# Ultrashort‐Peptide‐Responsive Gene Switches for Regulation of Therapeutic Protein Expression in Mammalian Cells

**DOI:** 10.1002/advs.202309411

**Published:** 2024-05-13

**Authors:** Jinbo Huang, Shuai Xue, Yu‐Qing Xie, Ana Palma Teixeira, Martin Fussenegger

**Affiliations:** ^1^ Department of Biosystems Science and Engineering ETH Zurich Klingelbergstrasse 48 Basel CH‐4056 Switzerland; ^2^ Faculty of Science University of Basel Klingelbergstrasse 48 Basel CH‐4056 Switzerland; ^3^ Present address: Key Laboratory of Growth Regulation and Translational Research of Zhejiang Province School of Life Sciences Westlake University Hangzhou Zhejiang China

**Keywords:** diabetes, gene circuits, gene switches, synthetic biology

## Abstract

Despite the array of mammalian transgene switches available for regulating therapeutic protein expression in response to small molecules or physical stimuli, issues remain, including cytotoxicity of chemical inducers and limited biocompatibility of physical cues. This study introduces gene switches driven by short peptides comprising eight or fewer amino acid residues. Utilizing a competence regulator (ComR) and sigma factor X‐inducing peptide (XIP) from *Streptococcus vestibularis* as the receptor and inducer, respectively, this study develops two strategies for a peptide‐activated transgene control system. The first strategy involves fusing ComR with a transactivation domain and utilizes ComR‐dependent synthetic promoters to drive expression of the gene‐of‐interest, activated by XIP, thereby confirming its membrane penetrability and intracellular functionality. The second strategy features an orthogonal synthetic receptor exposing ComR extracellularly (ComR_EXTRA_), greatly increasing sensitivity with exceptional responsiveness to short peptides. In a proof‐of‐concept study, peptides are administered to type‐1 diabetic mice with microencapsulated engineered human cells expressing ComR_EXTRA_ for control of insulin expression, restoring normoglycemia. It is envisioned that this system will encourage the development of short peptide drugs and promote the introduction of non‐toxic, orthogonal, and highly biocompatible personalized biopharmaceuticals for gene‐ and cell‐based therapies.

## Graphical abstract

Regulation of therapeutic protein expression by ultrashort peptides. In this engineered mammalian cell system, an ultrashort peptide receptor in the outer membrane detects inducers. Activation of the receptor triggers a signal cascade, leading to expression and secretion of the protein‐of‐interest. Encapsulated cells implanted in mice implement this mechanism, regulating therapeutic protein production, offering targeted treatment for specific diseases.

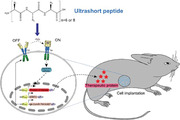

## 1 Introduction

Synthetic biology has ushered in a wealth of engineered transgene switches to govern therapeutic protein expression and cellular behavior for biomedical research.^[^ ^1^, ^2^, ^3^ ^]^ Numerous cell‐ and gene‐based therapies exploit engineered gene circuits to spatiotemporally regulate the production and delivery of biopharmaceuticals in response to an array of physical,^[^ ^4^, ^5^, ^6^ ^]^ chemical,^[^ ^7^, ^8^, ^9^, ^10^, ^11^ ^]^ and biological cues.^[^ ^2^, ^12^ ^]^ Physical inputs, such as light,^[^ ^4^ ^]^ heat,^[^ ^13^ ^]^ sound,^[^ ^14^ ^]^ and electricity,^[^ ^6^, ^15^ ^]^ have been employed for control, but often face issues such as complex control units, limited biocompatibility, and high time and energy requirements.^[^ ^4^, ^6^, ^14^, ^15^ ^]^ Meanwhile, although food‐derived small‐molecule‐responsive gene switches with favorable safety and bioavailability characteristics have been developed,^[^ ^3^, ^11^, ^16^, ^17^, ^18^ ^]^ conventional drug‐ or chemical‐induced systems can still be hampered by off‐target effects, cytotoxicity, and complex pharmacodynamics.^[^ ^3^, ^7^, ^19^ ^]^ While certain endogenous metabolites and disease biomarkers have shown promise as regulators of mammalian gene switches, their practical application in disease treatment and clinical translation is hindered by low bioavailability and a lack of orthogonality.^[^ ^2^, ^12^, ^19^ ^]^ Therefore, there is a need for the development of a transgene switch that is not only highly biocompatible and minimally cytotoxic, but also orthogonal, to enable precise control of therapeutic transgene expression.

Peptides exhibit considerable variability in length, ranging from a few amino acids to several tens of residues.^[^ ^20^, ^21^ ^]^ Their multifaceted roles in biological processes encompass functions as hormones,^[^ ^22^, ^23^ ^]^ signaling inducers,^[^ ^24^, ^25^ ^]^ neurotransmitters,^[^ ^26^ ^]^ among others.^[^ ^20^, ^25^ ^]^ Peptides offer considerable advantages as potential pharmaceutical agents or triggers of transgenic switches in synthetic biology for biomedical applications.^[^ ^20^, ^27^ ^]^ Compared to proteins, the smaller size of peptides confers structural simplicity, rendering them more amenable to synthesis, modification, and scale‐up production during drug development.^[^ ^20^, ^28^, ^29^ ^]^ Moreover, peptides often show higher bioavailability than proteins due to a greater ability to traverse cell membranes, efficiently reaching their target sites when employed as drugs or supplements.^[^ ^20^, ^28^, ^30^ ^]^ Furthermore, peptides are often associated to diminished immunogenic responses compared to larger proteins,^[^ ^20^, ^28^, ^31^ ^]^ generally display superior biocompatibility and orthogonality, and reduced cytotoxicity and off‐target effects compared to conventional drugs or chemicals,^[^ ^19^, ^20^, ^28^, ^32^, ^33^ ^]^ arising in part from their capacity to undergo metabolism to amino acids, which can be readily assimilated by living cells. Consequently, the development of transgene circuits induced by small peptides holds tremendous potential for advancing personalized healthcare.

Here, we focused on *Streptococcus vestibularis* (*S. vestibularis*), a bacterium inhabiting the human oral cavity, which employs the communication regulator (ComR) and the sigX/comX‐inducing peptide (XIP) effector, a transcription initiation factor, to govern the expression of genes associated with genetic competence and quorum sensing‐related functions.^[^ ^34^, ^35^, ^36^, ^37^ ^]^ XIP, an autoinducing peptide, consists of just eight amino acid residues and is synthesized when the bacterial population attains a certain density threshold.^[^ ^34^, ^36^, ^37^ ^]^ Accumulated XIP in the cellular environment is internalized into the cytoplasm of recipient bacteria, where it activates the transcriptional regulator ComR. This activation entails dimerization of ComR, enabling it to bind to its promoter sequences, thereby initiating the expression of proteins linked to competence and quorum sensing.^[^ ^34^, ^35^, ^37^ ^]^

To develop a short‐peptide‐induced transgene switch in mammalian cells, we designed two distinct strategies for expressing a gene of interest (GOI) through the engineering of the ComR‐XIP system. In the first design, we concurrently expressed ComR fused with mammalian transactivators, and strategically integrated ComR binding operators upstream of a human‐secreted alkaline phosphatase (SEAP), an engineered thermally stable secreted reporter protein that can be quantified in the culture medium to indicate the promoter activity and the degree of induction, providing a non‐invasive and quantitative method for studying the dynamics of gene expression in living cells. Profiling of SEAP expression revealed that cells engineered with the XIP‐sensing system exhibited a half‐maximal effective concentration (EC50) of 99.4 µm when exposed to XIP. This observation underscores the ability of XIP to traverse the mammalian cell membrane. In the second design, with the aim of enhancing sensitivity, we constructed an orthogonal synthetic receptor exposed to the extracellular space (ComR_EXTRA_) to engage with inducers, subsequently activating downstream gene expression via intricately engineered intracellular cascades. Through phenotypic profiling of a randomly generated mutant library, we crafted the ComR_EXTRA_‐based gene switch (XIP_EXTRA_) with unparalleled responsiveness to short peptides in mammalian cells. Our investigations revealed that XIP_EXTRA_ exhibited orthogonal sensitivity to peptides containing six and eight residues, with EC50 values of 31.7 and 4.3 µm, respectively. Crucially, these two peptides elicited no immunogenic response in mice. In a proof‐of‐concept study, we demonstrated that the administration of these two short peptides independently triggered microencapsulated engineered monoclonal human cells stably expressing the best‐performing ComR_EXTRA_‐based system to produce and release sufficient insulin to restore normoglycemia in a mouse model of type‐1 diabetes (T1D). We believe that this system represents a significant advance in the development of gene therapy for personalized medicine, characterized by high biocompatibility, orthogonality, non‐cytotoxicity and non‐immunogenicity.

## 2 Results

### 2.1 Design and Validation of a Gene Switch Relying on XIP‐Mediated ComR‐DNA Binding

**Figure 1:**
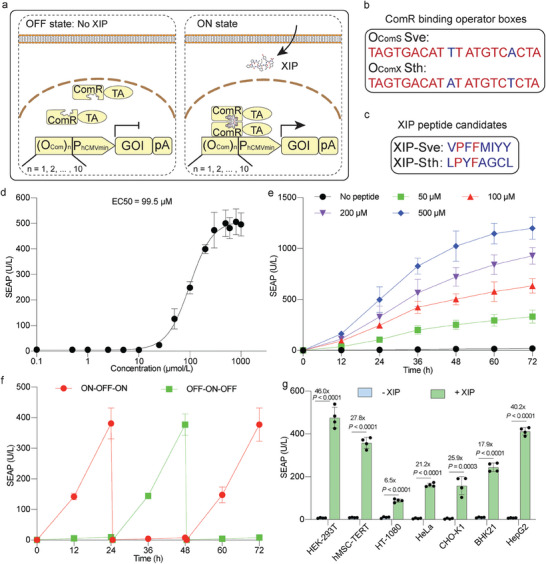
Design and characterization of a short‐peptide‐activated transgene expression switch in mammalian cells. a) Schematic representation of the genetic components required for constructing a short‐peptide‐induced gene switch in mammalian cells. In the absence of XIP, constitutively expressed ComR fused with a mammalian TA domain is not able to bind to the cognate DNA binding sites (O_Com_) upstream of a human minimal promoter (P_hCMVmin_), and therefore the expression of the downstream reporter is turned off (OFF). Upon XIP binding, ComR‐TA dimerization occurs, enabling its interation with O_Com_ and subsequent activation of the expression of the GOI. Various synthetic promoters containing one to ten repeats of O_Com_ are used. b) ComR binding sequences from the O_ComS_ operator from *S. vestibularis* (Sve) and the ComX operator (O_ComX_) from *S. thermophilus* (Sth). c) Sequence alignment of the XIP variants from Sve and Sth strains. d) Dose‐response curve of SEAP production by transiently transfected HEK‐293T cells treated with XIP for 24 h, indicating the EC50 value. e) Dose‐dependent SEAP production over a 72‐hour period in engineered cells exposed to XIP at the indicated concentrations. SEAP levels in culture medium were profiled every 12 h. f) Reversibility of the synthetic gene switch. Engineered cells were alternately cultured for 24 h intervals in XIP‐free (OFF) or XIP‐containing medium (200 µm) (ON). The culture medium was replaced and the cell density was readjusted every 24 h. g) SEAP production by various mammalian cell lines transiently transfected with the peptide‐sensing system. The SEAP levels were quantified in the culture supernatants. The corresponding cell viability profiles are shown in Figure S3 (Supporting Information). U/L: unit per liter; In (g), all treatment groups were induced with 250 µm XIP (+), and the non‐treated groups received an equivalent amount of vehicle (DMSO) (‐). Data are means ± SD. *n* = 4. *P* values in g were calculated versus the corresponding non‐induced control.

To build a short‐peptide‐inducible gene switch in mammalian cells, we leveraged the bacterial‐derived ComR‐XIP system. The DNA‐binding protein ComR was C‐terminally fused to a mammalian transactivator (TA). This fusion protein binds to ComR‐binding operator sites placed upstream of a GOI in the presence of XIP, thereby initiating transcription (**Figure** **1**a). To test the ability of ComR to regulate gene expression in mammalian cells, we designed a set of synthetic promoters consisting of several tandem repeats of ComR operator sites (O_Com_) upstream of a minimal version of human cytomegalovirus immediate early promoter (P_hCMVmin_) and a reporter gene encoding SEAP (Figure 1a). The synthetic promoters were based on operator boxes either from the *S. vestibularis* (Sve) ComS gene (O_ComS_), or from the *S. thermophilus* (*S. thermophilus*, Sth) ComX gene (O_ComX_) (Figure 1b). XIP variants from *S. vestibularis* (XIP‐Sve) and *S. thermophilus* (XIP‐Sth) were chemically synthesized and compared as inducers (Figure 1c).

First, we evaluated the different operator boxes and XIPs in transiently transfected human embryonic kidney (HEK‐293T) cells using ComR‐VP64 as the transactivator (pJH1504, P_hCMV_‐ComR‐VP64‐pA), in which VP64 consists of four tandem repeats of the *herpes simplex* virus early transcriptional activator VP16. Among the tested conditions, the most substantial SEAP fold‐activation was obtained with the reporter construct containing two repeats of the O_ComS_ site (pJH1530, O_2xComS_‐P_hCMVmin_‐SEAP‐pA) in cells treated with the XIP‐Sve peptide (Figure S1a–d and Table S1, Supporting Information). Thus, these conditions were selected for further optimizations. A comparison with two alternative transactivator variants, VP16 and a tripartite activator consisting of VP64, P65, and Rta (VPR), revealed that VPR showed the highest fold induction (Figure S1e, Supporting Information). We also replaced cysteine with serine residues in ComR, as previous studies showed this could enhance the stability and performance of the targeting protein.^[^ ^38^, ^39^, ^40^ ^]^ However, no significant increase in SEAP level or fold induction was observed in the mutant variants (Figure S1f, Supporting Information). Next, we screened additional promoter variants containing up to ten tandem repeats of the ComS operator sequence and found that nine repeats (pJH1543, O_ComS9_‐P_hCMVmin_‐SEAP‐pA) showed the best performance, considering both induction fold and overall SEAP production (Figure S2a, Supporting Information). Afterward, we employed the best reporter construct for determining the optimum molar ratio of the two gene‐switch components (pJH1522 and pJH1543). The highest SEAP fold‐induction was achieved at a ratio of 3:1 for ComR‐VPR (pJH1522) and O_ComS9_ (pJH1543), respectively (Figure S2b,c, Supporting Information).

Next, we obtained a dose‐response curve in HEK‐293T cells transiently transfected with the most effective switch constructs in order to evaluate the response to XIP. The results revealed that XIP induced SEAP expression with an EC50 of 99.4 µm (Figure 1d), highlighting its penetration ability across the mammalian cell membrane, presumably due to its simple structure and low molecular mass. Moreover, SEAP exhibited continuous accumulation over time, with higher concentrations of XIP leading to increased SEAP production over a 72‐h period (Figure 1e). To assess the reversibility of the gene switch, we alternated engineered cells between XIP‐containing (ON state) and XIP‐free (OFF state) media at 24‐h intervals. The system exhibited robust reversibility, maintaining similar induction and repression profiles over multiple cycles of ON‐to‐OFF and OFF‐to‐ON switching (Figure 1f). In addition, we tested the versatility of the system by transiently transfecting a set of mammalian cell lines. While variations in expression level and induction fold were observed among different cell lines upon XIP treatment, the functionality of the system was validated accross all tested cell lines, including human mesenchymal stem cell‐derived hMSC‐TERT and liver‐derived HepG2 cells (Figure 1g; Figure S3, Supporting Information). These results indicate the broad compatibility of the XIP‐inducible system, emphasizing its potential for diverse applications.

### 2.2 Designing a XIP‐Inducible System Relying on Activation of Plasma Membrane Synthetic Receptors

**Figure 2:**
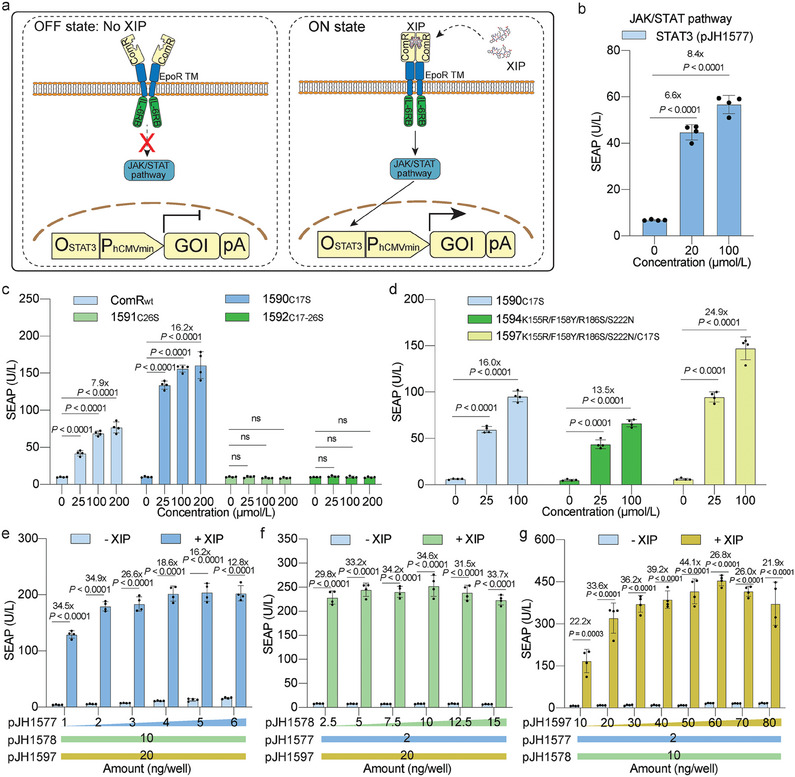
Design of the ComR_EXTRA_‐based short‐peptide‐sensing system. a) Schematic illustration of the ComR_EXTRA_‐based synthetic gene circuit for short peptide sensing in mammalian cells. ComR is displayed extracellularly by fusion to a synthetic receptor composed of the erythropoetin transmembrane domain (EpoR TM) linked to an intracellular signaling transduction domain (IL‐6RB). In the absence of XIP, the synthetic ComR receptor cannot dimerize, preventing activation of the downstream signaling pathway, and therefore the STAT3 operator‐controlled GOI is turned off (OFF). Upon XIP binding, ComR‐mediated receptor dimerization and reorientation of the receptor scaffold occur, leading to the expression of the downstream GOI. EpoR TM, erythropoietin receptor transmembrane domain; JAK/STAT, Janus kinase/signal transducer and activator of transcription; IL‐6RB, interleukin 6 receptor B. b) SEAP levels 24 h after incubation with XIP in the culture supernatant of HEK‐293T cotransfected with the receptor ComR plasmid (pJH1567, P_SV40_‐SP‐ComR‐EpoR‐TM‐IL‐6RB‐pA), the reporter plasmid containing STAT3 operator‐controlled SEAP (pJH1577, O_STAT3_‐P_hCMVmin_‐SEAP‐pA) and the enhancing plasmid constitutively expressing STAT3 (pJH1578, P_hCMV_‐STAT3‐pA). c) Cysteine mutagenesis screening of the ComR receptor. SEAP production was quantified after XIP induction for 24 h. C17S, cysteine residue at the 17th position mutated to serine; C26S, cysteine residue at the 26th position mutated to serine; C17‐26S, both cysteine residues mutated to serine residues. d) Secondary screening of ComR variants obtained from error‐prone PCR and/or combined with cysteine mutation from (c). e–g) Molar ratio optimization among reporter plasmid (pJH1577), enhancing plasmid (pJH1578) and ComR receptor plasmid (pJH1597, P_SV40_‐SP‐ComR_K155R/F158Y/R186S/S222N/C17S_‐EpoR‐TM‐IL‐6RB‐pA). The peptide‐sensing system was transiently transfected with increasing amounts of one plasmid, and constant amounts of the other two: e), constant amounts of pJH1578 and pJH1597; f), constant amounts of pJH1577 and pJH1597; g), constant amounts of pJH1577 and pJH1578. The indicated amounts of pJH1577, pJH1578, and pJH1597 were applied per well of a 96‐well plate cell culture in (e), (f), and (g), respectively. In (e–g), all treatment groups were incubated with 50 µm XIP (+), and the non‐treated groups received an equivalent amount of vehicle (DMSO) (‐). All data are means ± SD; *n* = 4. *P* values in (c–g) were calculated versus the corresponding non‐induced control. ns means not significant (*p‐*values > 0.05).

In our pursuit of a more sensitive XIP‐inducible system, we next explored the concept of extracellular XIP‐induced dimerization of ComR, circumventing the need for XIP to penetrate the cell membrane. To achieve this, we fused ComR C‐terminally to the transmembrane domain of the Epo receptor (EpoR TM),^[^ ^41^ ^]^ thereby exposing ComR to the extracellular space (ComR_EXTRA_) (**Figure** **2**a; Figure S4a, Supporting Information). In this configuration, the ComR protein was linked to the N‐terminus of EpoR TM, and multiple intracellular signal transduction domains were C‐terminally fused to EpoR TM to identify the most effective activated downstream signaling pathways, including JAK/STAT (Janus kinase/signal transducer and activator of transcription; induced by interleukin 6 receptor B (IL‐6RB)) (pJH1567, P_SV40_‐igk‐ComR‐EpoRTM‐IL6RB‐pA), PI3K/Akt (phosphatidylinositol 3‐kinase/protein kinase B; induced by vascular endothelial growth factor receptor 2 (VEGFR2)) (pJH1569, P_SV40_‐igk‐ComR‐EpoRTM‐VEGFR2‐pA) and PLCG (phospholipase C gamma; induced by VEGFR2) (pJH1569), as well as MAPK (mitogen‐activated protein kinase; induced by fibroblast growth factor receptor 1 (FGFR1)) (pJH1568, P_SV40_‐igk‐ComR‐EpoRTM‐FGFR1‐pA)^[^ ^41^ ^]^ (Figure 2a; Figure S4a and Table S1, Supporting Information). We found that the JAK/STAT signaling pathway, a fundamental cellular signaling cascade involved in the regulation of immune responses, cell growth, and differentiation, afforded the highest fold‐induction in the presence of XIP when its enhancer STAT3 (pJH1578, P_hCMV_‐STAT3‐pA) was co‐expressed (Figure 2b; Figure S4b–d, Supporting Information), which was consistent with the initial design.^[^ ^41^ ^]^ Therefore, the receptor based on JAK/STAT signaling pathway activation was selected for further optimizations. First, we tested the ComR variants in which we had point‐mutated cysteine to serine residues, a modification that afforded improved performance in previous studies,^[^ ^38^, ^39^, ^40^ ^]^ and found that in this configuration the mutant ComR_C17S_ significantly increased the fold‐induction and expression levels, while the other single mutant, ComR_C26S_, or the double mutant ComR_C17‐26S_ failed to respond to XIP (Figure 2c). In parallel, we conducted error‐prone PCR‐based random mutations on ComR and evaluated the resulting library in a high‐throughput functional screening assay by transfection into HEK‐293T cells cultured in 96‐well plates (Figure S5a, Supporting Information). SEAP levels in the culture supernatant of HEK‐293T cells expressing either the wild‐type ComR_wt_‐ (pJH1567) or the mutant ComR_mut_‐based receptor switches (pJH1567‐ComR_mut_, Figure S5a, Supporting Information) were measured after 24 h in the presence or absence of XIP (Figure S5b, Supporting Information). The six best mutant hits, were selected for further validation in a secondary screening. The ComR_E1_ variant containing 4 mutations (ComR_K155R/F158Y/R186S/S222N_) significantly improved both the fold‐induction and the expression level obtained with the wild‐type ComR (Figure S5c, Supporting Information). Subsequently, we introduced the point mutation C17S into this variant and observed a synergistic effect in the resulting 5‐residue mutant, achieving a 24.9‐fold induction at 100 µm XIP (pJH1597, P_SV40_‐igk‐ComR_E1/C17S_‐EpoR TM‐IL‐6RB‐pA) (Figure 2d). Fine‐tuning the mass ratio of the three‐component switch revealed that the highest fold‐induction in SEAP expression (44.1‐fold) was obtained at 2:10:50 for reporter (pJH1577), constitutive expression of STAT3 (pJH1578) and receptor (pJH1597), respectively (Figure 2e–g). To validate the extracellular presentation of the engineered ComR, we initially fused a fluorescent protein, EGFP, to the C‐terminus of ComR (pJH1687, P_SV40_‐SP‐ComR‐EGFP‐GS4‐TM‐IL‐6RB‐pA) (Figure S6a, Supporting Information) and performed fluorescence‐microscopic imaging. The results confirmed that the ComR was indeed integrated into the extracellular membrane (Figure S6b, Supporting Information). For further confirmation, we introduced an immunogenic hemagglutinin (HA) tag N‐terminally in ComR (pJH1692, P_SV40_‐SP‐3xHA‐ComR‐GS4‐TM‐IL‐6RB‐pA) (Figure S6a, Supporting Information). The resulting cells were immunostained with a fluorescent anti‐HA antibody and analyzed by flow cytometry. The results again demonstrated that the ComR domain was presented on the outer membrane of cells, where it would facilitate XIP recognition (Figure S6c–f, Supporting Information).

### 2.3 Characterization and Validation of the ComR_EXTRA_‐Based Short‐Peptide‐Inducible Gene Switch

**Figure 3:**
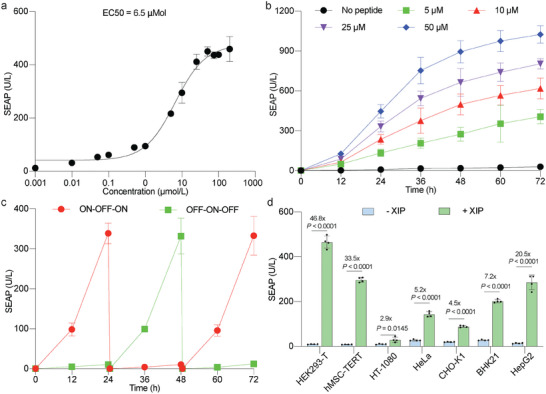
Characterization of the ComR_EXTRA_‐based short‐peptide‐sensing system. a) Characterization of the dose‐response curve of SEAP production by HEK‐293T cells transiently cotransfected with the ComR_EXTRA_‐based short‐peptide‐induced system. Cells were treated with different concentrations of XIP, and the EC50 value was determined after 24 h of inducer addition. b) Dose‐dependent SEAP production kinetics over a 72‐hour period in ComR_EXTRA_‐engineered cells exposed to XIP at the indicated concentrations. The SEAP levels were quantified in the culture supernatants at the indicated time points. c) Reversibility of ComR_EXTRA_‐engineered cells expressing SEAP. Engineered cells were alternately cultured for 24‐h cycles in a medium containing XIP at a concentration of 25 µm (ON) or XIP‐free medium (OFF). Culture supernatant samples were collected every 12 h for analysis of SEAP production. The culture medium was exchanged and the cell density was re‐adjusted every 24 h. d) SEAP produced by different mammalian cell lines transiently transfected with the ComR_EXTRA_‐based short‐peptide‐induced system. The treatment groups were induced with 50 µm XIP (+), and the non‐treated groups received an equivalent amount of vehicle (DMSO) (‐). The corresponding cell viability profiles are shown in Figure S6 (Supporting Information). In (a), (c), and (d), SEAP levels were measured 24 h after induction in the culture supernatants. All data are means ± SD; n = 4. *P* values in (d) were calculated versus the corresponding non‐induced control.

The dose‐response curve for XIP‐induced reporter expression displayed a nearly linear relationship from 1 to 50 µm, with an EC50 of 6.5 µm (**Figure** **3**a), marking a significant 15.3‐fold decrease compared to the EC50 of the previously designed gene switch (Figure 1e). When assayed at various doses of XIP, the HEK‐293T cells transiently transfected with the ComR_EXTRA_‐based system exhibited a steady, linear increase in SEAP protein secretion during the exponential growth phase, and the expression kinetics were maintained for at least 72 h (Figure 3b). This gene switch displayed robust reversibility, responding effectively to cycles of exposure (ON) and withdrawal (OFF) of XIP at 24 h intervals (Figure 3c). Furthermore, when the system was overstimulated by XIP peptide, we observed no significant difference of cell viability between the control and treatment groups (Figure S7a,b, Supporting Information). On addition, although the ComR_EXTRA_ system was engineered based on IL‐6RB, addition of IL‐6 to the transfected cells showed no apparent induction compared to the control group (Figure S7c, Supporting Information).

The efficacy of the ComR_EXTRA_‐based system was similarly validated across all tested cell lines (Figure 3d; Figure S8, Supporting Information), including hMSC‐TERT and HepG2 cells, requiring lower concentrations of XIP to reach reporter expression levels comparable to those of the previous system (Figure 1g). These results underscore the system's broad compatibility and versatile applicability, enabled by XIP induction. Considering the fold‐inductions and the basal and maximal expression levels, we selected HEK‐293T cells for subsequent experiments and designated this ComR_EXTRA_‐based gene switch as XIP_EXTRA_ system.

### 2.4 Generation and Characterization of a XIP_EXTRA_ Monoclonal Cell Line

**Figure 4:**
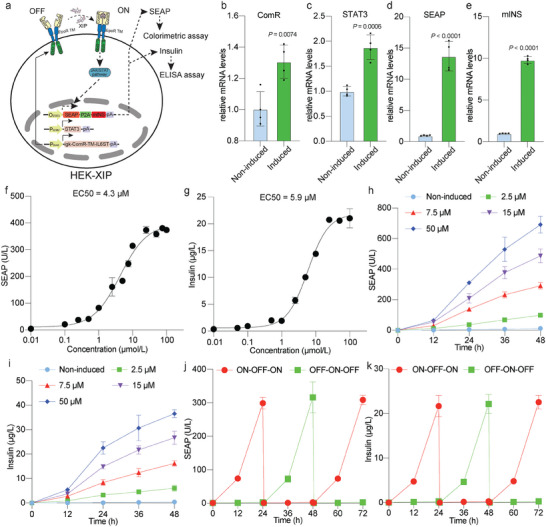
In vitro characterization of the ComR_EXTRA_‐based monoclonal cell line HEK‐XIP. a) Schematic depiction of stably transfected HEK‐XIP cells expressing SEAP and insulin following XIP peptide induction. In the presence of XIP, ComR‐mediated receptor dimerization and reorientation of the receptor scaffold ensue, leading to the expression of the downstream genes of interest (GOIs), including SEAP and mINS. The expression levels of SEAP and mINS were quantified using appropriate measurement kits. b–e) Relative mRNA levels of ComR (b), STAT3 (c), SEAP (d) and mINS (e) in HEK‐XIP monoclonal cells in the absence of XIP (Non‐induced) and the presence of XIP (Induced) at a concentration of 25 µm. The cells were harvested after 24 h of incubation with or without XIP. The mRNA levels were analyzed by quantitative real‐time PCR. f,g) Characterization of the dose‐response curves of SEAP (f) and insulin (g) of the stable monoclonal cell line HEK‐XIP cultured in the presence of different XIP concentrations after 24 h of incubation. EC50 values are indicated. h,i) Dose‐dependent SEAP (h) and insulin (i) expression kinetics over a 48‐hour period in HEK‐XIP monoclonal cells exposed to XIP at the indicated concentrations. The SEAP and insulin levels were quantified in the culture supernatants at the indicated time points. j,k) Reversibility of SEAP (j) and insulin (k) expression by HEK‐XIP monoclonal cells. HEK‐XIP cells were alternately cultured for 24‐h cycles in medium containing XIP at a concentration of 25 µm (ON) or XIP‐free medium (OFF). Culture supernatant samples were collected every 12 h for analysis of SEAP and insulin production. The culture medium was exchanged and the cell density was re‐adjusted every 24 h. All data are means ± SD; *n* = 4. The *p‐*values indicate the significance of differences in the mean values versus the non‐induced group.

For a proof‐of‐principle study in vivo, we chose the prevalent chronic disease T1D as a target.^[^ ^42^ ^]^ For this purpose, we first established a stable cell line with the XIP_EXTRA_ system genomically integrated, in which the therapeutic protein (mouse insulin, mINS) was placed downstream of SEAP, employing a P2A self‐cleaving peptide strategy^[^ ^43^ ^]^ (Figure S9a, Supporting Information**;** **Figure** **4**a). The system components (pJH1611, ITR‐O_STAT3_‐SEAP‐P2A‐mINS‐pA:P_RPBSA_‐ECFP‐P2A‐PuroR‐pA‐ITR; pJH1618, ITR‐P_hCMV_‐STAT3‐pA:P_RPBSA_‐ECFP‐P2A‐PuroR‐pA‐ITR and pJH1632, ITR‐P_SV40_‐SP‐ComR_E1/C17S_‐EpoR‐IL‐6RB‐pA: P_hCMV_‐ZeoR‐P2A‐mRuby‐pA‐ITR) were semi‐randomly integrated into the genome of HEK‐293T cells using the Sleeping Beauty transposase system.^[^ ^44^ ^]^ Monoclonal cell lines were isolated by fluorescence‐activated cell sorting (FACS) (Figure S9b–i, Supporting Information) and screened by quantitative SEAP assay after two weeks of culture in the medium containing selective antibiotics (Figure S9j,k, Supporting Information). A secondary screening was further performed to select the cell clone (HEK‐XIP) with the highest induction fold and lowest basal expression of the SEAP reporter (Figure S10a,b, Supporting Information). Flow‐cytometry analysis confirmed the presence of the three fluorescence‐tagged XIP_EXTRA_ constructs in nearly 100% of the analyzed live HEK‐XIP cells (Figure S10c–j, Supporting Information).

We also carried out quantitative PCR (qPCR) to compare the transcript levels of ComR, STAT3, SEAP, and mINS in HEK‐XIP cells under induced and non‐induced conditions (Table S2, Supporting Information). While constitutively expressed ComR and STAT3 transcripts showed less than 2‐fold induction, SEAP and mINS transcripts were significantly upregulated in cells cultured in XIP‐containing medium (Figure 4b–e), further confirming the inducibility of the ComR_EXTRA_‐based HEK‐XIP cell line. To evaluate HEK‐XIP sensitivity, we monitored the expression of both SEAP and insulin after induction with increasing doses of XIP. The XIP‐induced SEAP expression exhibited an EC50 of 4.3 µm (Figure 4f), while insulin expression exhibited an EC50 of 5.9 µm (Figure 4g), which were consistent with the findings in transiently transfected HEK‐293T cells (Figure 3a). HEK‐XIP responded within 3 h post‐XIP addition, yielding significantly increased amounts of SEAP protein levels compared to non‐induced cells (Figure S11, Supporting Information). Furthermore, HEK‐XIP cells showed dose‐ and time‐dependent SEAP (Figure 4h) and insulin (Figure 4i) expression kinetic profiles over a consecutive 48 h period, in accordance with the results in the transiently transfected system (Figure 3b). Reversibility tests showed that HEK‐XIP cells could be activated by XIP addition and deactivated by XIP washout at 24 h intervals for both SEAP (Figure 4j) and insulin (Figure 4k), indicating excellent reversibility. Additionally, even after the XIP peptide was removed following incubation for 6 or 12 h, HEK‐XIP cells retained ≈3.7% and 23.6% of their full activity at 72 h, respectively, compared to cells that underwent no medium changes (Figure S12, Supporting Information).

### 2.5 Specificity Assessment

**Figure 5:**
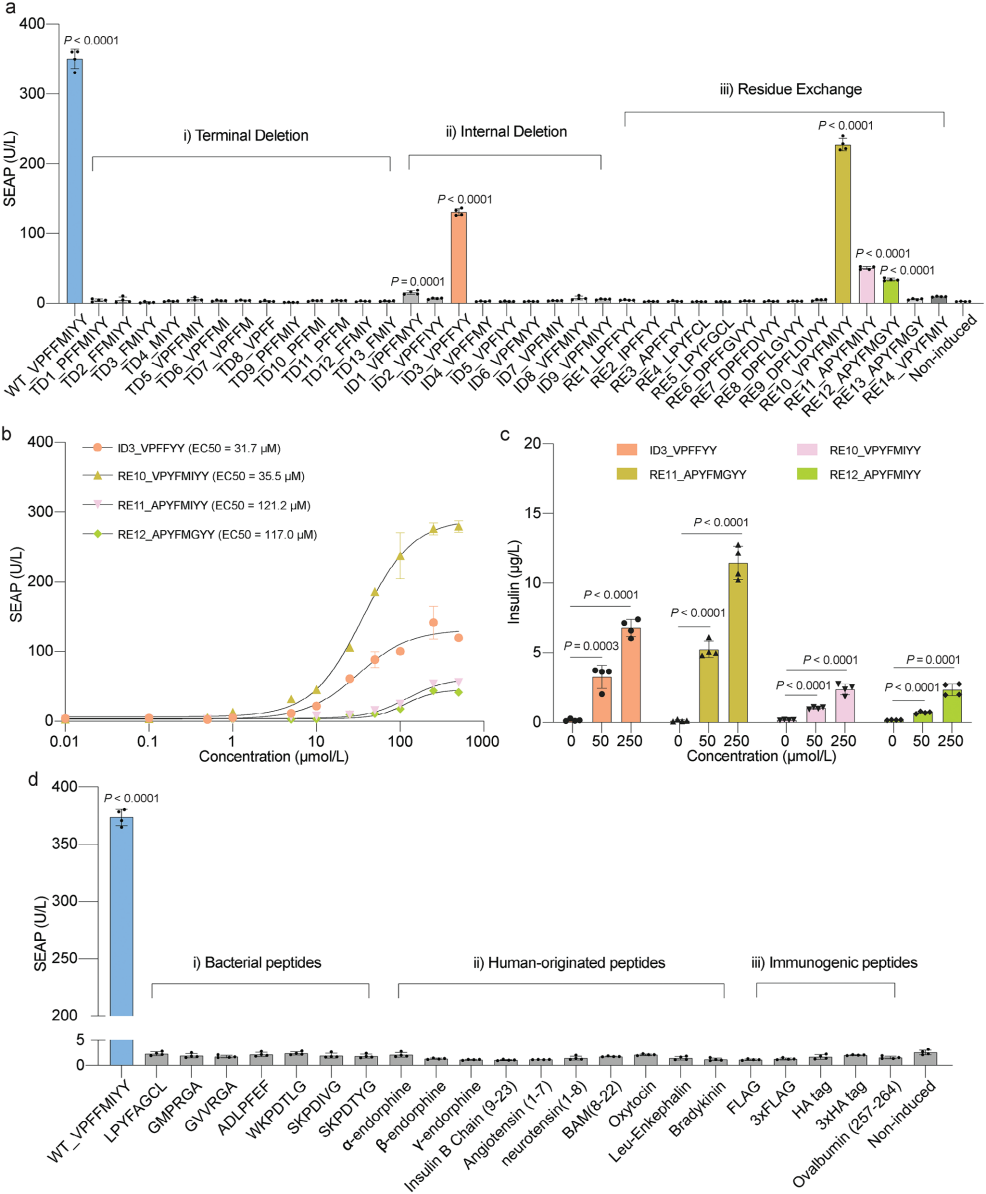
Specificity and orthogonality evaluation for HEK‐XIP cells. a) Specificity assessment by screening XIP variants. (i). Peptide screening for N‐terminal or C‐terminal residue deletion based on the wild‐type XIP (XIP_WT) peptide sequence, WT_VPFFMIYY. (ii). Peptide screening for internal residue deletion based on XIP_WT peptide. (iii). Peptide screening for exchanging and/or deleting residues based on XIP_WT peptide. TD, terminal deletion. ID, internal deletion. RE, residue exchange. b) Dose‐response curve of SEAP production in 24 h by HEK‐XIP monoclonal cell line cultured in the presence of peptide variants ID3, RE10, RE11 and RE12. The EC50 value is indicated for each peptide. c) Dose‐dependent insulin production in the culture supernatant of HEK‐XIP monoclonal cells after 24 h of incubation with the indicated peptides at the indicated concentrations. d) Specificity and orthogonality testing for HEK‐XIP cells. (i). Specificity testing for bacteria‐derived peptides. (ii). Orthogonality testing for human‐originated peptides. (iii). Specificity and orthogonality testing for widely used peptides. All data are means ± SD; *n* = 4. ns means not significant (*p‐*value > 0.05). The *p*‐values indicate the significance of the difference in the mean value versus the non‐induced group.

To evaluate the specificity of induction of HEK‐XIP cells by the XIP (VPFFMIYY), we performed a systematic screening, deleting internal residues and residues at both termini. Deletions in the N‐terminal or C‐terminal regions significantly impaired the induction functionality (**Figure** **5**a(i); Tables S3 and S4, Supporting Information). As for internal deletions (ID), two variants of XIP (ID1 and ID3) showed significant fold inductions compared to the non‐induced control (Figure 5a(ii); Tables S3 and S4, Supporting Information), with the 6‐residue ID3 variant showing the best performance. This motivated us to conduct a further residue exchange (RE) screening of both XIP and ID3 based on the structural similarity and conservation of amino acids (Figure 1c). Quantification of culture supernatants after 24 h of incubation showed significant fold inductions for 3 variants (Figure 5a(iii) and Table S3, Supporting Information). Among these, RE10, in which phenylalanine (F) was replaced by the relatively similar tyrosine (Y) at the third position (Figure 1c), afforded the highest fold‐induction. Nevertheless, the SEAP fold‐inductions achieved with ID3 and RE10 were still lower than that obtained with the wild‐type XIP (Figure 5a), with EC50 values higher than 30 µm (Figure 5b). Furthermore, HEK‐XIP cells cultured in the presence of these peptide analogs showed significantly elevated insulin production levels and limited cytotoxicity (Figure 5c; Figure S13, Supporting Information). Considering peptide size and inducibility, we selected wild‐type XIP and ID3 for subsequent experiments.

Next, we explored the possibility of conserved regions in mammalian proteins similar to the sequences of these short peptides. Using the NCBI human database (https://blast.ncbi.nlm.nih.gov/Blast.cgi) (Tax ID: 9606) we conducted sequence blast with XIP and ID3 as templates. The top twenty best‐matched genes for both peptides were selected and sequence alignments were performed (Figure S14a,b, Supporting Information). Short peptides based on conserved motifs (highlighted in green rectangles) identified from these alignments (Figure S14a,b, Supporting Information) were synthesized and tested in HEK‐XIP cells, but showed no induction after 24 h of incubation (Figure S14c,d, Supporting Information). These results highlight the specificity and orthogonality of the ComR_EXTRA_‐based short‐peptide‐induced system.

To test if the XIP short peptide can mediate cell‐cell communications, we engineered HEK‐293T cells as sender cells by C‐terminally fusing the XIP_WT sequence with a reporter protein under the control of a constitutive promoter (pJH1680, P_EF‐1α_‐NanoLuc‐GS1‐XIP‐pA). Interestingly, co‐culture experiments revealed a significant induction signal when HEK‐XIP cells were co‐cultured with the HEK‐293T cells transiently transfected with pJH1680 (Figure S15, Supporting Information), suggesting the potential application of XIP to facilitate cell‐cell communications. We also assessed the responsiveness of the system to natural short peptides originating from bacteria.^[^ ^36^ ^]^ SEAP analysis showed no significant fold‐inductions compared to the non‐induced control (Figure 5d(i)). Furthermore, we exposed HEK‐XIP cells to a number of human‐originated short peptides, including endorphins,^[^ ^45^ ^]^ C peptide,^[^ ^46^ ^]^ and BAM8‐22,^[^ ^47^ ^]^ and found that none of them displayed inducing activity (Figure 5d(ii)). Lastly, commercial immunogenic peptides^[^ ^48^ ^]^ failed to induce the XIP_EXTRA_ system, even at high dosages (Figure 5d(iii)). Collectively, these results highlight the specificity of HEK‐XIP cells to the inducer peptide.

### 2.6 Cytotoxicity and Immunogenicity Assessment of XIP‐Derived Peptides

In addition to having no significant impact on cell viability within the concentration range tested (Figures S1c,  S3, and S6, Supporting Information), XIP had a negligible effect on the growth kinetics of non‐transformed HEK‐293T cells (Figure S16a, Supporting Information), or on the overall recombinant protein production capacity of transiently transfected cells with constitutive expression of SEAP (pJH3, P_hCMV_‐SEAP‐pA) (Figure S16b, Supporting Information). These results collectively demonstrate the absence of cytotoxicity associated with XIP in HEK‐293T cells.

To assess the immunogenicity, we subcutaneously injected XIP and ID3 into mice in three doses at two‐week intervals (Figure S17a, Supporting Information). Five weeks after the completion of immunization, blood serum was taken for antibody analysis, and the spleen and draining lymph nodes were harvested for T‐cell response analysis. A colorimetric assay revealed no detectable antibodies’ response to XIP or ID3 after immunization (Figure S17b, Supporting Information), whereas HA peptide, a well‐known immunogenic peptide as the positive control, induced a significant antibody response (Figure S17b, Supporting Information). FACS analysis of intracellular cytokine staining revealed no peptide‐specific T‐cell response was detected at six weeks after the completion of immunization with XIP or ID3 in mice (Figure S17c–f, Supporting Information). These results indicate that these peptides were not immunogenic in the mammalian host.

### 2.7 Short‐Peptide‐Induced Insulin Expression for the Treatment of T1D

**Figure 6:**
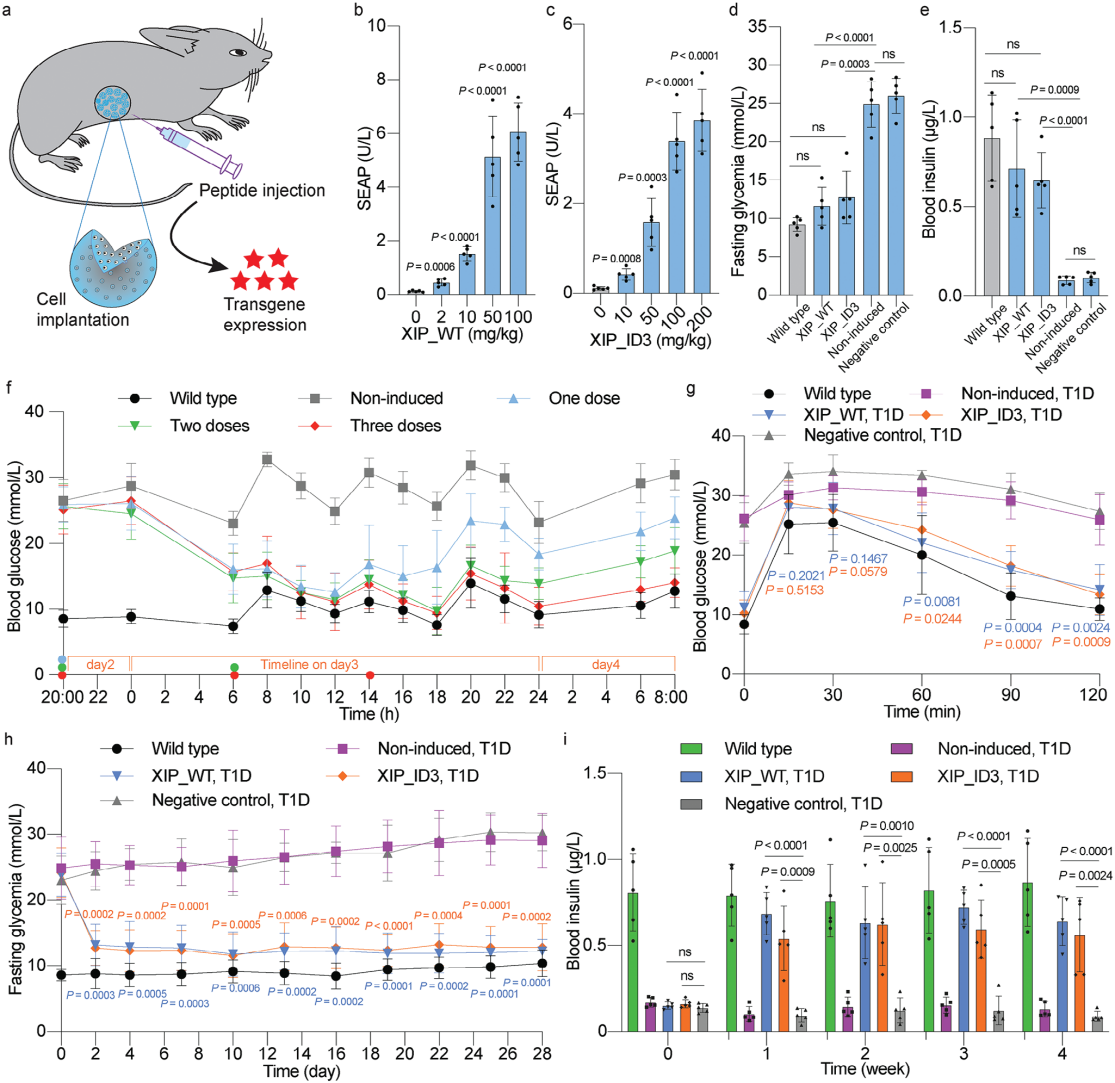
Functionality evaluation and validation of the ComR_EXTRA_‐based short‐peptide‐induced system in vivo. a) Scheme showing how encapsulated engineered monoclonal HEK‐XIP cells implanted intraperitoneally (i.p.) in mice are induced by short peptides, resulting in transgene expression. b,c) Dose‐dependent control of blood SEAP expression. Blood SEAP levels were profiled at two days after introduction of engineered cell implants in wild‐type mice administered (i.p.) with XIP_WT (b) and XIP_ID3 (c) at the indicated dosages. d,e) Fasting glycemia (d) and blood insulin (e) levels were measured after two days of treatment in type‐1 diabetic mice. Mice injected with the encapsulated HEK‐XIP cells were untreated (Non‐induced group) or treated with XIP_WT or XIP_ID3 at doses of 50 and 200 mg kg^−1^, respectively. Fasting glycemia was measured after 6 h of fasting. Blood was taken after fasting glycemia measurement. f) Dose course. T1D mice were implanted with encapsulated HEK‐XIP cells. XIP_WT was administered (i.p.) at one, two, or three doses per day (50 mg kg^−1^/dose) at the indicated time point(s), according to the scheme in Figure S14e (Supporting Information). Blood glucose levels were monitored for 36 h at the indicated time points. Time zero corresponded to midnight of day 2, and the blood glucose levels were profiled during day 3. g) Intraperitoneal glucose tolerance test was performed by administration of 1.5 g kg^−1^ aqueous D‐glucose to mice 3 days after implantation of microencapsulated cells and after fasting for 8 h. h) Fasting glycemia was recorded before implantation (day 0) and for four consecutive weeks after implantation in three groups of T1D mice with HEK‐XIP cell implants induced with XIP_WT, XIP_ID3 or vehicle (Non‐induced group). i) Blood insulin levels were quantified in wild‐type, negative control, XIP_WT‐induced XIP_ID3‐induced and non‐induced groups in 0 week (before implantation), and at 1, 2, 3, and 4 weeks after implantation. In (d–i), wild‐type and T1D mice without any implant or treatment were used as controls. All data are means ± SD; *n* = 5. ns means not significant (*p‐*value > 0.05) (versus non‐induced control or indicated group). The *p‐*values indicate the significance of differences in the mean values.

Prior to evaluating the in vivo performance of the short‐peptide‐induced system, we confirmed that microencapsulating the HEK‐XIP monoclonal cells in FDA‐licensed semipermeable alginate beads resulted in dose‐dependent production of SEAP and insulin upon incubation with XIP and ID3 (Figure S18a–d, Supporting Information). These beads allow free diffusion of nutrients, peptides, and therapeutic proteins, while also providing protection against the host immune system. To assess the in vivo performance, we first we administered the encapsulated HEK‐XIP cells intraperitoneally (i.p.) to wild‐type mice, which were then treated with different concentrations of short peptides (**Figure** **6**a). Analysis of blood samples indicated dose‐dependent induction of SEAP expression in mice treated with XIP (Figure 6b) or ID3 (Figure 6c). These results confirm the precise regulation of transgene expression by HEK‐XIP cells in vivo in response to these peptides.

Next, to examine the regulation of therapeutic protein expression, we implanted (i.p.) alginate‐encapsulated HEK‐XIP cells in experimental T1D mice. Treatment with the inducer peptide significantly attenuated fasting glycemia in the implanted TD1 mice, to levels comparable with the wild‐type levels (Figure 6d). Insulin blood levels were confirmed to be significantly increased in response to peptide addition (Figure 6e). To determine the optimal dosing regimen, we treated the mice with XIP up to three times per day, simulating the multiple daily insulin injections required for T1D patients, and repeated fasting–feeding cycles were scheduled to mimic human eating patterns (Figure S18e, Supporting Information). Interestingly, a single administration of peptide maintained normoglycemia for 18 h, while two or three doses maintained normoglycemia throughout the day (Figure 6f). Consequently, we opted for two doses per day in subsequent experiments.

An intraperitoneal glucose tolerance test performed on day 3 after implantation revealed that insulin production in the peptide‐induced group not only restored glucose homeostasis, but also attenuated postprandial glycemic excursions compared to control groups without any implants or with non‐induced implants (Figure 6g). Moreover, mice in both the XIP‐treated and ID3‐treated groups, receiving two doses per day, showed reduced fasting glycemia levels. Indeed, normoglycemia was achieved over the whole four‐week treatment period, with similar levels to the wild‐type group (Figure 6h). This was corroborated by weekly blood insulin profiling, which showed significantly higher insulin levels in XIP‐ and ID3‐induced animals compared to the non‐induced group (Figure 6i), Furthermore, profiling of inflammatory cytokines in the serum of the non‐induced and short‐peptide‐induced groups showed no significant differences (Figure S19, Supporting Information). Collectively, these data confirm the functionality of the XIP_EXTRA_ system for effectively treating T1D mice, without posing immunogenicity issues.

## 3 Discussion

We have engineered a highly sensitive, inducible transgene switch that enables the expression of therapeutic proteins in mammalian cells in response to small peptide inducers. Importantly, these inducers comprise just six to eight amino acids, and should be completely degraded into the component amino acids during cellular metabolism,^[^ ^49^, ^50^ ^]^ obviating any concerns regarding biocompatibility. Furthermore, our investigations unequivocally established the non‐cytotoxic and non‐immunogenic nature of these peptides in living organisms. Their structural simplicity should enable facile penetration of the cell membrane, and indeed, we found the eight‐residue XIP efficiently traverses the mammalian cell membrane to engage its operator, thereby activating transgene expression. Notably, presentation of the peptide receptor on the outer cell membrane significantly improved the inducer recognition efficiency. These small peptides can be easily synthesized using established chemical technology, and scale‐up should substantially lower the production cost, rendering them an economically viable option. We estimate the amount of peptide required for daily therapeutic dosing in a diabetic mouse to be ≈2.5 mg (2×1.25 mg), which would cost no more than 1.5 dollars (Table S4, Supporting Information). Notably, we confirmed that our short‐peptide‐induced system effectively regulates microencapsulated engineered cells in vivo, enabling the release of sufficient insulin to restore normoglycemia in a T1D mouse model. Compared with traditional small‐molecule‐triggered synthetic switches, short peptides offer the advantages of superior biocompatibility and minimal cytotoxicity.

While the XIP_EXTRA_ system indeed exhibits excellent excitable dynamics and expression kinetics in vitro in the presence of XIP peptide, for long‐term use in vivo, more frequent and higher doses of XIP would likely be needed to maintain a stable blood glucose level due to the complexity of the in vivo environment compared to cell culture medium. Furthermore, other potential limitations would need to be investigated before clinical trials. For example, natural peptides are easily hydrolyzed by endogenous proteases,^[^ ^33^, ^51^ ^]^ so it might be necessary to improve the stability and selectivity of XIP peptide for clinical application. various strategies could be employed for this purpose, including modification of residues terminally or internally,^[^ ^52^ ^]^ substitution of L‐amino acids by D‐amino acids,^[^ ^53^ ^]^ or incorporation of β‐amino acids.^[^ ^54^ ^]^

At the molecular level, we harnessed the competence‐regulating and quorum‐sensing components (ComR‐XIP) derived from the gram‐positive, human oral cavity inhabitant bacterium *S. vestibularis*.^[^ ^34^, ^37^ ^]^ Notably, *S. vestibularis* is categorized as a biosafety level 1 bacterium according to established biosafety protocols,^[^ ^55^ ^]^ mitigating concerns regarding the biosafety of these bacterial‐derived components. Of the two strategies we employed for engineering a peptide‐sensing system in mammalian cells, the XIP_EXTRA_ system, in which the ComR receptor is positioned extracellularly, rendered the engineered cells highly responsive to inducers. Furthermore, multiple rounds of mutagenesis of ComR yielded a substantial improvement in sensitivity compared to the initial design, achieving micromolar levels of EC50 in a monoclonal cell line that stably expressed the best‐in‐class ComR_EXTRA_‐based system. Importantly, a comprehensive battery of tests supported the specificity, orthogonality, non‐cytotoxicity and non‐immunogenicity of the XIP‐induced system. As regards specificity, although we found a number of proteins containing motifs akin to XIP or ID3, none of them induced reporter expression in HEK‐XIP cells after 24 h of incubation. Furthermore, high‐throughput screening of peptides from diverse sources, including mammals,^[^ ^45^, ^46^, ^47^ ^]^ bacteria,^[^ ^36^ ^]^ and commercially available peptides,^[^ ^48^ ^]^ indicated that the XIP_EXTRA_ system responds specifically to XIP and closely related peptides. We confirmed that these peptides are non‐cytotoxic to mammalian cells within a physiologically relevant concentration range and are non‐immunogenic in mice. Although we employed the IL‐6 receptor B and JAK/STAT3 signaling pathway in the XIP_EXTRA_ system, we confirmed that IL‐6 caused no detectable activation of the system. The reason for this is presumably that the IL‐6RB was engineered to reside on the cytoplasmic side. Even when cells were overstimulated or saturated with the peptide, interference with cellular homeostasis appeared to be negligible. It should be noted that when the engineered cells were implanted into mice, we micro‐encapsulated the cells, not only to protect the cells against the host's immune system, but also to reduce the risk of signal cross‐talk between engineered cells and the host. Therefore, we consider that the XIP_EXTRA_ system should have a minimal influence on cellular homeostasis both in vitro and in vivo.

While our proof‐of‐concept validation focused on the treatment of experimental T1D mice, we believe this approach has many potential applications for controlling therapeutic protein expression in the realm of personalized medicine. The advantages of these short‐peptide‐induced transgene switches include low cytotoxicity, low immunogenicity, and low cost of the inducer, as well as exceptional modularity and tunability, biocompatibility, biodegradability, specificity, and orthogonality. We believe this cell‐based, short‐peptide‐activated strategy will pave the way for the emergence of a range of next‐generation, non‐toxic gene‐switch platforms.

## 4 Experimental Section

### Plasmid Construction

All expression vectors were designed using Benchling (www.benchling.com) and details are provided in Table S1 (Supporting Information). The target DNA fragments were PCR‐amplified using Q5 High‐Fidelity 2x Master Mix (M0492L, New England BioLabs). Subsequently, these amplified DNA fragments were purified via electrophoresis and gel DNA recovery procedures employing a specialized kit from Zymo Research (D4002). All backbones were digested using restriction enzyme from New England BioLabs. Then ligation of backbones and inserts were performed by following the manufacturer's instructions for Gibson assembly (E2611L, New England BioLabs). The transformation was achieved by adding 50 µl of competent cells to the ligation mixture, followed by heat‐shocking at 42 °C for 90 s and spreading on a Lurie‐Bertani (LB)‐agar plate containing ampicillin. Bacteria were cultured for 16 h at 37 °C and a single colony was picked up for confirmation. All plasmids were verified by sequencing (Microsynth AG, Balgach, Switzerland) and propagated in competent XL10 gold K12 *E. coli* (Stratagene) in ampicillin‐supplemented LB lysogeny broth at 37 °C. Plasmid DNA was extracted using a Zyppy Plasmid Miniprep Kit (D4037, Zymo Research) or ZymoPURE II Plasmid Midiprep Kit (D4201, Zymo Research).

### Random Mutagenesis of ComR

Briefly, the ComR mutant library was PCR‐amplified using a GeneMorph II random mutagenesis kit (200552, Agilent Technologies) according to the manufacturer's protocol. Then the ComR mutants were gel‐recovered and cloned into pJH1567 (digested with *Bam*HI and *Bsp*EI). The library was transformed and propagated as described above. Monoclonal colonies were selected and cultured in 96‐deep‐well plates at 37 °C overnight. The plasmid library was extracted using a Zyppy‐96 Plasmid Kit (D4042, Zymo Research) following the manufacturer's protocol.

### Cell Culture and Transfection

Human embryonic kidney cells (HEK‐293T, ATCC: CRL‐11268), human telomerase‐immortalized mesenchymal stem cells (hMSC‐TERT),^[^ ^56^ ^]^ human fibrosarcoma cells (HT‐1080, ATCC: CCL‐121), human cervical adenocarcinoma cells (HeLa, ATCC: CCL‐2), Chinese hamster ovary cells (CHO‐K1, ATCC: CCL‐61), baby hamster kidney cells (BHK‐21, ATCC: CCL‐10), and human liver cancer cells (Hep G2, ATCC: CRL‐11997) were cultured in Dulbecco's modified Eagle's medium (DMEM, cat. no. 52100–39, Thermo Fisher Scientific) containing 10% fetal bovine serum (FBS, cat. no. F7524, lot no. 022M3395, Sigma‐Aldrich) and 1% (v/v) streptomycin/penicillin (cat. no. L0022, Biowest). All mammalian cells were grown at 37 °C in a humidified atmosphere containing 5% CO_2_. For all transient transfections, 15 000 cells were seeded per well of a 96‐well plate (cat. no. 3599, Corning Inc. Life Sciences) overnight. Cells were transfected by mixing 100 ng of DNA and polyethyleneimine (PEI, 24765‐2, Polysciences) in a ratio of 1:4. After incubation for >6 h, the culture medium was exchanged for an inducer‐free or inducer‐containing medium. The cell density was calculated using a cell counter (CellDrop BF Brightfield Cell Counter, DeNovix Inc.)

### Peptide Synthesis

All peptides used in this study were synthesized by NovoPro (Shanghai, China). The costs of synthesis are summarized in Table S4 (Supporting Information), and up‐to‐date information can be obtained from the supplier (https://novoprolabs.com/custom‐peptide‐synthesis/).

### Stable Cell Line Generation

HEK‐293T or hMSC‐TERT cells were seeded in a 24‐well plate at a density of 5× 10^4^ cells per well for 24 h. Then the cells were co‐transfected with 1000 ng of plasmid mixture containing pJH1611 (ITR‐O_STAT3_‐SEAP‐P2A‐mINS‐pA:P_RPBSA_‐ECFP‐P2A‐PuroR‐pA‐ITR), pJH1618 (ITR‐P_hCMV_‐STAT3‐pA:P_RPBSA_‐ECFP‐P2A‐PuroR‐pA‐ITR), pJH1632 (ITR‐P_SV40_‐SP‐ComR_K155R/F158Y/R186S/S222N/C17S_‐EpoR‐IL‐6RB‐pA: P_hCMV_‐ZeoR‐P2A‐mRuby‐pA‐ITR) and the Sleeping Beauty transposase pJH42 (P_hCMV_‐SB100X‐pA)^[^ ^6^, ^44^ ^]^ at the molar ratio of 2:10:50:6. After 24 h of transfection, the cells were transferred into the medium containing 0.5 µg ml^−1^ puromycin, 5 µg ml^−1^ blasticidin, and 100 µg ml^−1^ of zeocin for antibiotic selection for three days. Then the polyclonal population of engineered cells was suspended in DMEM medium and separated by fluorescence‐activated cell sorting (FACS, BD FACS ARIA FUSION, BD Biosciences) using yPET (517/530), mRuby (558/605), and iRFP (690/713) fluorescence. The triple‐positive population was isolated into single cells and further cultured in 96‐well plates in medium containing the three selection antibiotics. After two weeks of clonal expansion, monoclonal cell lines were screened by the addition of XIP and the best‐in‐class cell line was selected for follow‐up experiments. Fluorescence‐based flow cytometry was performed to confirm triple‐positive fluorescence for the best‐in‐class cell line.

### Effect of XIP on Cell Growth and Productivity

HEK‐293T cells were seeded in a 10 cm diameter dish at a density of 2.5 × 10^5^ cells/ml. After 24 h of culture, the cells were transfected with 35 µg of pJH3 (P_hCMV_‐SEAP‐pA) overnight, then resuspended and evenly distributed into two 24‐well plates. The treatment groups were treated with XIP at the indicated concentration. Samples were collected at successive time points for two days to quantify viable cells and SEAP production.

### Resazurin‐Based Cell Viability Assay

Cells were incubated with resazurin (cat. no. R7017, Sigma‐Aldrich, USA) at a concentration of 50 µg ml^−1^ for 2 h. Then fluorescence was assayed with a plate reader (Tecan Spark, Tecan Group Ltd., Switzerland) at excitation and emission wavelengths of 540 and 590 nm, respectively. The fluorescence intensity of non‐induced cells was set to 100%.

### SEAP Reporter Assay

SEAP reporter levels in cell culture supernatants were quantified using a colorimetric assay. In brief, 20 µl of cell culture supernatant was mixed with 80 µl of PBS buffer (cat. no. 14190144, ThermoFisher) and heat‐inactivated at 65 °C for 30 min. Then 80 µl of 2x SEAP assay buffer (20 mM homoarginine, 1 mm MgCl_2_, 21% diethanolamine, pH 9.8) was mixed with 20 µl of substrate solution, and 100 µl of the buffer‐substrate mixture was added to 100 µl of the diluted heat‐inactivated sample. Absorbance at 405 nm was recorded using a plate reader (Tecan Spark, Tecan Group Ltd., Switzerland) for 30 min at 37 °C to determine the SEAP levels.

### Nanoluciferase (NanoLuc) Reporter Assay

The Nano‐Glo Luciferase Assay System (cat. no. N1110, Promega, Wisconsin, USA,) was used to quantify the cell culture concentration of NanoLuc according to the manufacturer's protocol. Briefly, 5 µl of cell culture supernatant was mixed with 5 µl of buffer‐substrate mixture (at a ratio of 50:1) in a 384‐well plate (cat. no. 781076, Greiner BioOne, Kremsmünster, Austria). The plate was then centrifuged at 3000 g for 2 min to debubble samples. Luminescence was quantified in 20 min using a plate reader (Tecan Spark, Tecan Group Ltd., Switzerland).

### Co‐Culture of Mammalian Cells

Both sender and receiver cells were initially seeded in two separate 10‐cm dishes. Following 24 h of transfection, both cell populations were harvested, then resuspended, and the cell numbers were redetermined. Subsequently, the cells were mixed in a specified ratio and co‐seeded in 96‐well plates. Reporter signals were then quantified after 24 h of co‐culture.

### Insulin Quantification

Insulin concentrations in cell culture medium or blood serum were measured by an ELISA kit (cat. no. 10‐1247‐01, Mercordia, Uppsala, Sweden).

### Fluorescence‐Microscopic Analysis of ComR Localization

For microscopic examination, cells were seeded in black 24‐well plates (cat. no.: 211102, Cellvis, USA) and cultured in FluoroBrite DMEM medium (cat. no.: A1896701, ThermoFisher). Following 36 h of transfection, the nucleic acid stain Hoechst 33342 (cat. no.: H3570, ThermoFisher) was introduced to the cells at a working concentration of 1 µg ml^−1^ prior to imaging analysis. Imaging was conducted using a Nikon WF5 confocal microscope. Hoechst 33342 was excited with the 380 nm laser line, and emission was collected at 450 nm. The EGFP fluorescent proteins were analyzed using the 510/525‐545 channel.

### Cell Processing Prior to FACS Analysis of Anti‐3xHA Labeled Protein

HEK‐293T cells (5×10^5^) were transfected with targeting plasmids, incubated for 24 h, then harvested and rinsed twice with 1x PBS. Following this, the cells were incubated for 20 min at 4 °C with allophycocyanin (APC)‐labeled anti‐3xHA tag antibody (cat. no.: 901523, BioLegend) at a concentration of 2 µg mL^−1^, washed twice with 1x PBS and resuspended in 1x PBS buffer containing 0.1 µg mL^−1^ DAPI (cat. no.: D9542‐1MG, Sigma) for FACS. The DAPI (360/460) and APC (630/660) channels were utilized for cell analysis.

### RNA Extraction and qPCR

The cells were cultured in 10 cm dishes overnight, then harvested for total RNA extraction using a Quick‐RNA Miniprep Kit (Zymo Research, cat. no. R1054). The RNA samples were quantified using a NanoDrop 2000 (Thermo Fisher). Next, a cDNA library was established using a High‐Capacity cDNA Reverse Transcription Kit (Applied Biosystems, cat. no. 4368814). The cDNA was mixed with SYBR Green Supermix (Bio‐Rad, cat. no. 1725271) for qPCR analysis by QuantStudio 3 (Thermo Fisher). The *glyceraldehyde 3‐phosphate dehydrogenase* gene was used as a house‐keeping gene.

### Cell Encapsulation and Implantation in Mice

Cells were microencapsulated in 400 µm diameter alginate‐poly‐L‐lysine‐alginate beads using an encapsulator (Inotech Encapsulator IE‐50R, EncapBiosystems Inc., Switzerland) according to the manufacturer's protocol. The following parameters were set: a 20 mL syringe was operated at a flow rate of 400 units, a 200 µm nozzle was used with a vibration frequency of 1100 Hz, bead dispersion voltage was set at 1200 V and the stirrer speed was set at 60 rpm/min. The encapsulated cells were further cultured in complete DMEM for 36 h. Then the medium was replaced with serum‐free DMEM before implantation. Five million microencapsulated cells were intraperitoneally implanted through a standard 3 mL syringe (cat. no. 9400038, Becton Dickinson) equipped with a 0.7×30 mm needle (cat. no. 30382903009009, Becton Dickinson).

### Animal Experiments

Male and female C57BL/6JRJ mice were purchased from Janvier Labs (Le Genest‐Saint‐Isle, France). Eight‐week‐old wild‐type mice were used for immunogenicity assay, preliminary proof‐of‐concept testing, and generation of experimental type‐1 diabetic mice. Type‐1 diabetic mice were established by injecting streptozotocin (cat. no. S0130, Sigma‐Aldrich; 100 mg kg^−1^ in sodium citrate buffer adjusted to pH 4.3, 0.2 m) for three consecutive days. Mice were fasted for 8 h before each injection. Experimental type‐1 diabetes was confirmed after one week by profiling 8‐hour‐fasting blood glucose using a clinically licensed glucometer (Contour Next, Bayer Healthcare, Germany) and strips (cat. no. 5225293, Bayer Healthcare, Germany). Postprandial glycemic excursions were stimulated by intraperitoneally injecting 1.5 g kg^−1^ glucose, and the blood glucose levels were profiled at regular intervals over the following 2 h. For analysis of blood SEAP and insulin levels, blood serum was collected using Microtainer serum separator tubes (cat. no. 365967, Becton Dickinson). The blood was sampled from the tail or saphenous veins using a 20 µl glass micro‐haematocrit capillary (Avantor VWR, cat. no. 521–9100), then immediately transferred into a blood collection tube (BD Microtainer, cat. no.: BDAM365968) at room temperature for 10 min, followed by centrifugation at 6000 × g for 2 min. The supernatant serum was used for analysis or frozen at −80 °C within 30 min. Blood SEAP levels were quantified using a luminescence‐based assay kit (cat. no. ab133077, Abcam, Cambridge, UK). Blood insulin levels were profiled by an ELISA kit (cat. no. 10‐1247‐01, Mercordia, Uppsala, Sweden). All experiments involving animals were performed in accordance with the directives of the European Community Council (2010/63/EU), approved by the French Republic (project no. DR2018‐40v5 and APAFIS no. 16753) and carried out by Shuai Xue, Yu‐Qing Xie, Jinbo Huang, and Ghislaine Charpin‐El Hamri (no. 69266309) at the University of Lyon, Institut Universitaire de Technologie (IUT), F69622 Villeurbanne, France, or conducted by Shuai Xue according to the protocol (Protocol ID: 20‐009‐XMQ) approved by the Institutional Animal Care and Use Committee (IACUC) of Westlake University and in accordance with the Animal Care Guidelines of the Ministry of Science and Technology of the People's Republic of China.

### Immunization of Mice and Lymphocyte Isolation

Eight‐week‐old wild‐type male mice were immunized for peptide immunogenicity assessments. Briefly, peptide solution was premixed 1:1 with Freund's adjuvant in a total volume of 400 µl by thorough vigorous shaking. Then peptide/adjuvant mixes were injected subcutaneously in 30 min at a dose of 5 mg kg^−1^. Mice were immunized at 2‐week intervals. Negative controls (mock‐immunized) were also included in the study. One week after the third dose of immunization, blood was sampled for antibody analysis. One week later, mice were euthanized for dissection and organ collection. The spleen and draining lymph nodes (inguinal lymph nodes from both sides) were immediately collected for T cell response analysis and were preserved in 1 ml FACS buffer (PBS containing 0.2% w/v Bovine Serum Albumin (Merck) and 2 mm EDTA (Invitrogen)) on ice. For lymphocyte isolation, each spleen was transferred onto a cell strainer (pore size 70 µm, Corning) over a 50 ml conical tube, followed by grinding and mashing with a syringe plunger against the cell strainer. Cells were then flushed out from the cell strainer with cold FACS buffer and collected in a 50 ml conical tube. The tube was centrifuged at 1500 g for 2 min, and the cell pellet was resuspended in 2 ml ACK Lysing Buffer (Gibco) and incubated on ice for 5 min. Lysis was stopped by dilution with 10 ml cold FACS buffer, and the cells were centrifuged again at 1500 g for 2 min. The supernatant was discarded and the cell pellet was resuspended in 10 ml FACS buffer. Lymphocytes were isolated from the lymph nodes in the same manner, with the exception of ACK lysis, and finally resuspended in 1 ml FACS buffer. Aliquots of 200 µl of resuspended cells were taken for ex vivo stimulation with peptides and intracellular cytokine staining.

### Ex Vivo Stimulation of T Cells and Intracellular Cytokine Staining

Isolated lymphocytes from spleens and lymph nodes were placed in a U‐bottom 96‐well plate and resuspended in 100 µl complete RPMI‐1640 medium, which contains FBS (10% v/v), HEPES (pH 7.2∼7.5, 1% v/v, Gibco), penicillin/streptomycin (1% v/v, Gibco), sodium pyruvate (1% v/v, Gibco) and 2‐mercaptoethanol (0.1% v/v, Gibco). Cells were stimulated with 1 µm cognate peptides or with Cell Stimulation Cocktail (a cocktail of phorbol 12‐myristate 13‐acetate (PMA) and ionomycin, Invitrogen) as positive controls at 37 °C for 4 h, in the presence of 10 µm brefeldin A (MedChemExpress) to inhibit cytokine secretion. Next, the cells were washed with 150 µl FACS buffer, and centrifuged with 1,500 g for 2 min. The supernatant was discarded and the cells were blocked with anti‐CD16/32 antibody (1:100, 93, 101302, BioLegend) in 20 µl/well FACS buffer at 4 °C for 15 min. Then 20 µl/well antibody cocktail containing anti‐CD3ε (1:500, 17A2, 100306, BioLegend), anti‐CD4 (1:500, RM4‐5, 100526, BioLegend), anti‐CD8β (1:500, YTS256.7.7, 126606, BioLegend), and anti‐CD45 (1:500, S18009F, 157211, BioLegend) was added to the cell mixture for 20 min incubation at 4 °C. The incubated cells were washed with 200 µl PBS and the cell pellet was resuspended and incubated with Aqua Fixable Dye (1:1000 dilution, BioLegend) in 100 µl/well PBS at 4 °C for 30 min. The cells were washed twice with FACS buffer, then fixed and permeabilized with a Cytofix/Cytoperm Fixation/Permeabilization Solution Kit (cat: 51–2090KZ, BD Biosciences) for cytokine staining per the manufacturer's instructions. Briefly, the cell pellet was suspended and incubated with 100 µl/well Fixation/Permeable buffer at 4 °C for 20 min. After that, 150 µl/well 1x BD wash buffer was added and the cells were washed twice with 1x BD wash buffer, resuspended and incubated with anti‐IFN‐γ (1:200, XMG1.2, 505826, BioLegend) and anti‐TNF‐α (1:500, MP6‐XT22, 506308, BioLegend) in 20 µl/well 1x BD wash buffer at 4 °C for 30 min. Lastly, the cells were washed with 150 µl/well 1x BD wash buffer followed by 200 µl/well FACS buffer. Cells were kept in 200 µl/well FACS buffer before flow cytometry analysis. Flow cytometry data were collected using a CytoFLEX S Flow Cytometer with CytExpert Software v2.1 (Beckman Coulter) at the Single Cell Facility (SCF) of D‐BSSE. Analyses were performed using FlowJo 10.9 (Tree Star). Gate margins were determined on the basis of isotype controls and fluorescence‐minus‐one controls.

### Serum Inflammatory Cytokines

The concentrations of interferon gamma (IFN‐γ), interleukin‐6 (IL‐6), and tumor necrosis factor alpha (TNF‐α) in mouse serum were assessed by the use of specific enzyme‐linked immunosorbent assay (ELISA) kits. IFN‐γ levels were analyzed using the IFN gamma mouse ELISA kit (cat. no. ab282874, Abcam), IL‐6 levels were determined with the IL‐6 mouse ELISA kit (cat. no. ab100712, Abcam), and TNF‐α levels were quantified utilizing the TNF alpha mouse ELISA kit (cat. no. BMS607HS, ThermoFisher). Freund's Complete Adjuvant (cat. no.: 344289, Sigma‐Aldrich) and lipopolysaccharides (cat. no.: L2630‐10MG, Sigma‐Aldrich) were utilized to induce cytokine production as positive controls. Blood samples were collected from mice treated with adjuvant and LPS 24 hours post‐treatment. The quantification of cytokines from these groups was conducted using the same ELISA kit.

### Antibody Analysis for Peptide Immunogenicity

For antibody analysis, the peptides were coated on the EIA/RIA plate (cat. no. 3590, Corning Inc. Life Sciences) in carbonate coating buffer (cat. no. CB01100, ThermoFisher) at 4 °C overnight. The next day the plate was blocked with 20% nonfat milk (cat. no. A0830,1000, AppliChem Panreac) for 24 h at 4 °C, and incubated with antibodies at the indicated dilution or at 37 °C for 1 h. Next, horseradish peroxidase‐linked anti‐mouse IgG (cat. no. LNA931V, GE Healthcare, USA) was added to the plate at a dilution of 1:1000 for 1 h at 37 °C. Between each step, the plate was washed with PBS at least three times. Then, 100 µl ultra TMB substrate solution (cat. no. 34028, ThermoFisher) was added to the plate at room temperature for 15 min, followed by the addition of 50 µl of stop solution (2 m H_2_SO_4_). The OD450 was measured by a plate reader (Tecan Spark, Tecan Group Ltd., Switzerland). Mouse anti‐HA antibody (cat. no. 901523, BioLegend, USA, 1:1000 dilution) was used as a positive control.

### Statistics

Unless otherwise stated, all experiments were conducted with at least two independent biological replicates, and consistent results were obtained across these replicates. Details of data presentation, sample size of biological replicates (n), statistical analysis, and significance of differences are given in the figures and corresponding figure legends. The statistical significance of differences among multiple groups was evaluated with a two‐tailed, unpaired Student's *t*‐test or a one‐way analysis of variance (ANOVA) using GraphPad Prism (v 9.2.0, GraphPad Software Inc.) or Microsoft Excel (v 16.51, Microsoft Inc.).

## Conflict of Interest

The authors declare no conflict of interest.

## Author Contributions

J.H. and M.F. designed the project. J.H. performed the cell culture experiments. J.H., S.X., and Y.‐Q.X. performed the animal experiments. J.H. and Y.X. performed the immunogenicity assay. J.H., S.X., Y.‐Q.X., A.P.T., and M.F. designed the experiments and analyzed the results. J.H., S.X., Y.‐Q.X., A.P.T., and M.F. wrote the manuscript.

## Supporting information

Supporting Information

## Data Availability

The data that support the findings of this study are available from the corresponding author upon reasonable request.

## References

[1] A. Cubillos‐Ruiz , T. Guo , A. Sokolovska , P. F. Miller , J. J. Collins , T. K. Lu , J. M. Lora , Nat. Rev. Drug Discovery 2021, 20, 941. 34616030 10.1038/s41573-021-00285-3

[2] J. Manhas , H. I. Edelstein , J. N. Leonard , L. Morsut , Nat. Chem. Biol. 2022, 18, 244. 35058646 10.1038/s41589-021-00926-z PMC9041813

[3] M. Xie , M. Fussenegger , Nat. Rev. Mol. Cell Biol. 2018, 19, 507. 29858606 10.1038/s41580-018-0024-z

[4] M. Mansouri , M.‐D. Hussherr , T. Strittmatter , P. Buchmann , S. Xue , G. Camenisch , M. Fussenegger , Nat. Commun. 2021, 12, 3388. 34099676 10.1038/s41467-021-23572-4 PMC8184832

[5] M. Duque , C. A. Lee‐Kubli , Y. Tufail , U. Magaram , J. Patel , A. Chakraborty , J. Mendoza Lopez , E. Edsinger , A. Vasan , R. Shiao , Nat. Commun. 2022, 13, 600. 35140203 10.1038/s41467-022-28205-y PMC8828769

[6] J. Huang , S. Xue , P. Buchmann , A. P. Teixeira , M. Fussenegger , Nat. Metab. 2023, 5, 1395. 37524785 10.1038/s42255-023-00850-7 PMC10447240

[7] A. Bertschi , B.‐A. Stefanov , S. Xue , G. Charpin‐El Hamri , A. P. Teixeira , M. Fussenegger , Nucleic Acids Res. 2023, 51, e28. 36625292 10.1093/nar/gkac1256 PMC10018347

[8] C.‐Y. Wu , K. T. Roybal , E. M. Puchner , J. Onuffer , W. A. Lim , Science 2015, 350, aab4077. 26405231 10.1126/science.aab4077 PMC4721629

[9] C. Chen , G. Yu , Y. Huang , W. Cheng , Y. Li , Y. Sun , H. Ye , T. Liu , Nat. Chem. Biol. 2022, 18, 47. 34782743 10.1038/s41589-021-00899-z

[10] Y. Wang , S. Liao , N. Guan , Y. Liu , K. Dong , W. Weber , H. Ye , Sci. Adv. 2020, 6, eabb9484. 32821842 10.1126/sciadv.abb9484 PMC7413729

[11] L. Yang , J. Yin , J. Wu , L. Qiao , E. M. Zhao , F. Cai , H. Ye , Proc. Natl. Acad. Sci. USA 2021, 118, e2106612118. 34404729 10.1073/pnas.2106612118 PMC8403971

[12] M. Xie , H. Ye , H. Wang , G. Charpin‐El Hamri , C. Lormeau , P. Saxena , J. Stelling , M. Fussenegger , Science 2016, 354, 1296. 27940875 10.1126/science.aaf4006

[13] B. A. Stefanov , A. P. Teixeira , M. Mansouri , A. Bertschi , K. Krawczyk , G. C. E. Hamri , S. Xue , M. Fussenegger , Adv. Sci. 2021, 8, 2101813. 10.1002/advs.202101813 PMC8564464 34496151

[14] H. Zhao , S. Xue , M.‐D. Hussherr , P. Buchmann , A. P. Teixeira , M. Fussenegger , Lancet Diabetes Endocrinol. 2023, 11, 637. 37620062 10.1016/S2213-8587(23)00153-5

[15] K. Krawczyk , S. Xue , P. Buchmann , G. Charpin‐El‐Hamri , P. Saxena , M.‐D. Hussherr , J. Shao , H. Ye , M. Xie , M. Fussenegger , Science 2020, 368, 993. 32467389 10.1126/science.aau7187

[16] S. Galvan , O. Madderson , S. Xue , A. P. Teixeira , M. Fussenegger , Adv. Sci. 2022, 9, 2203193. 10.1002/advs.202203193 PMC9731693 36316222

[17] A. P. Teixeira , S. Xue , J. Huang , M. Fussenegger , Nucleic Acids Res. 2023, 51, e85. 37497781 10.1093/nar/gkad600 PMC10450161

[18] J. Yin , L. Yang , L. Mou , K. Dong , J. Jiang , S. Xue , Y. Xu , X. Wang , Y. Lu , H. Ye , Sci. Transl. Med. 2019, 11, eaav8826. 31645456 10.1126/scitranslmed.aav8826

[19] M. Siddiqui , C. Tous , W. W. Wong , Curr. Opin. Biotechnol. 2022, 78, 102823. 36332343 10.1016/j.copbio.2022.102823 PMC9951109

[20] V. Apostolopoulos , J. Bojarska , T.‐T. Chai , S. Elnagdy , K. Kaczmarek , J. Matsoukas , R. New , K. Parang , O. P. Lopez , H. Parhiz , Molecules 2021, 26, 430. 33467522 10.3390/molecules26020430 PMC7830668

[21] M. Su , Y. Ling , J. Yu , J. Wu , J. Xiao , Front. Genetics 2013, 4, 286. 10.3389/fgene.2013.00286 PMC3864261 24379829

[22] H. S. Tager , D. F. Steiner , Annu. Rev. Biochem. 1974, 43, 509. 4368999 10.1146/annurev.bi.43.070174.002453

[23] Y. Matsubayashi , Y. Sakagami , Annu. Rev. Plant Biol. 2006, 57, 649. 16669777 10.1146/annurev.arplant.56.032604.144204

[24] F. G. Chirdo , S. Auricchio , R. Troncone , M. V. Barone , Int. Rev. Cell Molecular Biol. 2021, 358, 165. 10.1016/bs.ircmb.2020.10.003 33707054

[25] G. J. Lyon , R. P. Novick , Peptides 2004, 25, 1389. 15374643 10.1016/j.peptides.2003.11.026

[26] S. H. Snyder , R. B. Innis , Annu. Rev. Biochem. 1979, 48, 755. 38738 10.1146/annurev.bi.48.070179.003543

[27] T. Uhlig , T. Kyprianou , F. G. Martinelli , C. A. Oppici , D. Heiligers , D. Hills , X. R. Calvo , P. Verhaert , EuPA Open Proteomics 2014, 4, 58.

[28] M. Muttenthaler , G. F. King , D. J. Adams , P. F. Alewood , Nat. Rev. Drug Discovery 2021, 20, 309. 33536635 10.1038/s41573-020-00135-8

[29] A. C.‐L. Lee , J. L. Harris , K. K. Khanna , J.‐H. Hong , Int. J. Mol. Sci. 2019, 20, 2383.31091705

[30] K. Fosgerau , T. Hoffmann , Drug Discov. Today 2015, 20, 122. 25450771 10.1016/j.drudis.2014.10.003

[31] A. Henninot , J. C. Collins , J. M. Nuss , J. Med. Chem. 2018, 61, 1382. 28737935 10.1021/acs.jmedchem.7b00318

[32] R. D. Egleton , T. P. Davis , Peptides 1997, 18, 1431. 9392847 10.1016/s0196-9781(97)00242-8

[33] L. Wang , N. Wang , W. Zhang , X. Cheng , Z. Yan , G. Shao , X. Wang , R. Wang , C. Fu , Signal Transduct. Target. Ther. 2022, 7, 48. 35165272 10.1038/s41392-022-00904-4 PMC8844085

[34] L. Ledesma‐Garcia , J. Thuillier , A. Guzman‐Espinola , I. Ensinck , I. Li de la Sierra‐Gallay , N. Lazar , M. Aumont‐Nicaise , J. Mignolet , P. Soumillion , S. Nessler , Proc. Natl. Acad. Sci. USA 2020, 117, 7745. 32198205 10.1073/pnas.1916085117 PMC7149491

[35] A. Talagas , L. Fontaine , L. Ledesma‐Garca , J. Mignolet , I. Li de la Sierra‐Gallay , N. Lazar , M. Aumont‐Nicaise , M. J. Federle , G. Prehna , P. Hols , PLoS Pathog. 2016, 12, e1005980. 27907189 10.1371/journal.ppat.1005980 PMC5131891

[36] M. B. Neiditch , G. C. Capodagli , G. Prehna , M. J. Federle , Annu. Rev. Genet. 2017, 51, 311. 28876981 10.1146/annurev-genet-120116-023507 PMC6588834

[37] E. Shanker , D. A. Morrison , A. Talagas , S. Nessler , M. J. Federle , G. Prehna , PLoS Pathog. 2016, 12, e1005979. 27907154 10.1371/journal.ppat.1005979 PMC5131902

[38] M. Mahameed , P. Wang , S. Xue , M. Fussenegger , Nat. Commun. 2022, 13, 7350. 36446786 10.1038/s41467-022-35161-0 PMC9708828

[39] T. Suzuki , S. Arai , M. Takeuchi , C. Sakurai , H. Ebana , T. Higashi , H. Hashimoto , K. Hatsuzawa , I. Wada , PLoS One 2012, 7, e37551. 22649538 10.1371/journal.pone.0037551 PMC3359384

[40] J. Yan , Q. Zhang , Z. Guan , Q. Wang , L. Li , F. Ruan , R. Lin , T. Zou , P. Yin , Nat. Plants 2017, 3, 17037. 28394309 10.1038/nplants.2017.37

[41] L. Scheller , T. Strittmatter , D. Fuchs , D. Bojar , M. Fussenegger , Nat. Chem. Biol. 2018, 14, 723. 29686358 10.1038/s41589-018-0046-z

[42] G. A. Gregory , T. I. Robinson , S. E. Linklater , F. Wang , S. Colagiuri , C. de Beaufort , K. C. Donaghue , D. J. Magliano , J. Maniam , T. J. Orchard , Lancet Diabetes Endocrinol. 2022, 10, 741. 36113507 10.1016/S2213-8587(22)00218-2

[43] A. L. Szymczak‐Workman , K. M. Vignali , D. Vignali , Cold Spring Harbor Protocols 2012, 2012, 199. 22301656 10.1101/pdb.ip067876

[44] L. Mátés , M. K. Chuah , E. Belay , B. Jerchow , N. Manoj , A. Acosta‐Sanchez , D. P. Grzela , A. Schmitt , K. Becker , J. Matrai , Nat. Genet. 2009, 41, 753. 19412179 10.1038/ng.343

[45] F. E. Bloom , Annu. Rev. Pharmacol. Toxicol. 1983, 23, 151. 6307119 10.1146/annurev.pa.23.040183.001055

[46] E. Leighton , C. A. Sainsbury , G. C. Jones , Diabetes Ther. 2017, 8, 475. 28484968 10.1007/s13300-017-0265-4 PMC5446389

[47] J. Hu , L. Wang , H. Yang , Y. Meng , M. Tao , Y. Wu , Z. Cao , Peptides 2023, 161, 170927. 36566839 10.1016/j.peptides.2022.170927

[48] X. Zhao , G. Li , S. Liang , J. Anal. Methods Chem. 2013, 2013, 581093. 24490106 10.1155/2013/581093 PMC3893739

[49] T. Saric , C. I. Graef , A. L. Goldberg , J. Biol. Chem. 2004, 279, 46723. 15328361 10.1074/jbc.M406537200

[50] J.‐F. Yao , H. Yang , Y.‐Z. Zhao , M. Xue , Curr. Drug Metab. 2018, 19, 892. 29956618 10.2174/1389200219666180628171531

[51] L. Di , AAPS J. 2015, 17, 134. 25366889 10.1208/s12248-014-9687-3 PMC4287298

[52] J. Chatterjee , F. Rechenmacher , H. Kessler , Angew. Chem., Int. Ed. 2013, 52, 254. 10.1002/anie.201205674 23161799

[53] X. Wei , C. Zhan , X. Chen , J. Hou , C. Xie , W. Lu , Mol. Pharmaceutics 2014, 11, 3261. 10.1021/mp500086e 24673510

[54] R. W. Cheloha , T. Watanabe , T. Dean , S. H. Gellman , T. J. Gardella , ACS Chem. Biol. 2016, 11, 2752. 27533344 10.1021/acschembio.6b00404 PMC5107307

[55] R. Whiley , J. Hardie , Int. J. Syst. Evol. Microbiol. 1988, 38, 335.

[56] J. L. Simonsen , C. Rosada , N. Serakinci , J. Justesen , K. Stenderup , S. I. Rattan , T. G. Jensen , M. Kassem , Nat. Biotechnol. 2002, 20, 592. 12042863 10.1038/nbt0602-592

