# Supplementary material for: Ultrashort‐Peptide‐Responsive Gene Switches for Regulation of Therapeutic Protein Expression in Mammalian Cells
Source: Adv Sci (Weinh). 2024 May 13;11(28):2309411. doi: 10.1002/advs.202309411 (PMC11267282; doi:10.1002/advs.202309411)
Supplement: Supplementary file 1 — Supporting Information [file ADVS-11-2309411-s001.pdf]

ADVANCED SCIENCE
Open Access

Supporting Information

for *Adv. Sci.*, DOI 10.1002/advs.202309411

Ultrashort-Peptide-Responsive Gene Switches for Regulation of Therapeutic Protein Expression in Mammalian Cells

*Jinbo Huang, Shuai Xue, Yu-Qing Xie, Ana Palma Teixeira and Martin Fussenegger**

Supporting Information

Ultrashort-peptide-responsive gene switches for regulation of therapeutic protein expression in mammalian cells

Jinbo Huang[1], Shuai Xue[1,3], Yu-Qing Xie[1], Ana Palma Teixeira[1], Martin Fussenegger[1,2,*]

[1]Department of Biosystems Science and Engineering, ETH Zurich, Klingelbergstrasse 48, CH-4056 Basel, Switzerland.

[2]Faculty of Science, University of Basel, Klingelbergstrasse 48, CH-4056 Basel, Switzerland.

[3]Present address: Key Laboratory of Growth Regulation and Translational Research of Zhejiang Province, School of Life Sciences, Westlake University, Hangzhou, Zhejiang, China.

[*]Corresponding author. E-mail: martin.fussenegger@bsse.ethz.ch.

This file includes:

- Supplementary Figures: 1-19
- Supplementary Tables: 1-4
- Supplementary References

Supplementary Figures

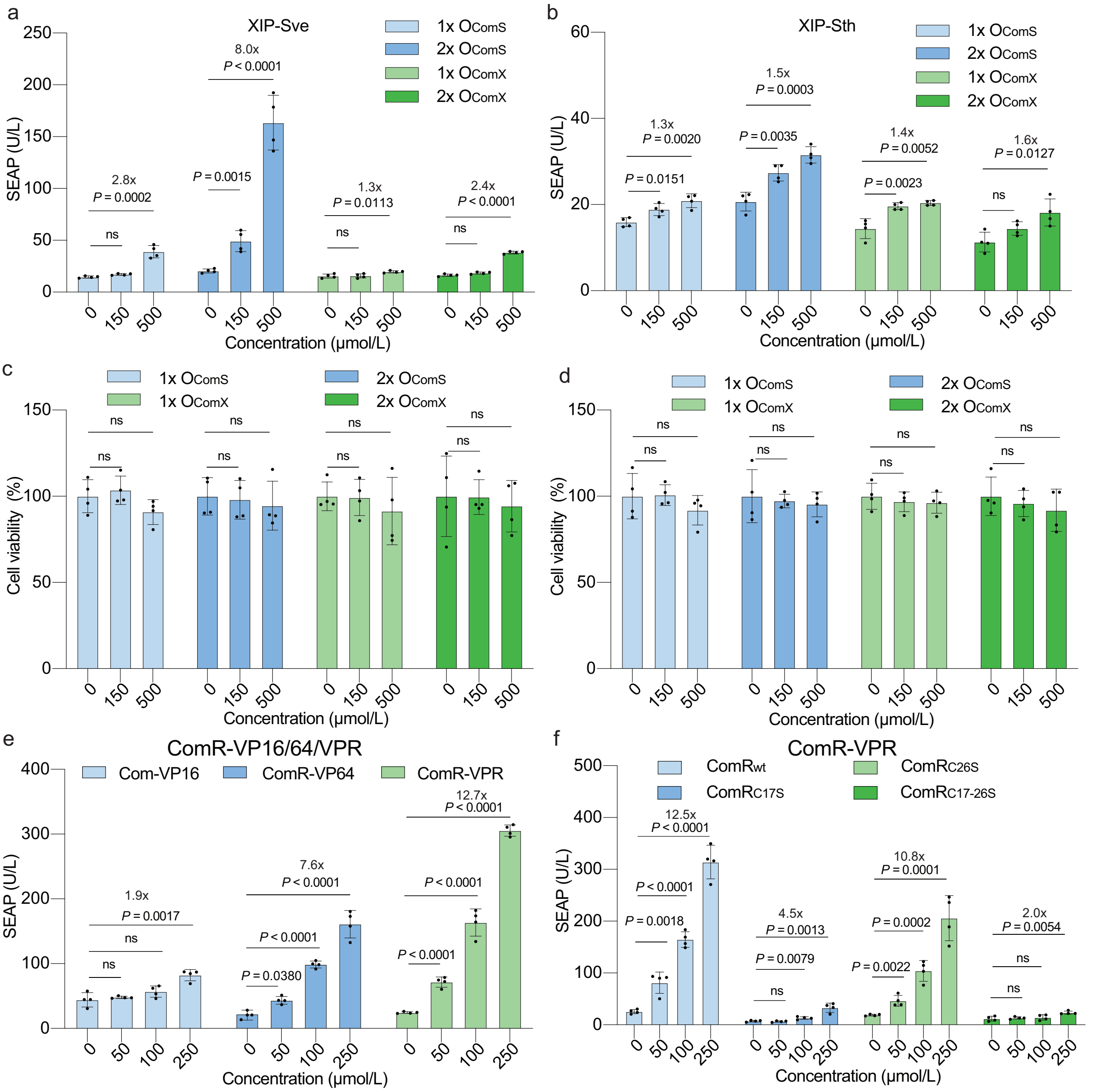

Supplementary Figure 1. Design and optimization of a short-peptide-induced system in engineered HEK-293T cells. a,b, Screening of Com operators with XIP from *Streptococcus vestibularis* (XIP-Sve) (**a**) and XIP from *Streptococcus thermophilus* (XIP-Sth) (**b**). **c**,**d**, Impact of peptide induction on the viability of engineered HEK-293T cells. Cells were exposed to XIP-Sve (**c**) or XIP-Sth (**d**) at the indicated concentrations, and cell viability was measured using resazurin assay. **e**, The ComR protein was fused with transactivation domain variants, VP16, VP64 and VPR. **f**, Screening of cysteine-to-serine mutation (C-to-S) in ComR. Two cysteine residues were replaced with serine at the N-terminal of ComR (C17S, C26S and C17-26S). The ComRs fused with the indicated transactivation domain were co-transfected with pJH1543 (O_{Coms9}-$P_{hCMVmin}$-SEAP-pA) in HEK-293 cells to test their performance. O_{ComS} and O_{ComX} are

operator variants of the ComR box from **Fig. 1c**. The sequences of XIP-Sve and XIP-Sth are shown in **Fig. 1d**. SEAP levels in the culture supernatants were measured at 24 h after induction. Data points represent mean ± SD; n = 4. ns means not significant (*P* value > 0.05). The *P* value indicates the significance of differences in the mean values versus the non-induced group.

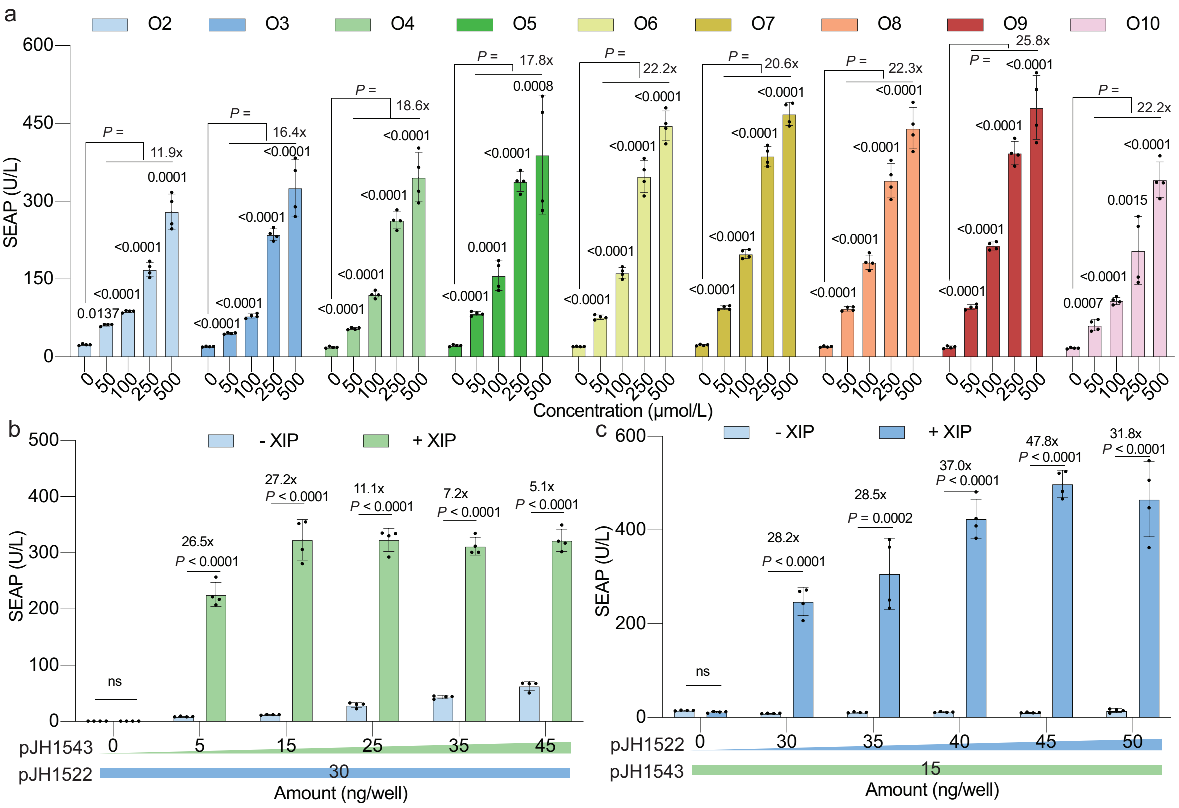

Supplementary Figure 2. Design and characterization of the short-peptide-induced system in engineered HEK-293T cells. a, Performance comparison of engineered cells containing ComS operator variants having two to ten repeats. **b**, **c**, Effect of molar ratio of pJH1543 (O_{Coms9}-$P_{hCMVmin}$-SEAP-pA) and pJH1522 (P_{hCMV}-ComR-VPR-pA). The short-peptide-induced system was transiently transfected with a constant amount of pJH1522 and the indicated amount of pJH1543 (**b**), or a constant amount of pJH1543 and the indicated amount of pJH1522 (**c**) per well of a 96-well plate cell culture. The engineered HEK-293T cells were induced by XIP-Sve at the indicated concentration. VP16, *herpes simplex* virus early transcriptional activator. VP64, four tandem repeats of the *herpes simplex* virus early transcriptional activator VP16. VPR, strong tripartite mammalian transactivator VP64-p65-Rta. WT, wild type. C17S, cysteine residue no. 17 mutated to serine. Data points represent mean ± SD; n = 4. ns means not significant (*P* > 0.05). The *P* value indicates the significance of differences in the mean values versus the non-induced group.

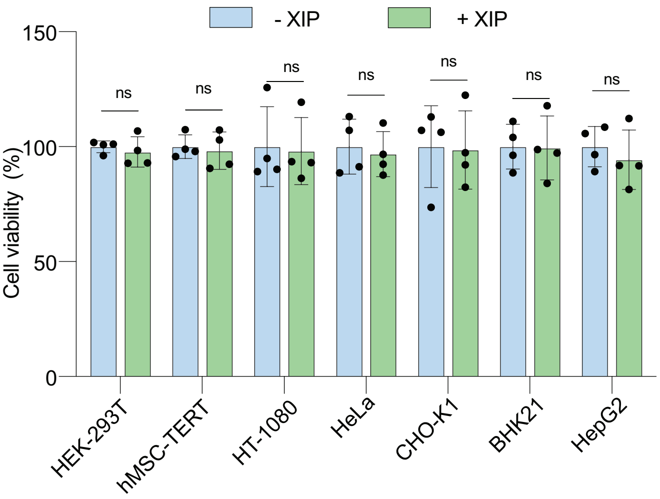

Supplementary Figure 3. Effect of XIP on the viability of mammalian cells. Viability was measured using resazurin assay. All treatment groups were induced with 250 µM XIP (+), and the non-treated groups received an equivalent amount of vehicle (DMSO) (-). This figure is corresponding to **Figure 1g**. Data points represent mean ± SD; n = 4. ns means not significant ($P > 0.05$). The P value indicates the significance of differences in the mean values versus the indicated group.

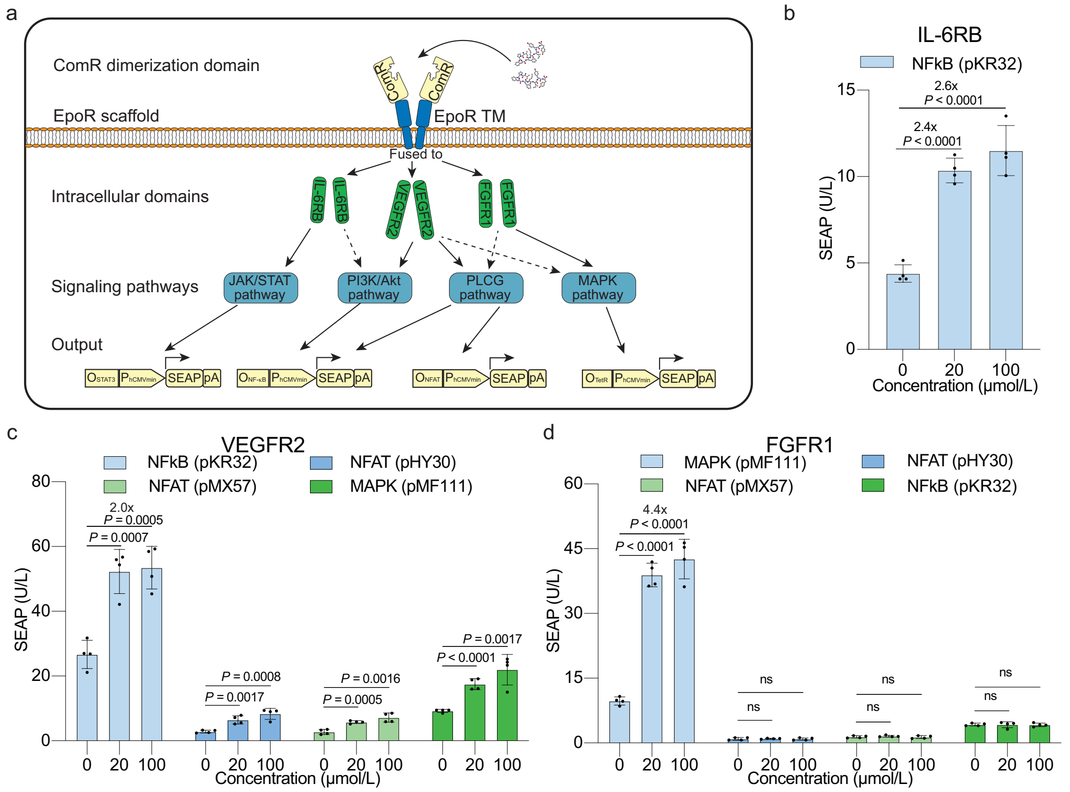

Supplementary Figure 4. Design and primary testing for ComR$_{\text{EXTRA}}$-based short-peptide-induced mammalian system. a, Schematic representation of the ComR$_{\text{EXTRA}}$-based synthetic gene circuit for short-peptide-sensing in mammalian cells. The ComR$_{\text{EXTRA}}$-based

system was composed of an input dimerization domain, which is ComR in our system, fused with the EpoR scaffold and intracellular transduction domains for activating downstream signal pathways and output reporters. The dimerization of ComR is induced by the short peptide in the ON state. **b**, IL-6RB signaling pathway testing with NFκB reporter. The engineered HEK-293 cells were transfected with pJH1567 (P_{SV40}-SP-ComR-EpoR-IL-6RB-pA) and pKR32 ($O_{NF\text{-}\kappa B}$-$P_{hCMVmin}$-SEAP-pA). **c**, VEGFR2 signaling pathway testing. The engineered HEK-293 cells were transfected with pJH1568 (P_{SV40}-SP-ComR-EpoR-VEGFR2-pA) and the indicated reporters. **d**, FGFR1 signal pathway testing. The engineered HEK-293 cells were transfected with pJH1569 (P_{SV40}-SP-ComR-EpoR-FGFR1-pA) and the indicated reporters. Reporter plasmids for the indicated signaling pathway: NFkB, pKR32 ($O_{NF\text{-}\kappa B}$-$P_{hCMVmin}$-SEAP-pA); NFAT, pHY30 (O_{NFAT}-$P_{hCMVmin}$-SEAP-pA) and pMX57 (P_{NFAT3}-SEAP-pA; P_{NFAT3}, $(NFAT_{IL4})_5$-$P_{hCMVmin}$); MAPK, pMF111 (O_{TetR}-$P_{hCMVmin}$-SEAP-pA) co-transfected with Mkp37 (P_{hCMV}-TetR-ELK1-pA). EpoR TM, erythropoietin receptor transmembrane domain. IL-6RB, interleukin 6 receptor B. VEGFR2, vascular endothelial growth factor receptor 2. FGFR1, fibroblast growth factor receptor 1. JAK/STAT, Janus kinase/signal transducer and activator of transcription. PI3K/Akt, phosphatidylinositol 3-kinase/protein kinase B. PLCG, phospholipase C gamma. MAPK, mitogen-activated protein kinase; induced by FGFR1. NFkB, nuclear factor 'kappa-light-chain-enhancer' of activated B-cells. NFAT, nuclear factor of activated T-cells. TetR-ELK1, a synthetic transcription factor containing a Tet operator binding domain (TetR) and a transactivation domain (ELK1). The solid arrows indicate well-documented signal pathways, while the dashed arrows indicate possible signal pathways. Data points represent mean ± SD; n = 4. ns means not significant ($P > 0.05$). The P value indicates the significance of differences in the mean values versus the non-induced group.

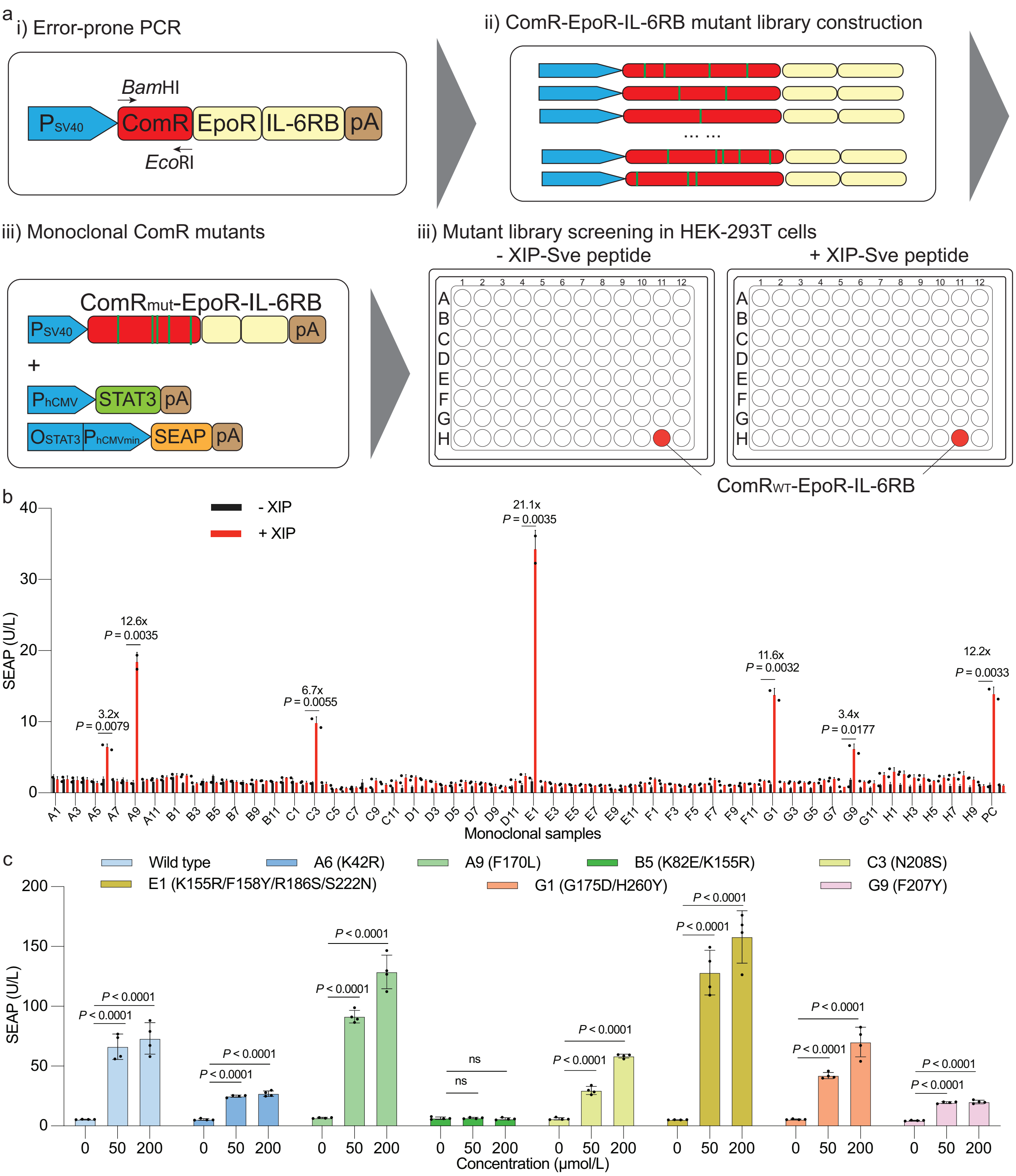

Supplementary Figure 5. Random mutagenesis of ComR and high-throughput assessment of mutant variants. a. Schematic model of error-prone PCR work flow to evaluate the mutants. The structural organization of pJH1567 plasmid (P_{SV40}-SP-ComR-EpoR-IL-6RB-pA) is shown. The region highlighted in red is ComR to be amplified by error-prone PCR. The PCR products were recovered and ligated with the template vector digested with *Bam*HI and *Eco*RI by Gibson assembly. The ligated plasmids were amplified in competent cells overnight and inoculated monoclonally into a 96-well plate. The monoclonal mutants were co-transfected with pJH1577 (O_{STAT3}-$P_{hCMVmin}$-SEAP-pA) and pJH1578 (P_{hCMV}-STAT3-pA) into HEK-293 cells seeded in

96-well culture plates. Each mutant was transfected in the same position of four plates, of which two were used as blank controls, and the other two were induced by XIP-Sve peptide (50 μM). **b**, Primary screening of SP-ComR-EpoR-IL-6RB plasmid library. SEAP levels were analyzed in non-induced (black) and induced (50 μM XIP-Sve peptide, red) groups. The variants with good performance in terms of fold changes were selected for next round evaluation. PC, positive control with wild-type ComR. **c**. Further confirmation of ComR variants. The selected variants were confirmed by sequencing. HEK-293 cells were transfected with 10 ng/well of pJH1577 and 30 ng/well pJH1578 plasmids and XIP-Sve peptide was added to the culture at the indicated concentration. **c**, Evaluation of the top six clones (Mut-A6, A9, C3, E1, G1, G9) obtained from initial screening (**b**). The non-inducible clone Mut-B5 was selected as a negative control. The engineered cells were induced with XIP at the indicated concentrations. SEAP levels were quantified in the culture supernatant after induction for 24 h. Data points represent mean ± SD, n = 2 in (**b**), and n = 4 in (**c**). ns means not significant ($P > 0.05$). The P value indicates the significance of differences in the mean values versus the non-induced group.

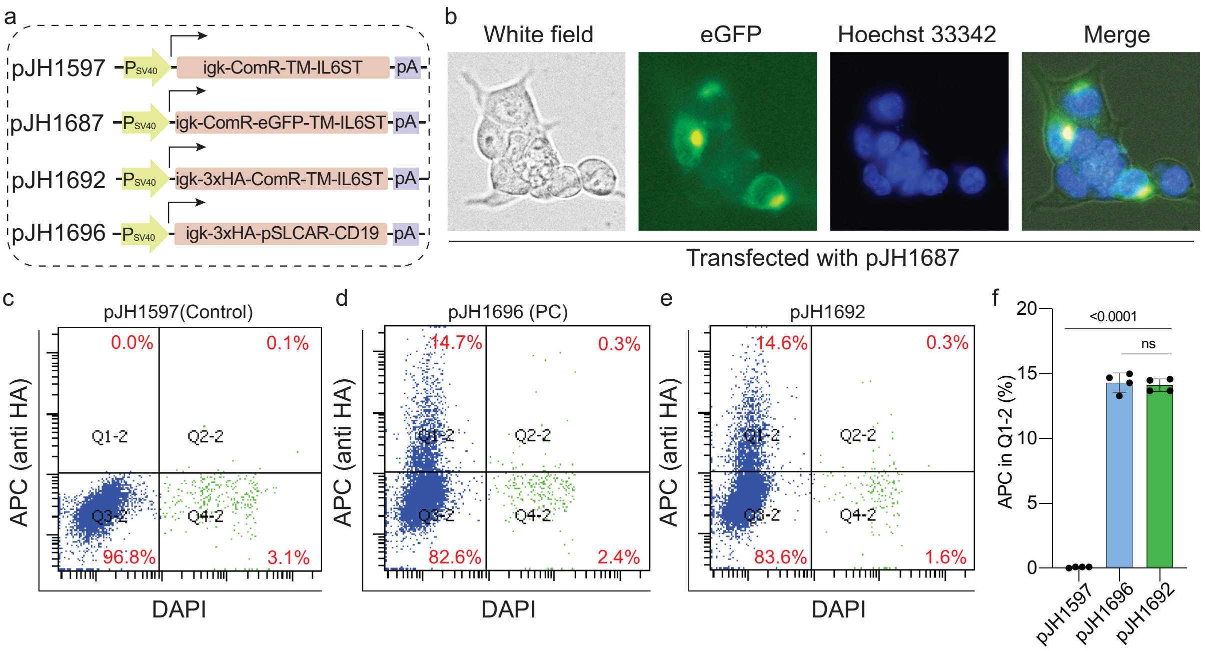

Supplementary Figure 6. Verification of extracellular presentation of ComR in the ComR$_{EXTRA}$-based system. a, Design of plasmids to transfect cells for fluorescence-microscopic imaging and flow-cytometric analysis. **b**, Fluorescence-microscopic imaging of HEK-293T cells transfected with pJH1687. HEK-293T cells were transfected with pJH1687 and cultured at 37 °C for 36 hours. Fluorescence imaging of eGFP (488/510) was performed using a Nikon wide-field microscopic platform. Hoechst 33342 was utilized for nuclear localization. **c-e**, Flow-cytometric analysis of HEK-293T cells transfected with pJH1597 (c),

pJH1696 (d), and pJH1692 (e). Following transfection with the respective plasmids, HEK-293T cells were collected, washed, and incubated with APC-labeled anti-3xHA antibody for 20 minutes at 4 °C. The cells were then washed with PBS and stained with DAPI before flow cytometry. The APC (630/660) and DAPI (360/460) channels were utilized for cell analysis. The Q1-2 gate indicates the APC signal. **f**, Bar plot for the Q1-2 gate among negative control (pJH1597), positive control (pJH1696), and targeting sample (pJH1692). Data points represent mean ± SD; n = 4. ns means not significant ($P > 0.05$). The P value indicates the significance of differences between the mean values in the indicated groups.

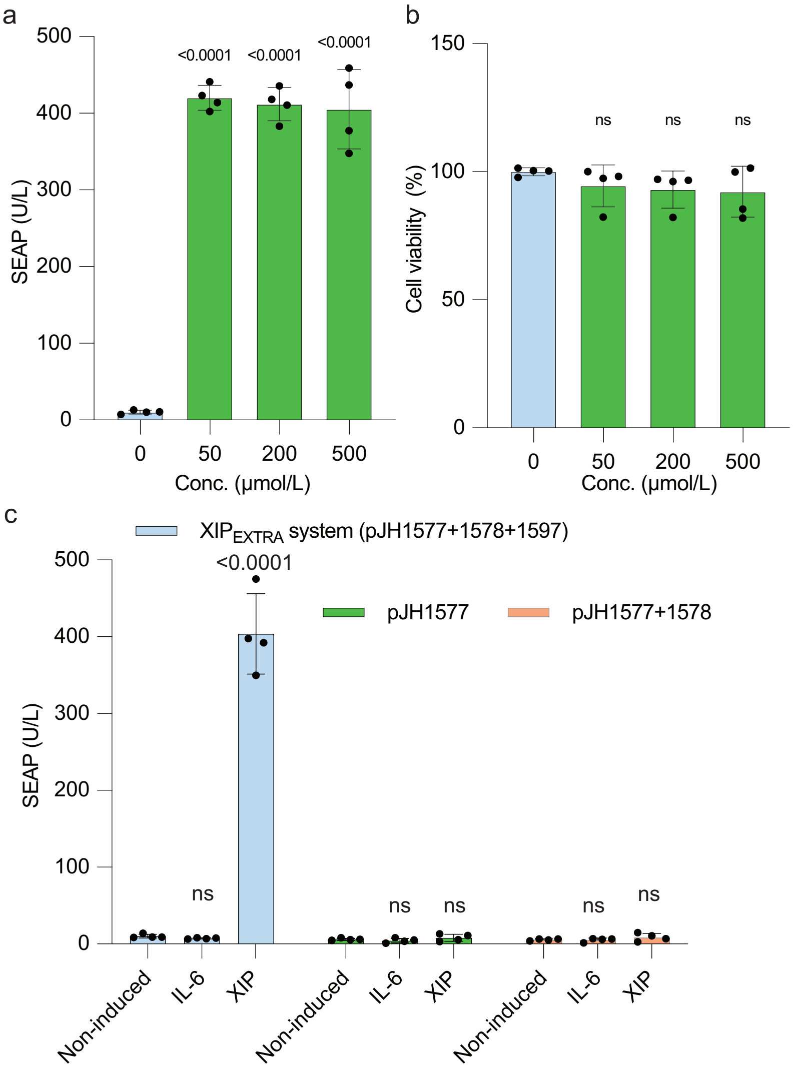

Supplementary Figure 7. Effect of overstimulation with XIP and IL-6 on mammalian cell lines expressing the ComR$_{EXTRA}$-based system. a,**b**, SEAP profile (**a**) and cell viability assessment (**b**) after 24 h of incubation with XIP peptide at the indicated concentrations. Viability was determined using resazurin assay. The non-treated groups received an equivalent amount of vehicle (DMSO) (0). **c**, SEAP profiling in HEK-293T cells transfected with the

ComR$_{EXTRA}$-related plasmids after incubation with IL-6 or XIP peptide. The concentration of IL-6 was 50 ng/mL, and the concentration of XIP was 50 μmol/L. Data points represent mean ± SD; n = 4. ns means not significant (P > 0.05). The P value indicates the significance of differences in the mean values versus the corresponding non-induced group.

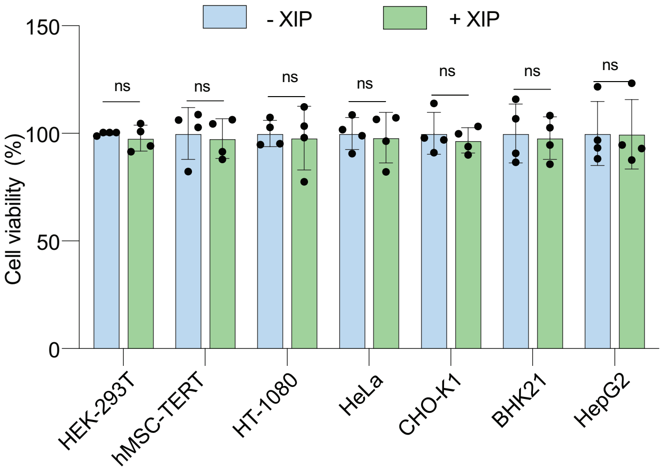

Supplementary Figure 8. Effect of XIP on the viability of various mammalian cell lines expressing the ComR$_{EXTRA}$-based system. Viability was measured using resazurin assay. All treatment groups were induced with 250 μM XIP (+), and the non-treated groups received an equivalent amount of vehicle (DMSO) (-). This figure is corresponding to **Figure 3d**. Data points represent mean ± SD; n = 4. ns means not significant (P > 0.05). The P value indicates the significance of differences in the mean values versus the indicated group.

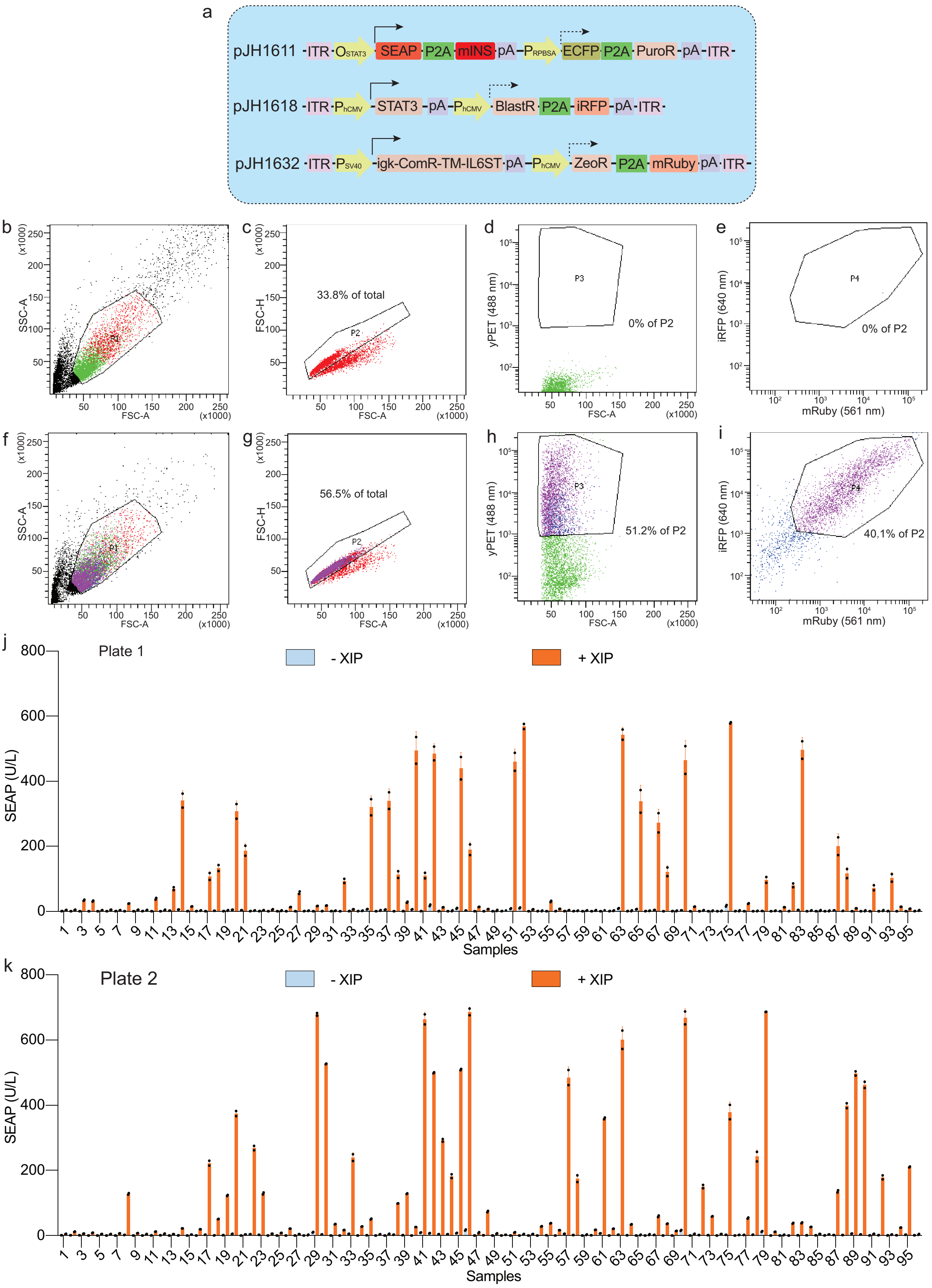

Supplementary Figure 9. Generation and characterization of monoclonal cell line. a, Design of plasmids used for the generation of monoclonal cell lines. The stable cell line constitutively expresses STAT3 (ITR-P_{hCMV}-STAT3-pA:P_{RPBSA}-ECFP-P2A-PuroR-pA-ITR,

pJH1618), Engineered ComR (ITR-P_{SV40}-SP-$ComR_{K155R/F158Y/R186S/S222N/C17S}$-EpoR-IL-6RB-pA: P_{hCMV}-ZeoR-P2A-mRuby-pA-ITR, pJH1632) and tandem STAT3 operator-controlled SEAP followed by mouse insulin (mINS) (ITR-O_{STAT3}-SEAP-P2A-mINS-pA:P_{RPBSA}-ECFP-P2A-PuroR-pA-ITR, pJH1611). All the constructs contain flanking inverted terminal repeats (ITR) for the recognition of SB100X transposase. **b**-**i**, Gating strategies of fluorescence-activated cell sorting (FACS) analysis for monoclonal cells lines using yPET (517/530), mRuby (558/605) and iRFP (690/713). **b-e**, human HEK-293T cells without transfection were used as a negative control. **f**-**i**, Human HEK-293T cells cotransfected with pJH1611, pJH1618, pJH1632 and SB100X transposase were sorted for selection of genomic integration of the XIP-induced system using yPET (517/530), mRuby (558/605) and iRFP (690/713). Triple-positive monoclonal cells were selected for two-week culture in medium containing 0.5 µg/ml puromycin, 5 µg/ml blasticidin and 100 µg/ml of zeocin for antibiotic selection. **j**,**k**, Screening of monoclonal HEK-293T cell lines on plate 1 (**j**) and plate 2 (**k**). HEK-293T cells were stably transfected with pJH1611, pJH1618, pJH1632 and SB100X transposase. SEAP production was profiled from two 96-well plates of monoclonal cell lines after induction with XIP (25 µM) for 24 h. 10,000 cells were analyzed in **b-e** and **f-i**, respectively. In **j**,**k**, data points represent mean ± SD; n = 2.

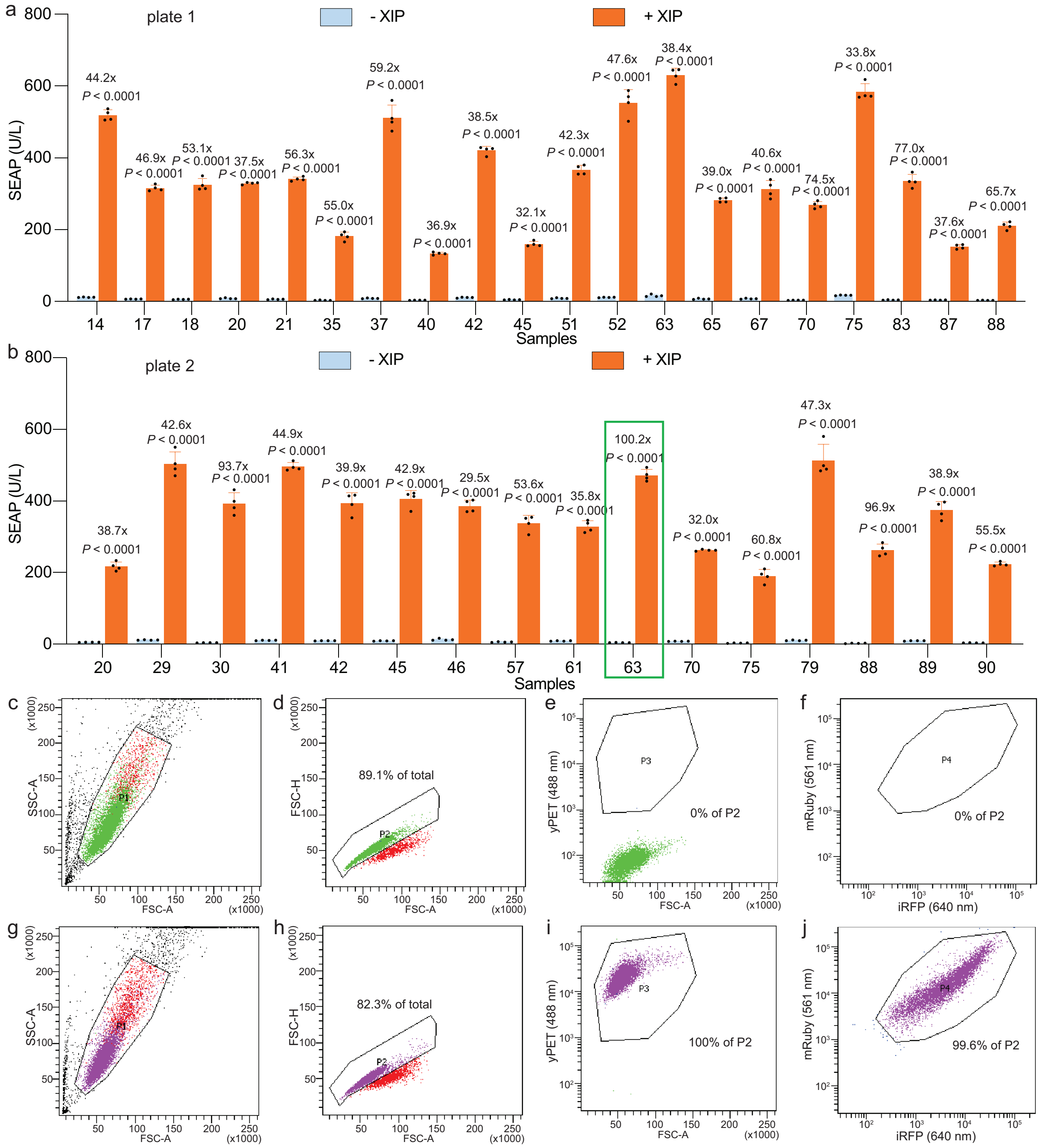

Supplementary Figure 10. Secondary screening and confirmation of monoclonal cell line. a,b, Evaluation of the top clones obtained from plate 1 and plate 2 in **Supplementary Fig. 9**. SEAP levels were quantified in the culture supernatant after induction for 24 h. The most promising monoclonal cell line, designated HEK-XIP and highlighted in green, was selected for follow-up experiments. **c-j**, Flow cytometric analysis of HEK-XIP monoclonal cells using yPET (517/530), mRuby (558/605) and iRFP (690/713). **c-f**, Gating strategy implemented for human HEK-293T cells without transfection, used as a negative control. **g-j**, Fluorescence analysis of HEK-XIP monoclonal cells using yPET (517/530), mRuby (558/605) and iRFP (690/713). The triple-positive population reached 99.6%, confirming that HEK-XIP is indeed

a monoclonal cell line. All data are presented as means ± SD; in **a** and **b**, n = 4; for all groups in **a** and **b**, *P* values < 0.0001. The *P* values were calculated for the differences between induced and non-induced groups. 10,000 cells were analysed in **c-f** and **g-j**, respectively.

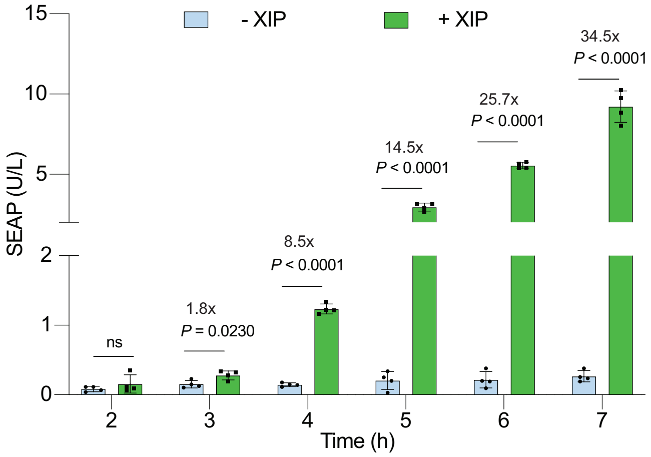

Supplementary Figure 11. Expression kinetics of HEK-XIP cells up to 7 hours. SEAP produced during the first 7 h by HEK-XIP cells non-induced or induced with 20 µM of XIP. The induction factors were calculated between the indicated groups. All data are presented as means ± SD; n = 4. The *P* values were calculated for the differences between induced and non-induced groups.

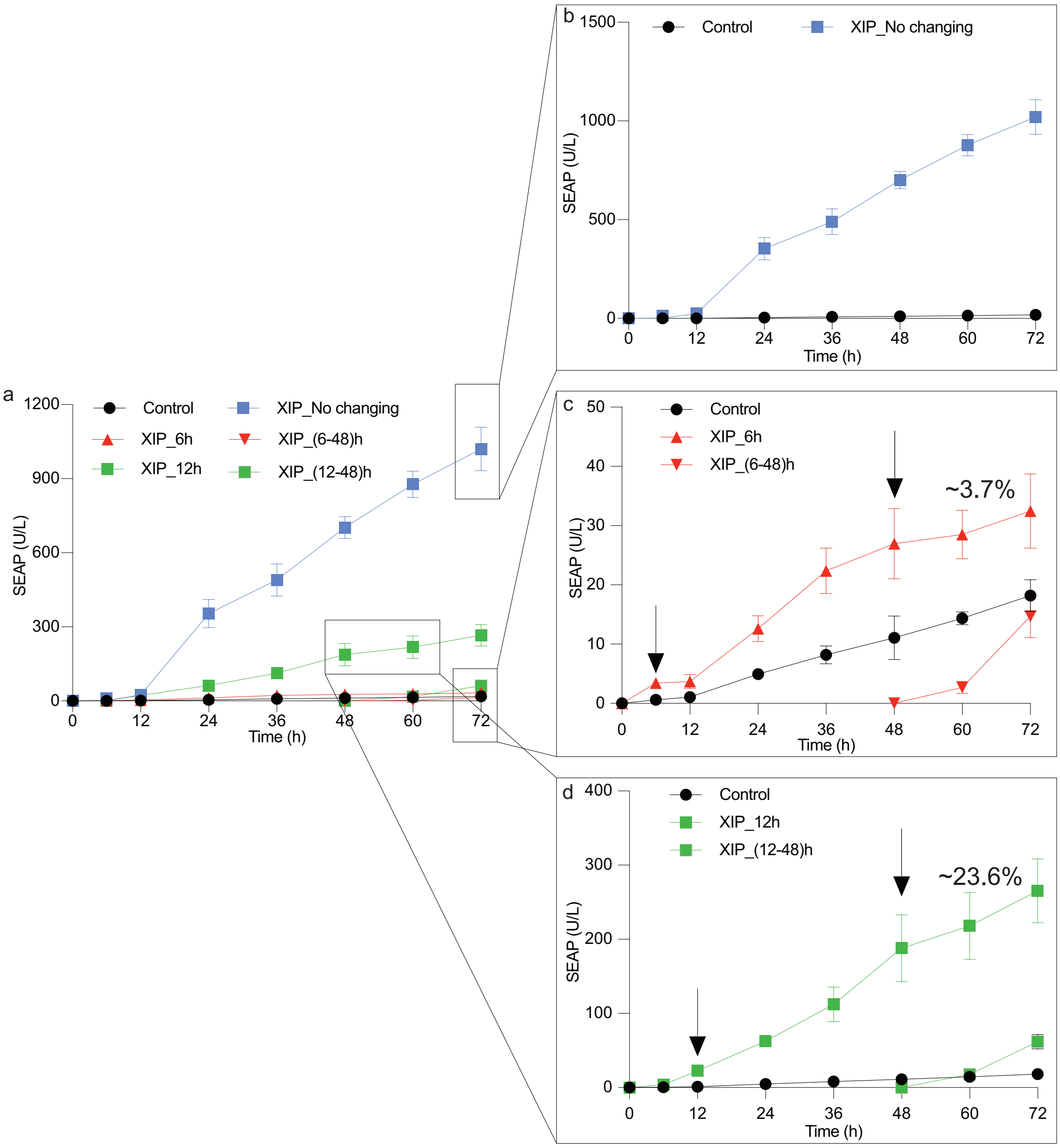

Supplementary Figure 12. SEAP production kinetics were monitored over a 72-h period in HEK-XIP cells. a-d, In all treatment groups (but not the "Control"), cells were initially exposed to 50 µM XIP peptide at time point 0. Following induction, the "XIP_No changing" group was kept in the same medium throughout the 72-hour period, while in the "XIP_6h", "XIP_(6-48)h", "XIP_12h", and "XIP_(12-48)h" groups, the medium was replaced with XIP-free medium at the specified time points. **b-d**, Zoomed-in graphs were generated from the data in (**a**), with (**b**) representing the "XIP_No changing" group, (**c**) depicting the "XIP_6h" and "XIP_(6-48)h" groups, and (**d**) illustrating the "XIP_12h" and "XIP_(12-48)h" groups. Note that compared to the "XIP_No changing" group, HEK-XIP cells in the "XIP_6h" and "XIP_12h" groups retain approximately 3.7% and 23.6% expression efficiency at 72 h, respectively, despite the replacement of the medium with XIP-free medium after 6 h and 12 h of incubation, respectively. All data are presented as means ± SD; n = 4.

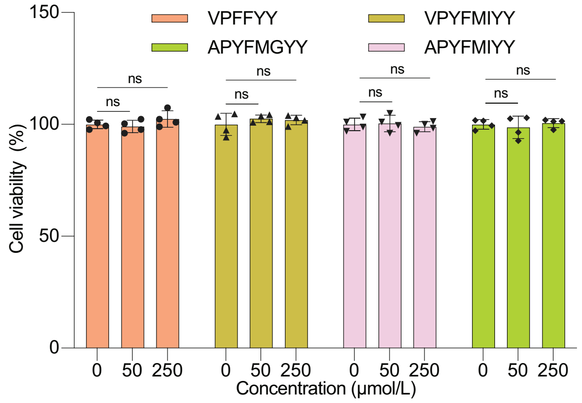

Supplementary Figure 13. Effect of various short peptides on the viability of HEK-XIP monoclonal cells. Viability was measured using resazurin assay after 24 h treatment with the indicated concentrations of peptides. Data points represent mean ± SD; n = 4. ns means not significant ($P > 0.05$). The P value indicates the significance of differences in the mean values versus the indicated group.

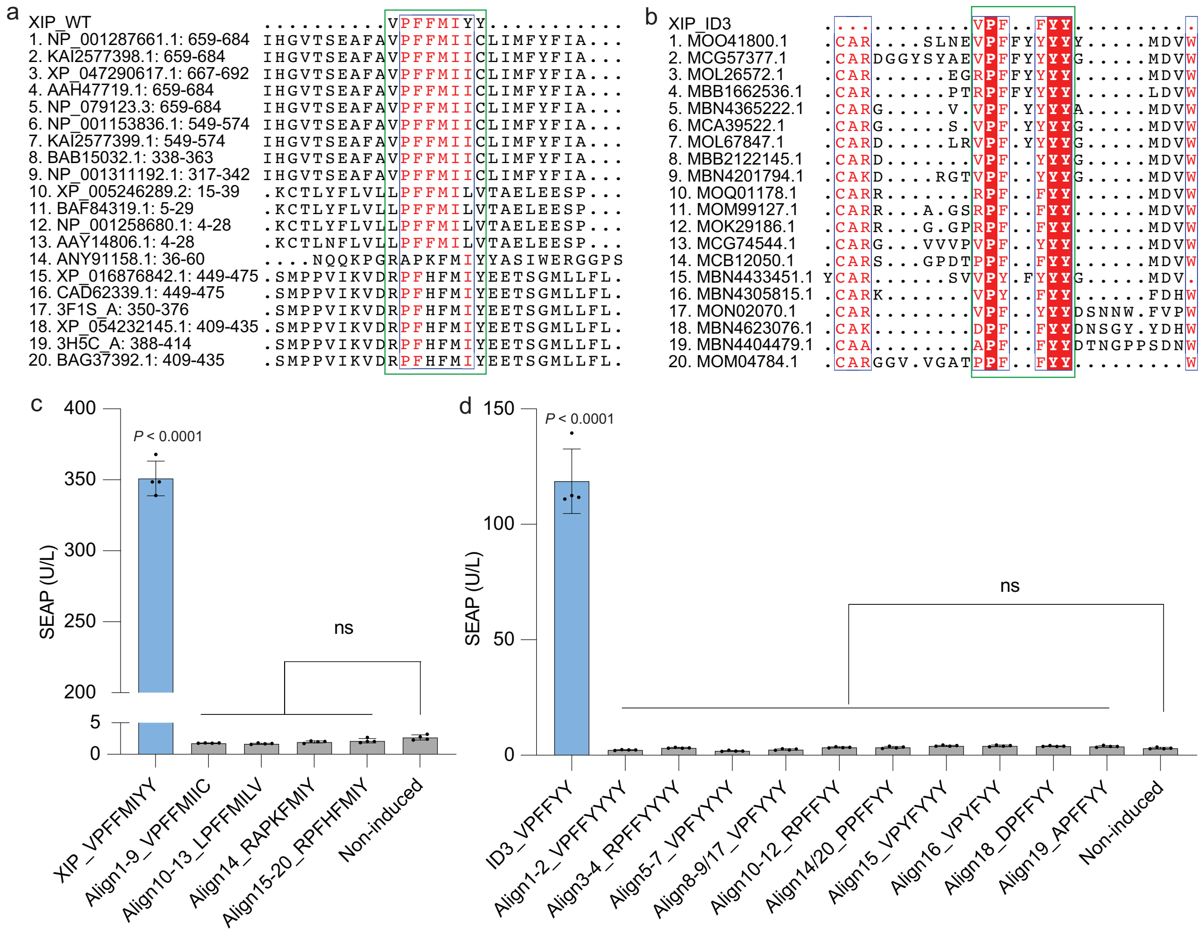

Supplementary Figure 14. Sequence BLAST based on XIP_WT and XIP_ID3 peptides using the NCBI database. a,b, Sequence alignment for the top 20 best-matched results from

the NCBI database using XIP_WT (VPFFMIYY) (**a**) and XIP_ID3 (VPFFYY) (**b**). The most conserved regions are highlighted with green rectangles. The sequencing alignments were performed on Multalin platform (http://multalin.toulouse.inra.fr/multalin/). **c,d**, HEK-XIP monoclonal cells were induced using the indicated peptides based on sequence alignment results from the most conserved regions (highlighted) of **a** and **b**. SEAP levels were quantified in the culture supernatant after 24 h of induction. NCBI, National Center for Biotechnology Information, https://www.ncbi.nlm.nih.gov/. All data are presented as means ± SD; n = 4. ns means not significant (*P* >0.05). The *P* values were calculated for the differences between induced and non-induced groups.

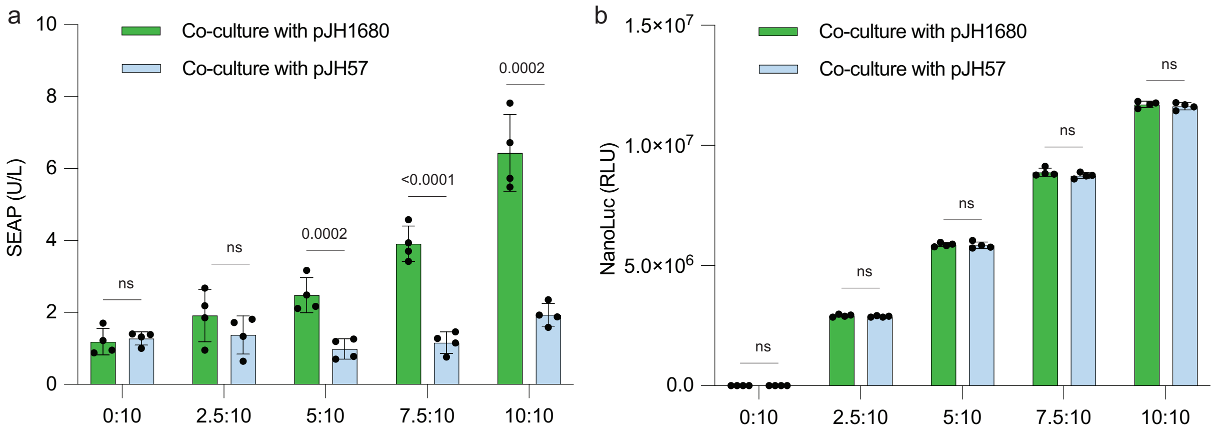

Supplementary Figure 15. Co-cultivation of HEK-XIP cells with HEK-293T cells transfected with pJH1680. **a-b**, HEK-293T cells (sender cells) were initially seeded in 10-cm dishes and transfected with pJH1680 ($P_{EF\text{-}1\alpha}$-NanoLuc-GS1-XIP-pA) for 24 h. Subsequently, these cells were counted and co-cultured with HEK-XIP cells (receiver cells) in 96-well plates at the specified ratio. The number of HEK-293T cells transfected with pJH1680 in the co-culture was progressively increased, with 15,000 HEK-XIP cells present in each well. For comparative purposes, HEK-293T cells were also transfected with pJH57 (P_{hCMV}-NanoLuc-pA) and co-cultured with HEK-XIP cells. The levels of SEAP (**a**) and NanoLuc (**b**) were quantified in the culture supernatant following co-cultivation for 24 h. All data are presented as means ± SD; n = 4. ns means not significant (*P* >0.05). The *P* values were calculated for the differences between induced and indicated groups.

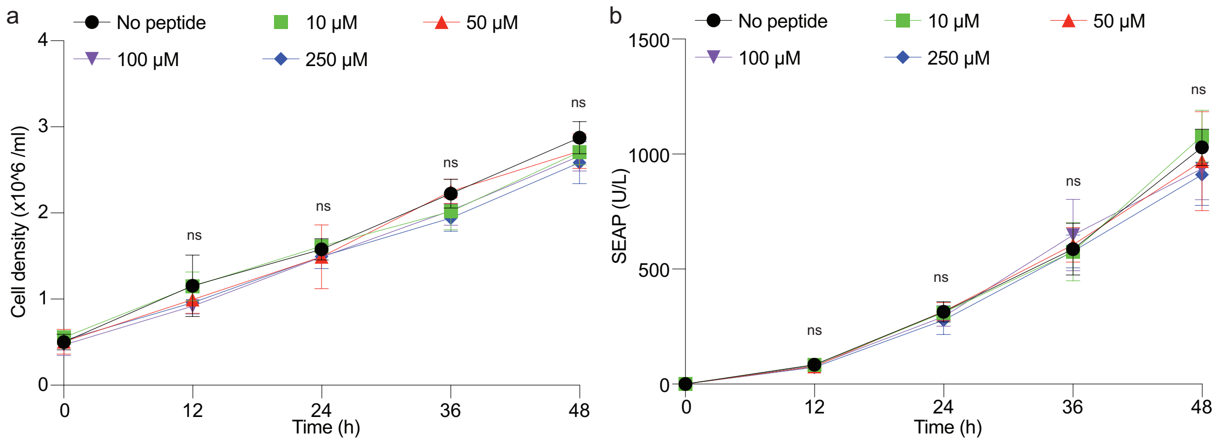

Supplementary Figure 16. Assessment of cell growth and recombinant protein production in the presence of XIP. a,**b**, HEK-293 cells were seeded 24 h before incubation with the indicated concentration of the XIP peptides. Cell density (**a**) and recombinant SEAP production (**b**) were analyzed every 12 h during 48 h of culture. HEK-293 cells with constitutive expression of SEAP in (**b**) were transfected with pJH3 (P_{hCMV}-SEAP-pA). All the treatment groups shown no significant difference compared with the non-induced group. Data points represent mean ± SD, n = 4. ns means not significant (*P* >0.05) (versus non-induced control).

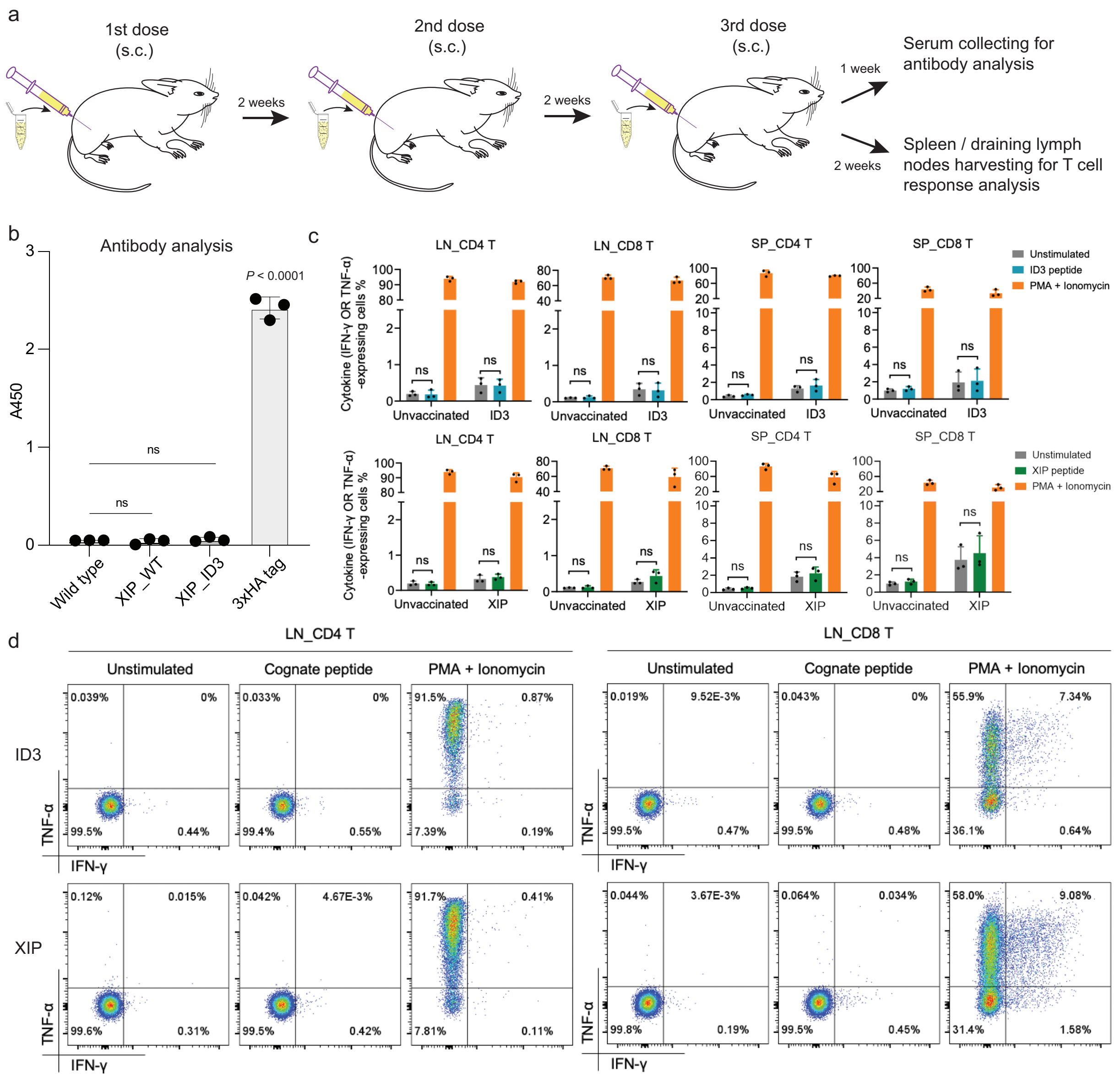

Supplementary Figure 17. Immunogenicity assessment for short peptides in mice. a, Procedures for C57BL/6 mice immunization. Mice were immunized with peptides three times at 2-week intervals. Blood samples were taken at one week after the final booster for ELISA testing. Spleen and draining lymph nodes were taken within two weeks of the final booster for flow cytometry testing. **b**, Antibody analysis in blood samples from animals immunized with the indicated peptides. **c**, Lymphocytes isolated from spleen or lymph nodes were *ex vivo*-stimulated with cognate peptides for 4 hours. Frequencies of cytokine producing cells (IFN-gamma+ or TNF-alpha+) among either CD4 T cells or CD8 T cells after the stimulation were quantified by FACS. Cells were stimulated with PMA + Ionomycin as internal positive controls. **d**, Representative flow cytometry plots showing the frequencies of cytokine-producing cells among either CD4 T cells or CD8 T cells in draining lymph nodes. s.c., subcutaneous injection; SP, spleen; LN, lymph node; PMA, phorbol myristate acetate. All data are presented as means

± SD; in (**b**), (**c**) and (**d**), n = 3. ns means not significant (*P* >0.05). The *P* values were calculated for the differences between induced and wild-type groups.

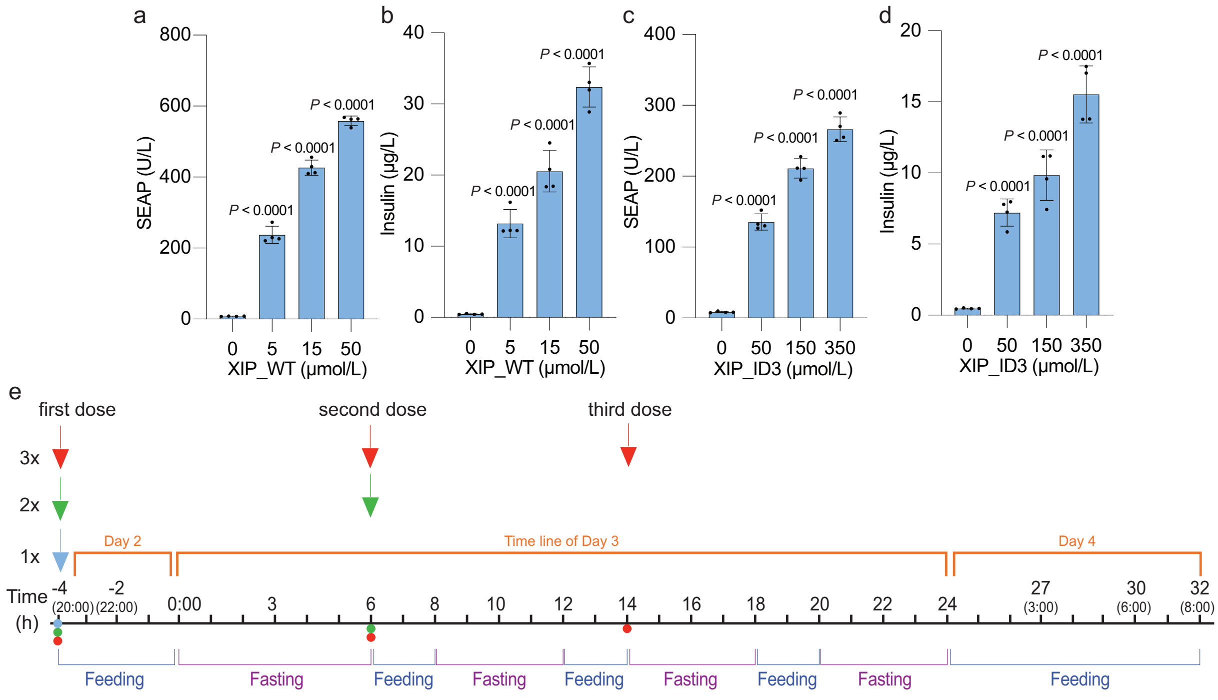

Supplementary Figure 18. SEAP and insulin production by alginate-encapsulated HEK-XIP cells. a,d. The microencapsulated cells were cultured in 24-well plates with DMEM medium containing 10% fetal bovine serum, and induced with XIP_WT (**a** and **b**) and XIP_ID3 (**c** and **d**) at the indicated concentrations. The SEAP (**a** and **c**) and insulin (**b** and **d**) levels in the culture supernatant were quantified after induction for 24 h. **e**, Schedule of induction with different frequencies and the fasting-feeding cycle for all mice throughout the experiment. Columns are each the mean ± SD of the four determinations indicated by dots. The *P* value indicates the significance of differences in the mean values (versus the non-induced group).

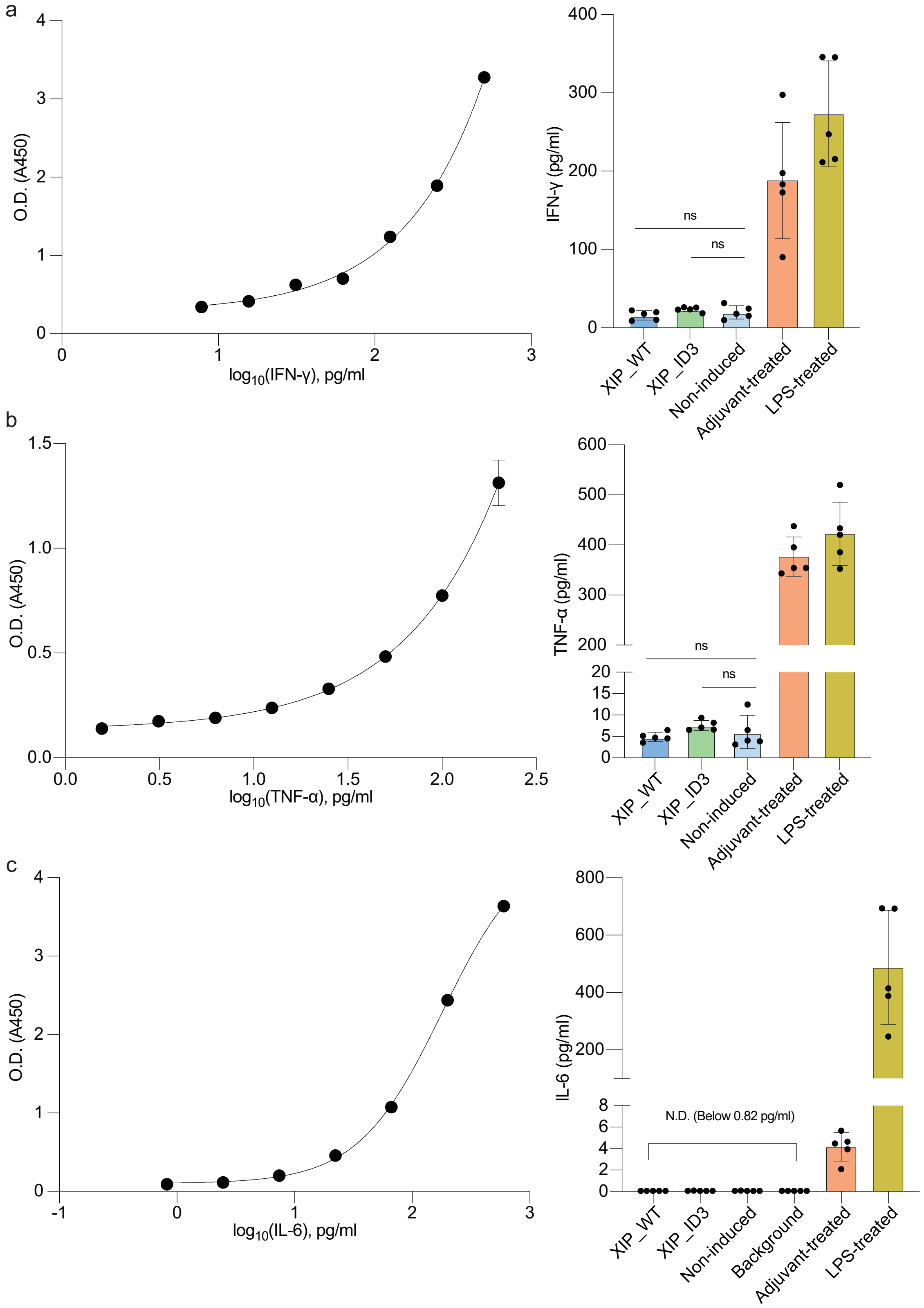

Supplementary Figure 19. Profiling of inflammatory cytokines in serum of T1D mice. a-c, IFN-γ (**a**), TNF-α (**b**) and IL-6 (**c**) levels were measured in the serum of peptide-induced and non-induced mice at four weeks after implantation of alginate-encapsulated monoclonal HEK-XIP cells. The left panels show the calibration curves calculated using a 4-parameter curve fit,

and the right panels in (**a**) and (**b**) show the values acquired for each cytokine in untreated and treated HEK-XIP engineered mice. The right panel in (**c**) shows the absorbance value of the IL-6 ELISA kit for each group, indicating that the IL-6 levels are undetectable in treated and untreated mice (below 0.82 pg/ml). In (**c**), the background is the plate blank without any sample. The adjuvant-treated and LPS-treated mice served as positive controls, with blood samples collected 24 hours post-treatment. In the adjuvant group, each mouse received an intraperitoneal injection of 200 μl of complete Freund's adjuvant/PBS (1:1) buffer, containing 3xHA peptide at a dosage of 7.5 mg/kg. LPS-treated mice received an intraperitoneal injection of 200 μl PBS containing LPS at a dosage of 5 μg/kg. The cytokines were measured using specific ELISA kits. O.D. (A450), optical density absorbance at 450 nm. N.D. means not detectable. Columns are each the mean ± SD of the five determinations indicated by dots. ns means not significant (P >0.05). The P value indicates the significance of differences in the mean values (versus the indicated group).

Supplementary Table 1. Plasmids used and designed in this study

**Plasmid**	**Description**	**Reference**
BB6-BlastR	SB100X-specific transposon containing a constitutive BlastR and iRFP expression unit (ITR-MCS:P_{hCMV}-BlastR-P2A-iRFP-pA-ITR).	Huang et al.[1]
BB6-PuroR	SB100X-specific transposon containing a constitutive ECFP and PuroR expression unit (ITR-MCS:P_{RPBSA}-ECFP-P2A-PuroR-pA-ITR).	Huang et al.[1]
BB6-ZeoR	SB100X-specific transposon containing a constitutive ZeoR and mRuby expression unit (ITR-MCS-pA: P_{hCMV}-ZeoR-P2A-mRuby-pA-ITR).	Huang et al.[1]
H107	Mammalian constitutive expression vector. (P_{hCMV}-EGFP-3FLAG:P_{mPGK}-ZeoR-pA).	ObiO, Shanghai
MKp37	Mammalian TetR-ELK1 fusion protein expression vector (P_{hCMV}-TetR-ELK1-pA).	Keeley et al.[2]
pCDNA3.1(+)	Mammalian constitutive expression vector (P_{hCMV}-MCS-pA).	Life Technologies, CA
pSLCAR-CD19-BBz	Mammalian constitutive expression vector containing CD19-CAR (Addgene No.: 135992). ($P_{EF\text{-}1\alpha\text{-}core}$-pSLCAR-CD19-P2A-EGFP-pA).	ADDGENE [3]

pCK53	CRE-driven SEAP expression vector (P_{CRE}-SEAP-pA).	Kemmer et al.[4]
pCMV-T7-SB100	Mammalian constitutive SB100X expression vector (P_{hCMV}-SB100X-pA) (Addgene no. 34879).	Mates et al.[5, 6]
pdCas9-VPR	Mammalian constitutive dCas9-VPR expression vector (P_{hCMV}- dCas9-VPR-pA) (Addgene no. 63798).	Chavez et al.[7]
pET11a_3xFlag-nsp14	T7-driven 3xFLAG-nsp14 expression vector (P_{T7}-3xFLAG-nsp14-pA) (Addgene no. 169159).	Canal et al. [8]
pLS13	Mammalian reporter plasmid for STAT3-induced SEAP expression (O_{STAT3}-$P_{hCMVmin}$-SEAP-pA).	Schukur et al.[9]
pLS15	Mammalian constitutive STAT3 expression vector (P_{hCMV}-STAT3-pA).	Schukur et al.[9]
pLeo619	Mammalian constitutive $GEMS_{RR120}$ expression vector (P_{SV40}-SP-VHH_{A52}-$EpoR_m$-IL-$6RB_m$-pA).	Scheller et al. [10]
pLeo628	Mammalian constitutive MAPK-$GEMS_{RR120}$ expression vector (P_{SV40}-SP-VHH_{A52}-$EpoR_{m0A}$-$FGFR1_{int}$-pA)	Scheller et al. [10]
pLeo690	Mammalian constitutive VEGFR2-$GEMS_{RR120}$ expression vector without alanines C-terminal of the $EpoR_m$ transmembrane helix (P_{SV40}-SP-VHH_{A52}-$EpoR_{m0A}$-$VEGFR2_{int}$-pA).	Scheller et al. [10]

pLeo1403	Mammalian reporter plasmid for TetR-ELK1 induced NanoLuc expression (O_{TetR}-$P_{hCMVmin}$-NanoLuc-pA).	Maysam et al. [11]
pYL1	$P_{CRE-SRE-NFAT}$–driven SEAP expression vector ($P_{CRE-SRE-NFAT}$-SEAP-pA).	Liu et al. [12]
pMF111	Mammalian reporter plasmid for TetR-ELK1-induced SEAP expression (O_{TetR}-$P_{hCMVmin}$-SEAP-pA)	Fussenegger et al.[13]
pMX57	P_{NFAT3}-driven SEAP expression vector (P_{NFAT3}-SEAP-pA; P_{NFAT3}, $(NFAT_{IL4})_5$-$P_{hCMVmin}$).	Xie et al.[14]
pMX256	SB100X-specific transposon containing a P_{NFAT5}-driven SEAP and mINS expression unit and a constitutive EGFP and ZeoR expression unit (ITR-P_{NFAT5}-SEAP-P2A-mINS-pA:P_{RPBSA}-EGFP-P2A-ZeoR-pAITR).	Xie et al.[14]
pHY30	Mammalian reporter plasmid for NFAT-induced SEAP expression (O_{NFAT}-$P_{hCMVmin}$-SEAP-pA)	Ye et al. [15]
phIR	Mammalian constitutive expression vector containing human insulin receptor (hIR) gene (P_{hCMV}-hIR-pA).	Ye et al. [16]
pKR32	Mammalian reporter plasmid for NF-κB-induced SEAP expression ($O_{NF-\kappa B}$-$P_{hCMVmin}$-SEAP-pA)	Schukur et al.[9]
pAT13	Mammalian constitutive PIP-ELK1 expression vector (P_{hCMV}-PIP-ELK1-pA)	Scheller et al. [10],

pXS101	SB100X-specific transposon containing a constitutive mammalian promotor-driven CREB1-TetR stable expression vector in mammalian cells (ITR-P_{hCMV}-CREB1-TetR-2A-mCherry-2A-PuroR-pA-ITR).	Xue et al.[17]
pJH3	Mammalian constitutive SEAP expression vector (P_{hCMV}-SEAP-pA).	Huang et al.[1]
pJH42	Mammalian constitutive SB100X expression vector (P_{hCMV}-SB100X-pA).	Huang et al.[1]
pJH1005	ARE-driven SEAP expression vector (P_{DART}-SEAP-pA).	Huang et al.[1]
pJH1040	ARE-driven SEAP and mINS expression vector (O_{ARE}-$P_{hCMVmin}$-SEAP-P2A-mINS-pA).	Huang et al.[1]
pJH1043	ARE-driven shGLP-1 and NanoLuc expression vector (O_{ARE}-$P_{hCMVmin}$-shGLP-1-P2A-NanoLuc-pA).	Huang et al.[1]
pJH43	Mammalian constitutive SEAP expression vector (P_{SV40}-SEAP-pA).	Huang et al., unpublished.
pJH44	Mammalian constitutive SEAP expression vector ($P_{EF\text{-}1\alpha}$-SEAP-pA).	Huang et al., unpublished.
pJH45	Mammalian constitutive SEAP expression vector (P_{mPGK}-SEAP-pA).	Huang et al., unpublished.

pJH51	Mammalian constitutive NemR-VPR expression vector.<br>(P_{hCMV}-NemR-VPR-pA).	Huang et al., unpublished.
pJH57	Mammalian constitutive NanoLuc expression vector.<br>(P_{hCMV}-NanoLuc-pA).	Huang et al., unpublished.
pJH1078	Mammalian GBaA-VP16 expression vector (P_{hCMV}-GbaA-VP16-pA).	Huang et al., unpublished.
pJH1079	Mammalian GbaA-VP64 expression vector (P_{hCMV}-GbaA-VP64-pA).	Huang et al., unpublished.
pJH53	Mammalian constitutive NanoLuc and mINS expression vector. (P_{hCMV}-NanoLuc-P2A-mINS-pA).<br>The NanoLuc fragment was PCR-amplified from pLeo1403 with OJH53-I-GF (5’-TCGAAGCGGAATTCACCATGACTAGTGAGACAGACACACTCCT -3’) and OJH53-I-GR (5’-CGTCGCCTGCCTGCTTCAGCAGGGAAAAGTTGGTTGCTCCTGTCTGCGCCAGAATGCGTTCGCAC-3’), and the P2A-mINS fragment was PCR-amplified from pMX256 with OJH53-II-GF (5’-CCTGCTGAAGCAGGCAGGCGACGTGGAGGAGAATCCTGGACCCATGGCCCTGTGGATGCGCTTC-3’) and OJH53-II-GR (5’-TTCTAGACACCGGTGGATCCCTAGTTGCAGTAGTTCTC-3’), then the two fragments were cloned into pJH3 (digested by *Spe*I/*Bam*HI) by Gibson assembly.	This work.
pJH54	Mammalian constitutive NemR-VP16 expression vector. (P_{hCMV}-NemR-VP16-pA).	This work.

	The fragment was PCR-amplified from pJH1078 with OJH54-GF (5'-CAGGAGAAGCAGGCCACTAGTGGTTCCGGAGCCCCCCGACCGATGTCAGC-3') and OJH54-GR (5'- CTTTCTAGACACCGGTGGATCCGCTAGCCTACCCACCGTACTCGTC-3'), and cloned into pJH51 (digested by *Spe*I/*Bam*HI) by Gibson assembly.	
pJH55	Mammalian constitutive NemR-VP64 expression vector. (P_{hCMV}-NemR-VP64-pA).<br>The fragment was PCR-amplified from pJH1079 with OJH55-GF (5'- TCAGGAGAAGCAGGCCACTAGTGGTTCCGGACGGGCTGACGCAT-3') and OJH55-GR (5'- TTTCTAGACACCGGTGGATCCGCTAGCCTATCTAGAGTTAATCAGC-3'), and cloned into pJH51 (digested by *Spe*I/*Bam*HI) by Gibson assembly.	This work.
pJH1504	Mammalian constitutive ComR-VP64 expression vector. (P_{hCMV}-ComR-VP64-pA).<br>The fragment was PCR-amplified from synthesized ComR template (TWIST Bioscience) with OJH1504-GF (5'-AAGCTGTTCGAAGCGGAATTCACCATGTCTATCAAGGACTCCATCGGACTGAG-3') and OJH1504-GR (5'-AGCCCGTCCGGAACCACTAGTCAGGCCATCAGCGGCCTTC-3'), and cloned into pJH55 (digested by *Eco*RI/*Spe*I) by Gibson assembly.	This work.
pJH1522	Mammalian constitutive ComR-VPR expression vector. (P_{hCMV}-ComR-VPR-pA).<br>The fragment was PCR-amplified from pJH1504 with OJH1504-GF (5'-AAGCTGTTCGAAGCGGAATTCACCATGTCTATCAAGGACTCCATCGGACTGAG-3') and OJH1504-GR (5'-AGCCCGTC	This work.

	CGGAACCACTAGTCAGGCCATCAGCGGCCTTC-3’), and cloned into pJH51 (digested by *Eco*RI/*Spe*I) by Gibson assembly.	
pJH1529	O_{comS1}-driven SEAP expression vector. (O_{comS1}-$P_{hCMVmin}$-SEAP-pA).<br>The fragment was PCR-amplified from pJH1005 with OJH1529-GF (5’-GGATCGGGAGATCTCCAC GCGTATTAGCTACTAGTGACATTTATGTCACTAACACTAACTCGAGGGTAGGCGTGTACG -3’) and SEAP-BB3-GR (5’-AGCTTTCTAGACACCGGTGGATCCCTAGGTCTGCTCGAATCTG CCGG-3’), and cloned into pJH1005 (digested by *Mlu*I/*Bam*HI) by Gibson assembly.	This work.
pJH1530	O_{comS2}-driven SEAP expression vector. (O_{comS2}-$P_{hCMVmin}$-SEAP-pA).<br>The fragment was firstly PCR-amplified from pJH1005 with OJH1530-GF1 (5’-GTCACTAACACTA AATTAGCTACTAGTGACATTTATGTCACTAACACTAACTCGAGGGTAGGCGTGTACG-3’) and SEAP-BB3-GR (5’-AGCTTTCTAGACACCGGTGGATCCCTAGGTCTGCTCGAATCTGCCG G-3’), then the PCR products were taken as the template in the second round of PCR-amplification using OJH1530-GF2 (5’-GGATCGGGAGATCTCCACGCGTATTAGCTACTAGTGACATTTATG TCACTAACACTAAATTAGCTACTAGTGAC-3’) and SEAP-BB3-GR (5’-AGCTTTCTAGACAC CGGTGGATCCCTAGGTCTGCTCGAATCTGCCGG-3’), then the second round of PCR products were cloned into pJH1005 (digested by *Mlu*I/*Bam*HI) by Gibson assembly.	This work.
pJH1531	O_{comX1}-driven SEAP expression vector. (O_{comX1}-$P_{hCMVmin}$-SEAP-pA).<br>The fragment was PCR-amplified from pJH1005 with OJH1531-GF (5’-GGATCGGGAGATCTCCAC GCGTATTAGCTACTAGTGACATATATGTCTCTAACACTAACTCGAGGGTAGGCGTGTACG	This work.

	-3’) and SEAP-BB3-GR (5’-AGCTTTCTAGACACCGGTGGATCCCTAGGTCTGCTCGAATCTG CCGG-3’), and cloned into pJH1005 (digested by *Mlu*I/*Bam*HI) by Gibson assembly.	
pJH1532	O_{comX2}-driven SEAP expression vector. (O_{comX2}-$P_{hCMVmin}$-SEAP-pA).<br>The fragment was firstly PCR-amplified from pJH1005 with OJH1530-GF1 (5’-GTCTCTAACACTA AATTAGCTACTAGTGACATATATGTCTCTAACACTAACTCGAGGGTAGGCGTGTACG-3’) and SEAP-BB3-GR (5’-AGCTTTCTAGACACCGGTGGATCCCTAGGTCTGCTCGAATCTGCCG G-3’), then the PCR products were taken as the template in the second round of PCR-amplification using OJH1530-GF2 (5’-GGATCGGGAGATCTCCACGCGTATTAGCTACTAGTGACATATATG TCTCTAACACTAAATTAGCTACTAGTGACAT-3’) and SEAP-BB3-GR (5’-AGC TTTCTAGAC ACCGGTGGATCCCTAGGTCTGCTCGAATCTGCCGG-3’), then the second round of PCR products were cloned into pJH1005 (digested by *Mlu*I/*Bam*HI) by Gibson assembly.	This work.
pJH1537	O_{comS3}-driven SEAP expression vector. (O_{comS3}-$P_{hCMVmin}$-SEAP-pA).<br>The fragment was PCR-amplified from pJH1530 with OJH1537-GF (5’-ATCGGGAGATCTCCACGC GTGTAGACTGGAGACACGTAGTGACATTTATGTCACTACACGCGTATTAGCTACCAGTGA C-3’) and SEAP-BB3-GR (5’-AGCTTTCTAGACACCGGTGGATCCCTAGGTCTGCTCGAATCT GCCGG-3’), and cloned into pJH1005 (digested by *Mlu*I/*Bam*HI) by Gibson assembly.	This work.
pJH1538	O_{comS4}-driven SEAP expression vector. (O_{comS4}-$P_{hCMVmin}$-SEAP-pA).<br>The fragment was PCR-amplified from pJH1537 with OJH1538-GF (5’-ATCGGGAGATCTCCACGC GTCTGGTCATACGTGCTCTAGTGACATTTATGTCACTAGTAGACTGGAGACACGTAGTGA	This work.

	C-3') and SEAP-BB3-GR (5'-AGCTTTCTAGACACCGGTGGATCCCTAGGTCTGCTCGAATCT GCCGG-3'), and cloned into pJH1005 (digested by *Mlu*I/*Bam*HI) by Gibson assembly.	
pJH1539	O_{comS5}-driven SEAP expression vector. (O_{comS5}-$P_{hCMVmin}$-SEAP-pA).<br><br>The fragment was PCR-amplified from pJH1538 with OJH1539-GF (5'-ATCGGGAGATCTCCACGC GTCGACTGATACGTGCTCTAGTGACATTTATGTCACTACTGGTCATACGTGCTCTAGTGA C-3') and SEAP-BB3-GR (5'-AGCTTTCTAGACACCGGTGGATCCCTAGGTCTGCTCGAATCT GCCGG-3'), and cloned into pJH1005 (digested by *Mlu*I/*Bam*HI) by Gibson assembly.	This work.
pJH1540	O_{comS6}-driven SEAP expression vector. (O_{comS6}-$P_{hCMVmin}$-SEAP-pA).<br><br>The fragment was PCR-amplified from pJH1539 with OJH1540-GF (5'-ATCGGGAGATCTCCACGC GTCATAGCAGAGAGAGCGTAGTGACATTTATGTCACTACGACTGATACGTGCTCTAGTGA C-3') and SEAP-BB3-GR (5'-AGCTTTCTAGACACCGGTGGATCCCTAGGTCTGCTCGAATCT GCCGG-3'), and cloned into pJH1005 (digested by *Mlu*I/*Bam*HI) by Gibson assembly.	This work.
pJH1541	O_{comS7}-driven SEAP expression vector. (O_{comS7}-$P_{hCMVmin}$-SEAP-pA).<br><br>The fragment was PCR-amplified from pJH1540 with OJH1541-GF (5'-ATCGGGAGATCTCCACGC GTAGGAGAACGGTCGAAGTAGTGACATTTATGTCACTACATAGCAGAGAGAGCGTAGTG AC-3') and SEAP-BB3-GR (5'-AGCTTTCTAGACACCGGTGGATCCCTAGGTCTGCTCGAATC TGCCGG-3'), and cloned into pJH1005 (digested by *Mlu*I/*Bam*HI) by Gibson assembly.	This work.
pJH1542	O_{comS8}-driven SEAP expression vector. (O_{comS8}-$P_{hCMVmin}$-SEAP-pA).	This work.

	The fragment was PCR-amplified from pJH1541 with OJH1542-GF (5'-ATCGGGAGATCTCCACGC GTCTGCAGGTAGAGATCGTAGTGACATTTATGTCACTAAGGAGAACGGTCGAAGTAGTG AC-3') and SEAP-BB3-GR (5'-AGCTTTCTAGACACCGGTGGATCCCTAGGTCTGCTCGAATC TGCCGG-3'), and cloned into pJH1005 (digested by *Mlu*I/*Bam*HI) by Gibson assembly.	
pJH1543	O_{comS9}-driven SEAP expression vector. (O_{comS9}-$P_{hCMVmin}$-SEAP-pA). The fragment was PCR-amplified from pJH1542 with OJH1543-GF (5'-ATCGGGAGATCTCCACGC GTGGATACGAGTCGAGGTTAGTGACATTTATGTCACTACTGCAGGTAGAGATCGTAGTGA C-3') and SEAP-BB3-GR (5'-AGCTTTCTAGACACCGGTGGATCCCTAGGTCTGCTCGAATCT GCCGG-3'), and cloned into pJH1005 (digested by *Mlu*I/*Bam*HI) by Gibson assembly.	This work.
pJH1544	O_{comS10}-driven SEAP expression vector. (O_{comS10}-$P_{hCMVmin}$-SEAP-pA). The fragment was PCR-amplified from pJH1543 with OJH1544-GF (5'-ATCGGGAGATCTCCACGC GTCGAGATCGACGCTGTATAGTGACATTTATGTCACTAGGATACGAGTCGAGGTTAGTGA C-3') and SEAP-BB3-GR (5'-AGCTTTCTAGACACCGGTGGATCCCTAGGTCTGCTCGAATCT GCCGG-3'), and cloned into pJH1005 (digested by *Mlu*I/*Bam*HI) by Gibson assembly.	This work.
pJH1555	Mammalian constitutive $ComR_{C17S}$-VPR expression vector. (P_{hCMV}-$ComR_{C17S}$-VPR-pA). The fragment was PCR-amplified from pJH1522 with OJH1555-GF (5'-AGACCCAAGCTGTTCGAA GCGGAATTCACCATGTCTATCAAGGACTCCATCGGACTGAGAATCAAGACCGAGAGAGA	This work.

	GAGCCAGCAGA-3') and OJH1504-GR (5'-AGCCCGTCCGGAACCACTAGTCAGGCCATCAGC GGCCTTC-3'), and cloned into pJH51 (digested by *Eco*RI/*Spe*I) by Gibson assembly.	
pJH1556	Mammalian constitutive $ComR_{C26S}$-VPR expression vector. (P_{hCMV}-$ComR_{C26S}$-VPR-pA).<br><br>The fragment was firstly PCR-amplified from pJH1522 with OJH1556-GF1 (5'-GGACTGAGAATCA AGACCGAGAGAGAGTGCCAGCAGATGAGCAGAGAAGTGTTAAGTCTGG-3') and OJH1504-GR (5'-AGCCCGTCCGGAACCACTAGTCAGGCCATCAGCGGCCTTC-3'), then the PCR products were taken as the template in the second round of PCR-amplification using OJH1556-GF2 (5'-AAGCTGTTCGAAGCGGAATTCACCATGTCTATCAAGGACTCCATCGGACTGAGAATC AGACCGAGAG-3') and OJH1504-GR (5'-AGCCCGTCCGGAACCACTAGTCAGGCCATCAGC GGCCTTC-3'), after that the second round of PCR products were cloned into pJH51 (digested by *Eco*RI/*Spe*I) by Gibson assembly.	This work.
pJH1557	Mammalian constitutive $ComR_{C17-26S}$-VPR expression vector. (P_{hCMV}-$ComR_{C17-26S}$-VPR-pA).<br><br>The fragment was firstly PCR-amplified from pJH1522 with OJH1557-GF1 (5'-CGGACTGAGAATC AAGACCGAGAGAGAGAGCCAGCAGATGAGCAGAGAAGTGTTAAGTCTGG-3') and OJH1504-GR (5'-AGCCCGTCCGGAACCACTAGTCAGGCCATCAGCGGCCTTC-3'), then the PCR products were taken as the template in the second round of PCR-amplification using OJH1556-GF2 (5'-AAGCTGTTCGAAGCGGAATTCACCATGTCTATCAAGGACTCCATCGGACTGAGA ATCAGACCGAGAG-3') and OJH1504-GR (5'-AGCCCGTCCGGAACCACTAGTCAGGCCATCA	This work.

	GCGGCCTTC-3’), after that the second round of PCR products were cloned into pJH51 (digested by *Eco*RI/*Spe*I) by Gibson assembly.	
pJH1567	Mammalian constitutive JAK/STAT-ComR$_{EXTRA}$ expression vector.<br>(P$_{SV40}$-SP-ComR-EpoR-TM-IL-6RB-pA).<br>The fragment was PCR-amplified from pJH1522 with OJH1567-GF (5’-CTGGATCAACGGGGGACGGATCCTCTATCAAGGACTCCATCGGACTG-3’) and OJH1567-GR (5’-GCTGGGTGAAGGTGCGAATTCTCCGGACAGGCCATCAGCGGCCTTC-3’), and cloned into pLeo619 (digested by *Bam*HI/*Bsp*EI) by Gibson assembly.	This work.
pJH1568	Mammalian constitutive MAPK-ComR$_{EXTRA}$ expression vector.<br>(P$_{SV40}$-SP-ComR-EpoR-FGFR1-pA).<br>The fragment was firstly PCR-amplified from pJH1522 with OJH1568-GF1 (5’-TGCTGCTCTGGGTCCTGCTGCTGTGGGTCCCTGGATCAACGGGTGACGGTCATATGTCTATCAAGGACTCCATCGGACTG-3’) and OJH1568-GR (5’-AGGCTGGGTGAAGGTGCGAATTCACCGGACAGGCCATCAGCGGCCTTC-3’), then the PCR products were taken as the template in the second round of PCR-amplification using OJH1568-GF2 (5’-CTAGGCTTTTGCAAAAAGCTTCGATACGCGCTAGCGGCCGGCCACCATGGAAACTGATACTTTGCTGCTCTGGGTCCTGC-3’) and OJH1568-GR (5’-AGGCTGGGTGAAGGTGCGAATTCACCGGACAGGCCATCAGCGGCCTTC-3’), then the second round of PCR products were cloned into pLeo628 (digested by *Hin*dIII/*Eco*RI) by Gibson assembly.	This work.

pJH1569	Mammalian constitutive VEGFR2-ComR$_{EXTRA}$ expression vector.<br>(P$_{SV40}$-SP-ComR-EpoR-VEGFR2-pA).<br>The fragment was firstly PCR-amplified from pJH1522 with OJH1569-GF1 (5'-TGCTGCTCTGGGTCCTGCTGCTGTGGGTCCCTGGATCAACGGGGGACGGTCATATGTCTATCAAGGACTCCATCGGACTG-3') and OJH1569-GR (5'-AGGCTGGGTGAAGGTGCACTAGTACCGCCCAGGCCATCAGCGGCCTTC-3'), then the PCR products were taken as the template in the second round of PCR-amplification using OJH1568-GF2 (5'-CTAGGCTTTTGCAAAAAGCTTCGATACGCGCTAGCGGCCGGCCACCATGGAAACTGATACTTTGCTGCTCTGGGTCCTGC-3') and OJH1569-GR (5'-AGGCTGGGTGAAGGTGCACTAGTACCGCCCAGGCCATCAGCGGCCTTC-3'), then the second round of PCR products were cloned into pLeo690 (digested by *Hin*dIII/*Spe*I) by Gibson assembly.	This work.
pJH1577	Mammalian reporter plasmid for STAT3-induced SEAP expression in BB3 vector.<br>(O$_{STAT3}$-P$_{hCMVmin}$-SEAP-pA).<br>The fragment was PCR-amplified from pLS13 with OJH1577-GF (5'-ATCGGGAGATCTCCACGCGTCACATTTCCCCGAAAAGTGCCAC-3') and OJH1577-GR (5'-GCTTTCTAGACACCGGTGGATCCGCTAGCGGTCTGCTCGAATCTGCCG-3'), and cloned into pJH1005 (digested by *Mlu*I/*Bam*HI) by Gibson assembly.	This work.
pJH1578	Mammalian constitutive STAT3 expression in BB3 vector.<br>(P$_{hCMV}$-STAT3-pA).	This work.

	The fragment was PCR-amplified from pLS15 with OJH1578-GF (5'-GCTGTTCGAAGCGGAATTC ACCATGGCCCAATGGAATCAGCTACAG-3') and OJH1578-GR (5'-TTCTAGACACCGGTGGA TCCCTACATGGGGGAGGTAGCGCAC-3'), and cloned into pJH3 (digested by *Eco*RI/*Bam*HI) by Gibson assembly.	
pJH1590	Mammalian constitutive JAK/STAT-ComR$_{EXTRA-C17S}$ expression vector.<br>(P$_{SV40}$-SP-ComR$_{C17S}$-EpoR-IL-6RB-pA).<br>The fragment was PCR-amplified from pJH1555 with OJH1567-GF (5'-CTGGATCAACGGGGGAC GGATCCTCTATCAAGGACTCCATCGGACTG-3') and OJH1567-GR (5'-GCTGGGTGAAGGTG CGAATTCTCCGGACAGGCCATCAGCGGCCTTC-3'), and cloned into pJH1567 (digested by *Bam*HI/*Bsp*EI) by Gibson assembly.	This work.
pJH1591	Mammalian constitutive JAK/STAT-ComR$_{EXTRA-C26S}$ expression vector.<br>(P$_{SV40}$-SP-ComR$_{C26S}$-EpoR-IL-6RB-pA).<br>The fragment was PCR-amplified from pJH1556 with OJH1567-GF (5'-CTGGATCAACGGGGGAC GGATCCTCTATCAAGGACTCCATCGGACTG-3') and OJH1567-GR (5'-GCTGGGTGAAGGTG CGAATTCTCCGGACAGGCCATCAGCGGCCTTC-3'), and cloned into pJH1567 (digested by *Bam*HI/*Bsp*EI) by Gibson assembly.	This work.
pJH1592	Mammalian constitutive JAK/STAT-ComR$_{EXTRA-C17-26S}$ expression vector.<br>(P$_{SV40}$-SP-ComR$_{C17-26S}$-EpoR-IL-6RB-pA).	This work.

	The fragment was PCR-amplified from pJH1557 with OJH1567-GF (5'-CTGGATCAACGGGGGACGGATCCTCTATCAAGGACTCCATCGGACTG-3') and OJH1567-GR (5'-GCTGGGTGAAGGTGCGAATTCTCCGGACAGGCCATCAGCGGCCTTC-3'), and cloned into pJH1567 (digested by *Bam*HI/*Bsp*EI) by Gibson assembly.	
pJH1593	Mammalian constitutive JAK/STAT-ComR$_{EXTRA\text{-}F170L}$ expression vector.<br>(P_{SV40}-SP-ComR$_{F170L}$-EpoR-IL-6RB-pA).<br>The fragment was PCR-amplified using an error-prone PCR kit from pJH1567 with OJH1567-GF (5'-CTGGATCAACGGGGGACGGATCCTCTATCAAGGACTCCATCGGACTG-3') and OJH1567-GR (5'-GCTGGGTGAAGGTGCGAATTCTCCGGACAGGCCATCAGCGGCCTTC-3'), and cloned into pJH1567 (digested by *Bam*HI/*Bsp*EI) by Gibson assembly.	This work.
pJH1594	Mammalian constitutive JAK/STAT-ComR$_{EXTRA\text{-}K155R/F158Y/R186S/S222N}$ expression vector.<br>(P_{SV40}-SP-ComR $_{K155R/F158Y/R186S/S222N}$-EpoR-IL-6RB-pA).<br>The fragment was PCR-amplified using an error-prone PCR kit (Agilent: 200550) from pJH1567 with OJH1567-GF (5'-CTGGATCAACGGGGGACGGATCCTCTATCAAGGACTCCATCGGACTG-3') and OJH1567-GR (5'-GCTGGGTGAAGGTGCGAATTCTCCGGACAGGCCATCAGCGGCCTTC-3'), and cloned into pJH1567 (digested by *Bam*HI/*Bsp*EI) by Gibson assembly.	This work.
pJH1595	Mammalian constitutive JAK/STAT-ComR$_{EXTRA\text{-}F170L/K155R/F158Y/R186S/S222N}$ expression vector.<br>(P_{SV40}-SP-ComR $_{F170L/K155R/F158Y/R186S/S222N}$-EpoR-IL-6RB-pA).	This work.

	The fragment one was PCR-amplified from pJH1594 with OJH1567-GF (5'-CTGGATCAACGGGGG ACGGATCCTCTATCAAGGACTCCATCGGACTG-3') and OJH1595-F170L-R (5'-GCTTTCTGC CGTACAGGTGGAGGAAGTAGTAGTCGATC-3'), and fragment two was PCR-amplified from pJH1594 with OJH1595-F170L-F (5'-GATCGACTACTACTTCCTCCACCTGTACGGCAGAAAGC -3') and OJH1567-GR (5'-GCTGGGTGAAGGTGCGAATTCTCCGGACAGGCCATCAGCGGCCT TC-3'), then the two fragments were cloned into pJH1567 (digested by *Bam*HI/*Bsp*EI) by Gibson assembly.	
pJH1596	Mammalian constitutive JAK/STAT-ComR$_{EXTRA-F170L/C17S}$ expression vector.<br>(P$_{SV40}$-SP-ComR$_{F170L/C17S}$-EpoR-IL-6RB-pA).<br>The fragment was PCR-amplified from pJH1593 with OJH1590-GF (5'-CTGGATCAACGGGGGAC GGATCCTCTATCAAGGACTCCATCGGACTGAGAATCAAGACCGAGAGAGAGAGCCAGCA GAT-3') and OJH1567-GR (5'-GCTGGGTGAAGGTGCGAATTCTCCGGACAGGCCATCAGCGG CCTTC-3'), and cloned into pJH1567 (digested by *Bam*HI/*Bsp*EI) by Gibson assembly.	This work.
pJH1597	Mammalian constitutive JAK/STAT-ComR$_{EXTRA-K155R/F158Y/R186S/S222N/C17S}$ expression vector.<br>(P$_{SV40}$-SP-ComR$_{K155R/F158Y/R186S/S222N/C17S}$-EpoR-IL-6RB-pA).<br>The fragment was PCR-amplified from pJH1594 with OJH1590-GF (5'-CTGGATCAACGGGGGAC GGATCCTCTATCAAGGACTCCATCGGACTGAGAATCAAGACCGAGAGAGAGAGCCAGCA GAT-3') and OJH1567-GR (5'-GCTGGGTGAAGGTGCGAATTCTCCGGACAGGCCATCAGCGG CCTTC-3'), and cloned into pJH1567 (digested by *Bam*HI/*Bsp*EI) by Gibson assembly.	This work.

pJH1598	Mammalian constitutive $ComR_{F170L}$-VPR expression vector. (P_{hCMV}-$ComR_{F170L}$-VPR-pA).<br>The fragment was PCR-amplified from pJH1593 with OJH1504-GF (5'-AAGCTGTTCGAAGCGGA ATTCACCATGTCTATCAAGGACTCCATCGGACTGAG-3') and OJH1504-GR (5'-AGCCCGTC CGGAACCACTAGTCAGGCCATCAGCGGCCTTC-3'), and cloned into pJH51 (digested by *Eco*RI/*Spe*I) by Gibson assembly.	This work.
pJH1599	Mammalian constitutive $ComR_{K155R/F158Y/R186S/S222N}$-VPR expression vector.<br>(P_{hCMV}-$ComR_{K155R/F158Y/R186S/S222N}$-VPR-pA).<br>The fragment was PCR-amplified from pJH1594 with OJH1504-GF (5'-AAGCTGTTCGAAGCGGA ATTCACCATGTCTATCAAGGACTCCATCGGACTGAG-3') and OJH1504-GR (5'-AGCCCGTC CGGAACCACTAGTCAGGCCATCAGCGGCCTTC-3'), and cloned into pJH51 (digested by *Eco*RI/*Spe*I) by Gibson assembly.	This work.
pJH1600	Mammalian constitutive $ComR_{F170L}$-VP16 expression vector. (P_{hCMV}-$ComR_{F170L}$-VP16-pA).<br>The fragment was PCR-amplified from pJH1593 with OJH1600-GF (5'-AAGCTGTTCGAAGCGGA ATTCACCATGTCTATCAAGGACTCCATCGGACTGAG-3') and OJH1600-GR (5'-TCGGTCGG GGGGGCTCCGGAACCACTAGTCAGGCCATCAGCGGCCTTC-3'), and cloned into pJH54 (digested by *Eco*RI/*Spe*I) by Gibson assembly.	This work.
pJH1601	Mammalian constitutive $ComR_{K155R/F158Y/R186S/S222N}$-VP16 expression vector.<br>(P_{hCMV}-$ComR_{K155R/F158Y/R186S/S222N}$-VP16-pA).	This work.

	The fragment was PCR-amplified from pJH1594 with OJH1600-GF (5'-AAGCTGTTCGAAGCGGA ATTCACCATGTCTATCAAGGACTCCATCGGACTGAG-3') and OJH1600-GR (5'-TCGGTCGG GGGGGCTCCGGAACCACTAGTCAGGCCATCAGCGGCCTTC-3'), and cloned into pJH54 (digested by *Eco*RI/*Spe*I) by Gibson assembly.	
pJH1602	Mammalian constitutive ComR$_{F170L}$-VP64 expression vector. (P$_{hCMV}$-ComR$_{F170L}$-VP64-pA). The fragment was PCR-amplified from pJH1593 with OJH1504-GF (5'-AAGCTGTTCGAAGCGGA ATTCACCATGTCTATCAAGGACTCCATCGGACTGAG-3') and OJH1504-GR (5'-AGCCCGTC CGGAACCACTAGTCAGGCCATCAGCGGCCTTC-3'), and cloned into pJH55 (digested by *Eco*RI/*Spe*I) by Gibson assembly.	This work.
pJH1603	Mammalian constitutive ComR$_{K155R/F158Y/R186S/S222N}$-VP64 expression vector. (P$_{hCMV}$-ComR$_{K155R/F158Y/R186S/S222N}$-VP64-pA). The fragment was PCR-amplified from pJH1594 with OJH1504-GF (5'-AAGCTGTTCGAAGCGGA ATTCACCATGTCTATCAAGGACTCCATCGGACTGAG-3') and OJH1504-GR (5'-AGCCCGTC CGGAACCACTAGTCAGGCCATCAGCGGCCTTC-3'), and cloned into pJH55 (digested by *Eco*RI/*Spe*I) by Gibson assembly.	This work.
pJH1604	Mammalian constitutive ComR-VP16 expression vector. (P$_{hCMV}$-ComR-VP16-pA). The fragment was PCR-amplified from pJH1522 with OJH1604-GF (5'-AGGGAGACCCAAGCTGTT CGAAGCGGAATTCACCATGTCTATCAAGGACTCCATC-3') and OJH1604-GR (5'-GACATCG	This work.

	GTCGGGGGGCTCCGGAACCACTAGTCAGGCCATCAGCGGCCTTCTC-3’), and cloned into pJH54 (digested by *Eco*RI/*Spe*I) by Gibson assembly.	
pJH1605	O_{STAT3}-driven SEAP and mINS expression vector (O_{STAT3}-SEAP-P2A-mINS-pA).<br><br>The fragment was PCR-amplified from pJH1040 with OJH1605-GF (5’-CCGCGGAATTCACCATG ACTAGTCTGCTGCTGCTGCTGC-3’) and OJH1605-GR (5’-AAAGCTTTCTAGACACCGGTGG ATCCTCAGTTGCAGTAGTTCTCCAGTTGG-3’), and cloned into pJH1577 (digested by *Spe*I/*Bam*HI) by Gibson assembly.	This work.
pJH1606	O_{STAT3}-driven NanoLuc and mINS expression vector. (O_{STAT3}-NanoLuc-P2A-mINS-pA).<br><br>The fragment was PCR-amplified from pJH53 with OJH1606-GF (5’-CCGCGGAATTCACCATGAC TAGTGAGACAGACACACTCC-3’) and OJH1606-GR (5’-GCTTTCTAGACACCGGTGGATCCC TAGTTGCAGTAGTTCTCCAG-3’), and cloned into pJH1577 (digested by *Spe*I/*Bam*HI) by Gibson assembly.	This work.
pJH1607	O_{STAT3}-driven shGLP-1 and NanoLuc expression vector. (O_{STAT3}-shGLP-1-P2A-NanoLuc-pA).<br><br>The fragment was PCR-amplified from pJH1043 with OJH1607-GF (5’-CGCGGAATTCACCATGAC TAGTAAGATCATCCTGTGGCTGT-3’) and OJH1607-GR (5’-TTCTAGACACCGGTGGATCCCT ACGCCAGAATGCGTTCGC-3’), and cloned into pJH1577 (digested by *Spe*I/*Bam*HI) by Gibson assembly.	This work.
pJH1608	O_{comS9}-driven SEAP and mINS expression vector. (O_{comS9}-SEAP-P2A-mINS-pA).	This work.

	The fragment was PCR-amplified from pJH1040 with OJH1605-GF (5'-CCGCGGAATTCACCATGACTAGTCTGCTGCTGCTGCTGC-3') and OJH1605-GR (5'-AAAGCTTTCTAGACACCGGTGGATCCTCAGTTGCAGTAGTTCTCCAGTTGG-3'), and cloned into pJH1543 (digested by *Spe*I/*Bam*HI) by Gibson assembly.	
pJH1609	O_{comS9}-driven NanoLuc and mINS expression vector. (O_{comS9}-NanoLuc-P2A-mINS-pA).<br>The fragment was PCR-amplified from pJH53 with OJH1606-GF (5'-CCGCGGAATTCACCATGACTAGTGAGACAGACACACTCC-3') and OJH1606-GR (5'-GCTTTCTAGACACCGGTGGATCCCTAGTTGCAGTAGTTCTCCAG-3'), and cloned into pJH1543 (digested by *Spe*I/*Bam*HI) by Gibson assembly.	This work.
pJH1610	O_{comS9}-driven shGLP-1 and NanoLuc expression vector. (O_{comS9}-shGLP-1-P2A-NanoLuc-pA).<br>The fragment was PCR-amplified from pJH1043 with OJH1607-GF (5'- CGCGGAATTCACCATGACTAGTAAGATCATCCTGTGGCTGT-3') and OJH1607-GR (5'-TTCTAGACACCGGTGGATCCCTACGCCAGAATGCGTTCGC-3'), and cloned into pJH1543 (digested by *Spe*I/*Bam*HI) by Gibson assembly.	This work.
pJH1611	SB100X-specific transposon containing O_{STAT3}-driven SEAP and mINS expression unit and a constitutive ECFP and PuroR expression unit vector.<br>(ITR-O_{STAT3}-SEAP-P2A-mINS-pA:P_{RPBSA}-ECFP-P2A-PuroR-pA-ITR).	This work.

	The fragment was PCR-amplified from pJH1605 with OJH1611-GF (5'-CTAGTCTTAAGAGATCTACGCGTCACATTTCCCCGAAAAG -3') and OJH1611-GR (5'-CTGCAGGCCGGCCTCAAAGCTTTCTAGACACCGGTGGATC-3'), and cloned into BB6-PuroR (digested by *Mlu*I/*Hind*III) by Gibson assembly.	
pJH1612	SB100X-specific transposon containing O_{STAT3}-driven NanoLuc and mINS expression unit and a constitutive ECFP and PuroR expression unit vector.<br>(ITR-O_{STAT3}-NanoLuc-P2A-mINS-pA:P_{RPBSA}-ECFP-P2A-PuroR-pA-ITR).<br>The fragment was PCR-amplified from pJH1606 with OJH1611-GF (5'-CTAGTCTTAAGAGATCTACGCGTCACATTTCCCCGAAAAG-3') and OJH1611-GR (5'-CTGCAGGCCGGCCTCAAAGCTTTCTAGACACCGGTGGATC-3'), and cloned into BB6-PuroR (digested by *Mlu*I/*Hind*III) by Gibson assembly.	This work.
pJH1613	SB100X-specific transposon containing O_{STAT3}-driven shGLP-1 and NanoLuc expression unit and a constitutive ECFP and PuroR expression unit vector.<br>(ITR-O_{STAT3}-shGLP-1-P2A-NanoLuc-pA:P_{RPBSA}-ECFP-P2A-PuroR-pA-ITR).<br>The fragment was PCR-amplified from pJH1607 with OJH1611-GF (5'-CTAGTCTTAAGAGATCTACGCGTCACATTTCCCCGAAAAG -3') and OJH1611-GR (5'-CTGCAGGCCGGCCTCAAAGCTTTCTAGACACCGGTGGATC-3'), and cloned into BB6-PuroR (digested by *Mlu*I/*Hind*III) by Gibson assembly.	This work.

pJH1618	SB100X-specific transposon containing mammalian constitutive STAT3 expression unit and a constitutive BlastR and iRFP expression unit vector.<br>(ITR-P_{hCMV}-STAT3-pA:P_{RPBSA}-ECFP-P2A-PuroR-pA-ITR).<br>The fragment was PCR-amplified from pJH1578 with OJH1611-GF (5'-CTAGTCTTAAGAGATCT ACGCGTCACATTTCCCCGAAAAG -3') and OJH1611-GR (5'-CTGCAGGCCGGCCTCAAAGCT TTCTAGACACCGGTGGATC-3'), and cloned into BB6-BlastR (digested by *Mlu*I/*Hind*III) by Gibson assembly.	This work.
pJH1620	Mammalian constitutive $ComR_{C17S}$-VPR expression vector. (P_{hCMV}-$ComR_{C17S}$-VPR-pA).<br>The fragment was PCR-amplified from pJH1590 with OJH1620-GF (5'-ACCCAAGCTGTTCGAAG CGGAATTCACCATGTCTATCAAGGACTCC-3') and OJH1620-GR (5'-ATGCGTCAGCCCGTC CGGAACCACTAGTCAGGCCATCAGCGGCC-3'), and cloned into pJH51 (digested by *Eco*RI/ *Spe*I) by Gibson assembly.	This work.
pJH1621	Mammalian constitutive $ComR_{C26S}$-VPR expression vector. (P_{hCMV}-$ComR_{C26S}$-VPR-pA).<br>The fragment was PCR-amplified from pJH1591 with OJH1620-GF (5'-ACCCAAGCTGTTCGA AGCGGAATTCACCATGTCTATCAAGGACTCC-3') and OJH1620-GR (5'-ATGCGTCAGCCCG TCCGGAACCACTAGTCAGGCCATCAGCGGCC-3'), and cloned into pJH51 (digested by *Eco*RI/*Spe*I) by Gibson assembly.	This work.
pJH1622	Mammalian constitutive $ComR_{C17-26S}$-VPR expression vector. (P_{hCMV}-$ComR_{C17-26S}$-VPR-pA).	This work.

	The fragment was PCR-amplified from pJH1592 with OJH1620-GF (5'-ACCCAAGCTGTTCGAAGC GGAATTCACCATGTCTATCAAGGACTCC-3') and OJH1620-GR (5'-ATGCGTCAGCCCGTCC GGAACCACTAGTCAGGCCATCAGCGGCC-3'), and cloned into pJH51 (digested by *Eco*RI/*Spe*I) by Gibson assembly.	
pJH1623	SB100X-specific transposon containing O_{comS9}-driven SEAP and mINS expression unit and a constitutive ECFP and PuroR expression unit vector.<br>(ITR-O_{comS9}-SEAP-P2A-mINS-pA:P_{RPBSA}-ECFP-P2A-PuroR-pA-ITR).<br>The fragment was PCR-amplified from pJH1608 with OJH1611-GF (5'-CTAGTCTTAAGAGATCTA CGCGTCACATTTCCCCGAAAAG-3') and OJH1611-GR (5'-CTGCAGGCCGGCCTCAAAGCTT TCTAGACACCGGTGGATC-3'), and cloned into BB6-PuroR (digested by *Mlu*I/*Hind*III) by Gibson assembly.	This work.
pJH1624	SB100X-specific transposon containing O_{comS9}-driven NanoLuc and mINS expression unit and a constitutive ECFP and PuroR expression unit vector.<br>(ITR-O_{comS9}-NanoLuc-P2A-mINS-pA:P_{RPBSA}-ECFP-P2A-PuroR-pA-ITR).<br>The fragment was PCR-amplified from pJH1609 with OJH1611-GF (5'-CTAGTCTTAAGAGATCT ACGCGTCACATTTCCCCGAAAAG-3') and OJH1611-GR (5'-CTGCAGGCCGGCCTCAAAGCT TTCTAGACACCGGTGGATC-3'), and cloned into BB6-PuroR (digested by *Mlu*I/*Hind*III) by Gibson assembly.	This work.

pJH1629	Mammalian constitutive JAK/STAT-ComR$_{EXTRA-K155R/F158Y/R186S/S222N/C17S}$ expression vector.<br>(P$_{hCMV}$-SP-ComR$_{K155R/F158Y/R186S/S222N/C17S}$-EpoR-IL-6RB-pA).<br>The fragment was PCR-amplified from pJH3 with OJH1629-GF (5'-CGATGTACGGGCCAGATATA CGCGTGGTACCCTCGAGttg-3') and OJH1629-GR (5'-GTGGGAGGTCTATATAAGCAGAGCT CAAGCTTCGAATCGCGCTAGCG-3'), and cloned into pJH1597 (digested by *Mlu*I/*Hind*III) by Gibson assembly.	This work.
pJH1632	SB100X-specific transposon containing a constitutive JAK/STAT-ComR$_{EXTRA-K155R/F158Y/R186S/S222N/C17S}$ expression unit and a constitutive ZeoR and mRuby expression unit vector.<br>(ITR-P$_{SV40}$-SP-ComR$_{K155R/F158Y/R186S/S222N/C17S}$-EpoR-IL-6RB-pA: P$_{hCMV}$-ZeoR-P2A-mRuby-pA-ITR).<br>The fragment was PCR-amplified from pJH1597 with OJH1632-GF (5'-ATCTAGTCTTAAGAGATC TACGCGTGATCTGCGATCTGCATCTCAATTAGTC-3') and OJH1632-GR (5'-GGCGGCTACAT GCCTCAGTAGAAGCTTTGAGGCCGGCCTGCAGGCC-3'), and cloned into BB6-ZeoR (digested by *Mlu*I/*Hind*III) by Gibson assembly.	This work.
pJH1635	SB100X-specific transposon containing a constitutive JAK/STAT-ComR$_{EXTRA-K155R/F158Y/R186S/S222N/C17S}$ expression unit and a constitutive ZeoR and mRuby expression unit vector.<br>(ITR-P$_{hCMV}$-SP-ComR$_{K155R/F158Y/R186S/S222N/C17S}$-EpoR-IL-6RB-pA: P$_{hCMV}$-ZeoR-P2A-mRuby-pA-ITR).	This work.

	The fragment was PCR-amplified from pJH1597 with OJH1632-GF (5'-ATCTAGTCTTAAGAGAT CTACGCGTGATCTGCGATCTGCATCTCAATTAGTC-3') and OJH1632-GR (5'- GGCGGCTA CATGCCTCAGTAGAAGCTTTGAGGCCGGCCTGCAGGCC-3'), and cloned into BB6-ZeoR (digested by *Mlu*I/*Hind*III) by Gibson assembly.	
pJH1637	SB100X-specific transposon containing mammalian constitutive ComR-VPR expression unit and a constitutive BlastR and iRFP expression unit vector.<br>(ITR-P_{hCMV}-ComR-VPR-pA:P_{RPBSA}-ECFP-P2A-PuroR-pA-ITR).<br>The fragment was PCR-amplified from pJH1522 with OJH1611-GF (5'-CTAGTCTTAAGAGATC TACGCGTCACATTTCCCCGAAAAG -3') and OJH1611-GR (5'-CTGCAGGCCGGCCTCAAAGC TTTCTAGACACCGGTGGATC-3'), and cloned into BB6-BlastR (digested by *Mlu*I/*Hind*III) by Gibson assembly.	This work.
pJH1640	Mammalian constitutive SP-NSP14 expression vector.<br>(P_{hCMV}-SP-NSP14-pA).<br>The fragment was PCR-amplified from pET11a_3xFlag-nsp14 with OJH1640-GF (5'- AGCTGTTCG AAGCGGAATTCACCATGGGTCTGCTGCTGCTGCTGGGCCTGAGACTGCAGCTGAGCC TGGGCATCACTAGTGTTGCAACCCTGCAGGCAG-3') and OJH1640-GR (5'-GCTTTCTAGAC ACCGGTGGATCCCTGCAGACGTGTAAAGGTGTTCC-3'), and cloned into pJH3 (digested by *Eco*RI/*Bam*HI) by Gibson assembly.	This work.

pJH1644	SB100X-specific transposon containing O_{comS9}-driven shGLP-1 and NanoLuc expression unit and a constitutive ZeoR and mRuby expression unit vector.<br>(ITR-O_{comS9}-shGLP-1-P2A-NanoLuc-pA: P_{hCMV}-ZeoR-P2A-mRuby-pA-ITR).<br>The fragment was PCR-amplified from pJH1610 with OJH1644-GF (5'-ATCTAGTCTTAAGAGATC TACGCGTGGATACGAGTCGAGG -3') and OJH1644-GR (5'- CTGCAGGCCGGCCTCAAAGCT TTCTAGACACCGGTGGATC-3'), and cloned into BB6-ZeoR (digested by *Mlu*I/*Hind*III) by Gibson assembly.	This work.
pJH1645	Mammalian constitutive JAK/STAT-$ComR_{EXTRA-K155R/F158Y/R186S/S222N/C17S}$ expression vector.<br>(P_{SV40}-SP-$ComR_{K155R/F158Y/R186S/S222N/C17S}$-GS1-TM-IL-6RB-pA).<br>The fragment was PCR-amplified from pJH1597 with OJH1645-GF (5'- CAAAAAGCTTCGAATCG CGCTAGCGGCCGGCCACCATGGAAAC-3') and OJH1645-GR (5'- GATGAGAGGGTCCAGGT CGCTTCCGGACAGGCCATCAGCG-3'), and cloned into pJH1597 (digested by *Nhe*I) by Gibson assembly.	This work.
pJH1646	Mammalian constitutive JAK/STAT-$ComR_{EXTRA-K155R/F158Y/R186S/S222N/C17S}$ expression vector.<br>(P_{SV40}-SP-$ComR_{K155R/F158Y/R186S/S222N/C17S}$-GS2-TM-IL-6RB-pA).<br>The fragment was PCR-amplified from pJH1597 with OJH1645-GF (5'-CAAAAAGCTTCGAATCG CGCTAGCGGCCGGCCACCATGGAAAC-3') and OJH1646-GR (5'- TGAGAGGGTCCAGGTCG	This work.

	CTTCCGCCTCCGGACAGGCCATCAGC-3’), and cloned into pJH1597 (digested by *Nhe*I) by Gibson assembly.	
pJH1647	Mammalian constitutive JAK/STAT-$ComR_{EXTRA-K155R/F158Y/R186S/S222N/C17S}$ expression vector.<br>(P_{SV40}-SP-$ComR_{K155R/F158Y/R186S/S222N/C17S}$-GS3-TM-IL-6RB-pA).<br>The fragment was PCR-amplified from pJH1597 with OJH1645-GF (5’- CAAAAAGCTTCGAATC GCGCTAGCGGCCGGCCACCATGGAAAC-3’) and OJH1647-GR (5’- TGAGAGGGTCCAGGTC GCTTCCGCCGCTTCCTCCGGACAGGCCATCAGC-3’), and cloned into pJH1597 (digested by *Nhe*I) by Gibson assembly.	This work.
pJH1648	Mammalian constitutive JAK/STAT-$ComR_{EXTRA-K155R/F158Y/R186S/S222N/C17S}$ expression vector.<br>(P_{SV40}-SP-$ComR_{K155R/F158Y/R186S/S222N/C17S}$-GS4-TM-IL-6RB-pA).<br>The fragment was PCR-amplified from pJH1597 with OJH1645-GF (5’- CAAAAAGCTTCGAATCG CGCTAGCGGCCGGCCACCATGGAAAC-3’) and OJH1648-GR (5’- TGAGAGGGTCCAGGTCG CTTCCGCCTCCGCTGCCTCCTCCGGACAGGCCATCAGC-3’), and cloned into pJH1597 (digested by *Nhe*I) by Gibson assembly.	This work.
pJH1649	Mammalian constitutive JAK/STAT-$ComR_{EXTRA-K155R/F158Y/R186S/S222N/C17S}$ expression vector.<br>(P_{SV40}-SP-$ComR_{K155R/F158Y/R186S/S222N/C17S}$-GS5-TM-IL-6RB-pA).<br>The fragment was PCR-amplified from pJH1597 with OJH1645-GF (5’- CAAAAAGCTTCGAATCG CGCTAGCGGCCGGCCACCATGGAAAC-3’) and OJH1649-GR (5’-TGAGAGGGTCCAGGTCGC	This work.

	TTCCGCCTCCGCCGCTGCCGCCTCCTCCGGACAGGCCATCAGC-3'), and cloned into pJH1597 (digested by *Nhe*I) by Gibson assembly.	
pJH1650	Mammalian constitutive JAK/STAT-ComR$_{EXTRA-K155R/F158Y/R186S/S222N/C17S}$ expression vector. (P$_{SV40}$-SP-ComR$_{K155R/F158Y/R186S/S222N/C17S}$-GS6-TM-IL-6RB-pA). The fragment was PCR-amplified from pJH1597 with OJH1645-GF (5'- CAAAAAGCTTCGAATCG CGCTAGCGGCCGGCCACCATGGAAAC-3') and OJH1650-GR (5'- TGAGAGGGTCCAGGTCG CTTCCGCCTGATCCGCCTCCGCCGCTGCCGCCTCCTCCGGACAGGCCATCAGC-3'), and cloned into pJH1597 (digested by *Nhe*I) by Gibson assembly.	This work.
pJH1679	Mammalian constitutive NanoLuc-GS1-XIP expression vector. (P$_{EF\text{-}1\alpha}$-NanoLuc-GS1-XIP-pA). The fragment was PCR-amplified from pJH53 with OJH1679-GF (5'- TTCGAAGCGGAATTCACCA TGACTAGTGAGACAGACACACTCCTG-3') and OJH1679-GR (5'-CAGGCCGGCCTCAAAGC TTCTAGTAGTAGATCATGAAGAAAGGCACGCCTCCGGAGCCTCCCGCCAGAATGCGTTC GCAC-3'), and cloned into pJH1640 (digested by *Spe*I/*Hin*dIII) by Gibson assembly.	This work.
pJH1680	Mammalian constitutive NanoLuc-GS2-XIP expression vector. (P$_{EF\text{-}1\alpha}$-NanoLuc-GS1-XIP-pA). The fragment was PCR-amplified from pJH1679 with OJH1679-GF (5'- TTCGAAGCGGAATTCACC ATGACTAGTGAGACAGACACACTCCTG-3') and OJH1680-GR (5'-AGGCACGGATCCTCCAC CGCCAGAGCCTCCGGAGCCTCCCGCCAGAATGCGTTCGCAC-3'), and cloned into pJH1640 (digested by *Spe*I/*Bsp*EI) by Gibson assembly.	This work.

pJH1681	Mammalian constitutive SP-EGFP-GS1-XIP expression vector.<br>(P_{hCMV}-SP-EGFP-GS1-XIP-pA).<br>The fragment was PCR-amplified from 2040 with OJH1681-GF (5'- CAGCTGAGCCTGGGCATCAC TAGTGGTGGTTCTGGTGTGAGCAAG-3') and OJH1681-GR (5'- AGGCCGGCCTCAAAGCTTC TAGTAGTAGATCATGAAGAAAGGCACGCCTCCGGAGCCTCCCTTGTACAGCTCGTCC-3'), and cloned into pJH2040 (digested by *Spe*I/*Hin*dIII) by Gibson assembly.	This work.
pJH1682	Mammalian constitutive SP-EGFP-GS2-XIP expression vector. (P_{hCMV}-SP-EGFP-GS2-XIP-pA).<br>The fragment was PCR-amplified from 2040 with OJH1681-GF (5'- CAGCTGAGCCTGGGCATCAC TAGTGGTGGTTCTGGTGTGAGCAAG-3') and OJH1682-GR (5'-CCTCCACCGCCAGAGCCTC CGGAGCCTCCCTTGTACAGCTCGTCCATGCc-3'), and cloned into pJH1681 (digested by *Spe*I/*Bsp*EI) by Gibson assembly.	This work.
pJH1685	Mammalian constitutive JAK/STAT-$ComR_{EXTRA-K155R/F158Y/R186S/S222N/C17S}$ expression vector.<br>(P_{hCMV}-SP-$ComR_{K155R/F158Y/R186S/S222N/C17S}$-GS4-TM-IL-6RB-pA).<br>The fragment was PCR-amplified from pJH3 with OJH1685-GF (5'-ATGTACGGGCCAGATATAC GCGTGGTACCCTCGAGTTGACATTGATTATTG-3') and OJH1685-GR (5'- ATCAGTTTCCAT GGTGGCCGGCCGCTAGCCAGCTTGGGTCTCCCTATAGTGAGTCG-3'), and cloned into pJH1648 (digested by *Mlu*I/*Nhe*I) by Gibson assembly.	This work.
pJH1687	Mammalian constitutive JAK/STAT-$ComR_{EXTRA}$-EGFP expression vector.	This work

	(P_{SV40}-SP-ComR-EGFP-GS4-TM-IL-6RB-pA).<br>The fragment was PCR-amplified from pJH1682 with OJH1687-GF (5'-AAGGCCGCTGATGGCCT GTCCGGAGGCGGTGTGAGCAAGGGCGAGG-3') and OJH1687-GR (5'-GGGTGAAGGTGCGA ATTCTCCGGACTTGTACAGCTCGTCCATGCC-3'), and cloned into pJH1597 (digested by *Bsp*EI) by Gibson assembly.	
pJH1692	Mammalian constitutive JAK/STAT-3xHA-ComR$_{EXTRA}$ expression vector.<br>(P_{SV40}-SP-3xHA-ComR-GS4-TM-IL-6RB-pA).<br>Fragment one was PCR-amplified from pJH1597 with OJH1692-GF1 (5'-GACGTGCCGGACTA TGCGTATCCCTATGACGTCCCGGACTATGCCCATACTAGTATCAAGGACTCCATCGGACT GAG-3') and OJH1692-GR (5'-GCTGGGTGAAGGTGCGAATTCTCCGGACAGGCCATCAGCGG CC-3'), then fragment two was PCR-amplified from fragment one with OJH1692-GF2 (5'-GGATCAACGGGGGACGGATCCTATCCTTATGACGTGCCCGACTATGCCTATCCGTATGAC GTGCCGGACTATGCGTATC-3') and OJH1692-GR (5'-GCTGGGTGAAGGTGCGAATTCTCCG GACAGGCCATCAGCGGCC-3'). Next, fragment two was cloned into pJH1597 (digested by *Bam*HI/*Bsp*EI) by Gibson assembly.	This work
pJH1696	Mammalian constitutive expression vector containing CD19-CAR.<br>(P_{SV40}-SP-3xHA-pSLCAR-CD19-pA).<br>The fragment was PCR-amplified from the template (Addgene no.: 135992) with OJH1696-GF (5'-CCCGGACTATGCCCATACTAGTGACATCCAGATGACACAGACTACATCC-3') and OJH1696-	This work

	GR (5'-AACGGGCCCTCTAGACTCGAGGCTAGCGCGAGGGGGCAG-3'), and cloned into pJH1692 (digested by *Spe*I/*Xho*I) by Gibson assembly.	
pJH2040	Mammalian constitutive SP-EGFP expression vector. (P_{hCMV}-EGFP-pA).<br>The fragment was PCR-amplified from H107 with OJH2040-GF (5'-GCTGGGCCTGAGACTGCA GCTGAGCCTGGGCATCACTAGTGGTGGTTCTGGTGTGAGCAAG-3') and OJH1650-GR (5'-GGCATGGACGAGCTGTACAAGGGATCCACCGGTGTCTAGAAAGCTTTGA-3'), and cloned into pJH1640 (digested by *Spe*I/*Bam*HI) by Gibson assembly.	This work.

Abbreviations:

ARE: antioxidant response element; **BFP**, blue fluorescent protein; **BlastR**, gene conferring blasticidin resistance; **CAR**, Chimeric antigen receptor; **cDNA**, complementary DNA; **CD19**, cluster of differentiation 19 (a cell-surface protein); **CMV**, cytomegalovirus; **CRE**, cAMP-response element; **CREB1**, CAMP-responsive element binding protein 1; **dCas9**, nuclease-deactivated Cas9 endonuclease; **ECFP**, enhanced cyan fluorescent protein; **EGFP**, enhanced green fluorescent protein; **Elk1**, ETS Like-1 transcription factor; **EpoR**, erythropoietin receptor; **$EpoR_{0\text{-}4A}$**, EpoR extracellular and transmembrane domain with 0-4 C-terminal alanines; **EpoRm**, modified EpoR3A (F93A**,** mutation of phenylalanine 93 to alanine (original numbering of full-length EpoR)); **$EpoR_{m0\text{-}4A}$**, modified EpoR0-4A (F93A); **$FGFR1_{int}$**, murine fibroblast growth factor receptor 1 intracellular domain; **FLAG**, FLAG octapeptide tag; **GLP-1**, glucagon-like peptide 1; **GEMS**, generalized extracellular molecule sensor (describes the framework P_{SV40}-SP-VHH_{A52}-$EpoR_m$-IL-$6RB_m$-pA); **GS**, glycine and serine repeats; **IL-6RB**, IL-6 receptor subunit beta; **iRFP**, near-infrared fluorescent protein; **ITR**, inverted terminal repeats of SB100X; **KEAP1**: Kelch-like ECH-associated protein 1; **MAPK**, mitogen-activated protein kinase; **MCS**, multiple cloning site; **mFGFR1**, murine fibroblast growth factor receptor 1; **mINS**, modified insulin variant for optimal expression in HEK-293 cells; **mPGK**: a mouse constitutive promoter; **mRuby**: a bright monomeric red fluorescent protein; **NanoLuc**, *Oplophorus gracilirostris* luciferase; **NemR**: a HTH-type transcriptional repressor from *E. coli*; **NFAT**, nuclear factor of activated T-cells; **NF-**

κB, nuclear factor kappa light chain enhancer of activated B cells; **NRF2**: nuclear factor erythroid 2 p45-related factor 2; O_{STAT3}, STAT3 operator**;** **O_{tetR}**, TetR-specific operator; **P2A**, picornavirus-derived ribosome skipping sequence optimized for bicistronic expression in mammalian cells; **pA**, polyadenylation signal; **PCR**, polymerase chain reaction; **P_{CRE}**, CRE-containing synthetic mammalian promoter; **P_{DART}**, promoter of DC-actuated regulation technology containing ARE element, O_{ARE}-$P_{hCMVmin}$; **P_{DART2}**, O_{ARE2}-$P_{hCMVmin}$; **P_{DART3}**, O_{ARE3}-$P_{hCMVmin}$; **P_{DART4}**, O_{ARE4}-$P_{hCMVmin}$; **$P_{EF\text{-}1\alpha}$**, human elongation factor-1 alpha promoter; **$P_{EF\text{-}1\alpha\text{-}core}$**, core region of EF-1α promotor; **P_{hCMV}**, human cytomegalovirus immediate early promoter; **$P_{hCMVmin}$**, minimal version of P_{hCMV}; **P_{RPBSA}**: a constitutive synthetic mammalian promoter; **P_{SV40}**, simian virus 40 promoter; **PIP**, pristinamycin-induced protein; **P_{TRE}**, O_{TetR}-$P_{hCMVmin}$; **PuroR**, gene conferring puromycin resistance; **RR120**, reactive red 120; **scFv**, single-chain fragment variable; **SB100X**, optimized Sleeping Beauty transposase; **SEAP**, human placental secreted alkaline phosphatase; **shGLP1**, short human glucagon-like peptide 1; **SP**, secretory signal peptide; **STAT3**, signal transducer and activator of transcription 3; **T7**, T7 promoter; **TetR**, *Escherichia coli* Tn10-derived tetracycline-dependent repressor of the tetracycline resistance gene; **VEGFR2**, vascular endothelial growth factor receptor 2, **VH**, variable domain heavy chain, **VHH**, variable domain of camelid heavy chain antibody, **VL**, variable domain light chain; **VP16**, herpes simplex virus protein 16 transactivation domain; **VP64**, a transcriptional activator composed of four tandem copies of VP16; **VPR**, tripartite transcriptional activator consisting of VP64, P65 and Rta; **XIP**, SigX-inducing peptide from *Streptococcus*; **ZeoR**, gene conferring zeocin resistance.

Supplementary Table 2. Primers used for qPCR analysis.

Genes	Forward primers	Reverse primers
SEAP	5'-ATGAATCGGGCCAAGAAAGC-3'	5'-GAGTACCAGTTGCGGTTCAC-3'
Insulin	5'-GGGATCTTCAGACCTTGGCA-3'	5'-TGCAGTAGTTCTCCAGTTGGT-3'
STAT3	5'-GTGGGAAGAATCACGCCTTC-3'	5'-AGATCCTGCACTCTCTTCCG-3'
ComR	5'-CCTGCTGGTGATCGACTACT-3'	5'-GAACAGGGCGATGTTGTAGG-3'
GAPDH (house-keeping gene)	5'-GTCTCCTCTGACTTCAACAGCG-3'	5'-ACCACCCTGTTGCTGTAGCCAA-3'

Supplementary Table 3. Screening peptides.

**Name**	**Peptide sequences**
XIP-WT	VPFFMIYY
TD1	PFFMIYY
TD2	FFMIYY
TD3	FMIYY
TD4	MIYY
TD5	VPFFMIY
TD6	VPFFMI
TD7	VPFFM
TD8	VPFF
TD9	PFFMIY
TD10	PFFMI
TD11	PFFM
TD12	FFMIY
TD13	FMIY
ID1	VPFFMYY
ID2	VPFFIYY
ID3	VPFFYY
ID4	VPFFMY
ID5	VPFIYY
ID6	VPFMYY
ID7	VPFMIY
ID8	VFFMIYY

ID9	VPFMIYY
RE1	LPFFYY
RE2	IPFFYY
RE3	APFFYY
RE4	LPYFCL
RE5	LPYFGCL
RE6	DPFFGVYY
RE7	DPFFDVYY
RE8	DPFLGVYY
RE9	DPFLDVYY
RE10	VPYFMIYY
RE11	APYFMIYY
RE12	APYFMGYY
RE13	APYFMGY
RE14	VPYFMIY

Abbreviations:

XIP: comX-inducing peptide; WT: wild type; TD: terminal residue deletion; ID: internal residue deletion; RE: residue exchange.

Supplementary Table 4. Peptide synthesis costs for mouse experiment in this study.

Peptide Name	Peptide Sequence	Amount (mg)	Cost	Dosage (mg/kg/dose)	Estimated treatment cost per day (2 doses/mouse/day)
XIP_WT	VPFFMIYY	10	$ 69	50	**$ 17.25**
		1000	$ 591.3	50	**$ 1.48**
XIP_ID3	VPFFYY	10	$ 69.2	50	**$ 17.30**
		250	$ 131.4	50	**$ 1.31**

Note. The cost of peptide synthesis depends on the amount required and other factors. This table indicates our costs, calculated for a mouse weighing 25 g. For current synthesis costs, contact the company (https://novoprolabs.com/custom-peptide-synthesis/).

References

1. Huang, J.; Xue, S.; Buchmann, P.; Teixeira, A. P.; Fussenegger, M., *Nature Metabolism* **2023**, 1-13.
2. Keeley, M. B.; Busch, J.; Singh, R.; Abel, T., *BioTechniques* **2005,** *39* (4), 529-536.
3. Bloemberg, D.; Nguyen, T.; MacLean, S.; Zafer, A.; Gadoury, C.; Gurnani, K.; Chattopadhyay, A.; Ash, J.; Lippens, J.; Harcus, D., *Molecular Therapy-Methods & Clinical Development* **2020,** *16*, 238-254.
4. Kemmer, C.; Gitzinger, M.; Daoud-El Baba, M.; Djonov, V.; Stelling, J.; Fussenegger, M., *Nature Biotechnology* **2010,** *28* (4), 355-360.
5. Mátés, L.; Chuah, M. K.; Belay, E.; Jerchow, B.; Manoj, N.; Acosta-Sanchez, A.; Grzela, D. P.; Schmitt, A.; Becker, K.; Matrai, J., *Nature Genetics* **2009,** *41* (6), 753-761.
6. Kowarz, E.; Löscher, D.; Marschalek, R., *Biotechnology Journal* **2015,** *10* (4), 647-653.
7. Chavez, A.; Scheiman, J.; Vora, S.; Pruitt, B. W.; Tuttle, M.; Iyer, E. P.; Lin, S.; Kiani, S.; Guzman, C. D.; Wiegand, D. J., *Nature Methods* **2015,** *12* (4), 326-328.
8. Canal, B.; McClure, A. W.; Curran, J. F.; Wu, M.; Ulferts, R.; Weissmann, F.; Zeng, J.; Bertolin, A. P.; Milligan, J. C.; Basu, S., *Biochemical Journal* **2021,** *478* (13), 2445-2464.
9. Schukur, L.; Geering, B.; Charpin-El Hamri, G.; Fussenegger, M., *Science Translational Medicine* **2015,** *7* (318), 318ra201-318ra201.
10. Scheller, L.; Strittmatter, T.; Fuchs, D.; Bojar, D.; Fussenegger, M., *Nature Chemical Biology* **2018,** *14* (7), 723-729.
11. Mansouri, M.; Hussherr, M.-D.; Strittmatter, T.; Buchmann, P.; Xue, S.; Camenisch, G.; Fussenegger, M., *Nature Communications* **2021,** *12* (1), 3388.
12. Liu, Y.; Bai, P.; Woischnig, A.-K.; Charpin-El Hamri, G.; Ye, H.; Folcher, M.; Xie, M.; Khanna, N.; Fussenegger, M., *Cell* **2018,** *174* (2), 259-270. e11.
13. Fussenegger, M.; Bailey, J. E.; Varner, J., *Nature Biotechnology* **2000,** *18* (7), 768-774.
14. Xie, M.; Ye, H.; Wang, H.; Charpin-El Hamri, G.; Lormeau, C.; Saxena, P.; Stelling, J.; Fussenegger, M., *Science* **2016,** *354* (6317), 1296-1301.
15. Ye, H.; Baba, M. D.-E.; Peng, R.-W.; Fussenegger, M., *Science* **2011,** *332* (6037), 1565-1568.
16. Ye, H.; Xie, M.; Xue, S.; Hamri, G. C.-E.; Yin, J.; Zulewski, H.; Fussenegger, M., *Nature Biomedical Engineering* **2016,** *1* (1), 0005.
17. Xue, S.; Yin, J.; Shao, J.; Yu, Y.; Yang, L.; Wang, Y.; Xie, M.; Fussenegger, M.; Ye, H., *Molecular Therapy* **2017,** *25* (2), 443-455.
